# Structure-based inhibition of acetylcholinesterase and butyrylcholinesterase with 2-Aryl-6-carboxamide benzoxazole derivatives: synthesis, enzymatic assay, and *in silico* studies

**DOI:** 10.1007/s11030-024-10828-6

**Published:** 2024-03-30

**Authors:** Burak Kuzu, M. Abdullah Alagoz, Yeliz Demir, Ilhami Gulcin, Serdar Burmaoglu, Oztekin Algul

**Affiliations:** 1 Department of Pharmaceutical Chemistry, Faculty of Pharmacy, Van Yuzuncu Yil University, Van, 65080 Turkey; 2 Department of Pharmaceutical Chemistry, Faculty of Pharmacy, Mersin University, Mersin, 33169 Turkey; 3 Department of Pharmaceutical Chemistry, Faculty of Pharmacy, İnonu University, Malatya, 44280 Turkey; 4 Department of Pharmacy Services, Nihat Delibalta Göle Vocational High School, Ardahan University, Ardahan, 75000 Turkey; 5 Department of Chemistry, Faculty of Science, Ataturk University, Erzurum, 25240 Turkey; 6 Department of Pharmaceutical Chemistry, Faculty of Pharmacy, Erzincan Binali Yildirim University, Erzincan, 24100 Turkey

**Keywords:** Benzoxazole, Enzyme inhibition, Acetylcholinesterase, Butyrylcholinesterase, Molecular docking and dynamic simulation

## Abstract

An important research topic is the discovery of multifunctional compounds targeting different disease-causing components. This research aimed to design and synthesize a series of 2-aryl-6-carboxamide benzoxazole derivatives that inhibit cholinesterases on both the peripheral anionic and catalytic anionic sides. Compounds (**7**–**48**) were prepared from 4-amino-3-hydroxybenzoic acid in three steps. The Ellman test, molecular docking with Maestro, and molecular dynamics simulation studies with Desmond were done (Schrodinger, 12.8.117). Compound **36**, the most potent compound among the 42 new compounds synthesized, had an inhibitory concentration of IC_50_ 12.62 nM for AChE and IC_50_ 25.45 nM for BChE (whereas donepezil was 69.3 nM and 63.0 nM, respectively). Additionally, compound **36** had docking values ​​of − 7.29 kcal/mol for AChE and − 6.71 kcal/mol for BChE (whereas donepezil was − 6.49 kcal/mol and − 5.057 kcal/mol, respectively). Furthermore, molecular dynamics simulations revealed that compound **36** is stable in the active gorges of both AChE (average RMSD: 1.98 Å) and BChE (average RMSD: 2.2 Å) (donepezil had average RMSD: 1.65 Å and 2.7 Å, respectively). The results show that compound **36** is a potent, selective, mixed-type dual inhibitor of both acetylcholinesterase and butyrylcholinesterase. It does this by binding to both the catalytically active and peripheral anionic sites of cholinesterases at the same time. These findings show that target compounds may be useful for establishing the structural basis for new anti-Alzheimer agents.

**Graphical abstract:**

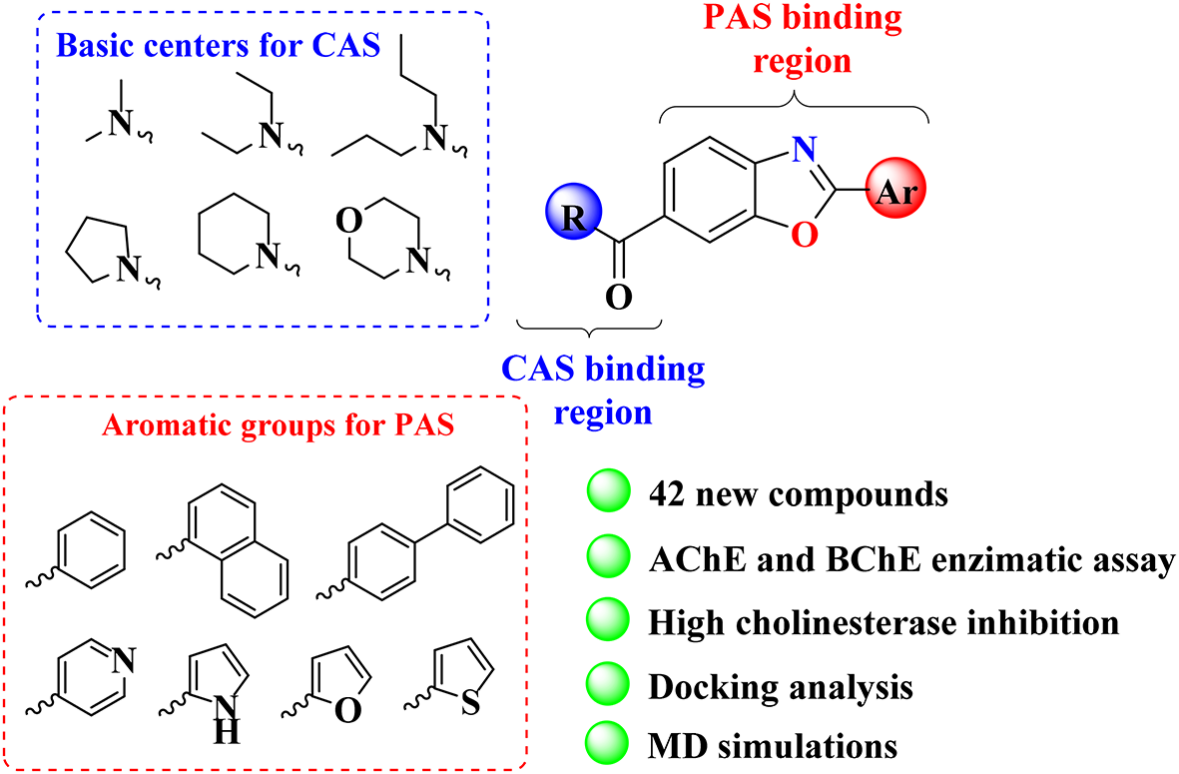

**Supplementary Information:**

The online version contains supplementary material available at 10.1007/s11030-024-10828-6.

## Introduction

Alzheimer’s disease (AD) is a serious health problem that primarily affects the elderly and is the leading cause of dementia and behavioral disorders [1]. The neuronal cortex and hippocampus are the most affected areas of the brain when neurons degenerate in AD [2]. According to the World Health Organization (WHO), 50 million people are affected by this condition, and that number is expected to triple by 2050 [3].

The pathogenesis of AD is not completely understood; typical pathological signs include amyloid-β (Aβ) deposits, neurofibrillary tangles, oxidative stress, and decreased acetylcholine (ACh) levels in the brain [4]. Furthermore, it was discovered that ACh levels increased in patients because of an effective immune response designed to reduce cognitive and behavioral symptoms reported in AD patients [5]. So, over the last two decades, acetylcholinesterase (AChE) has been the focus of intensive pharmaceutical research, resulting in the development of several drugs currently in clinical use for the treatment of AD, with AChE inhibitors constituting many drugs approved by the FDA for the disease [6, 7].

**Fig. 1:**
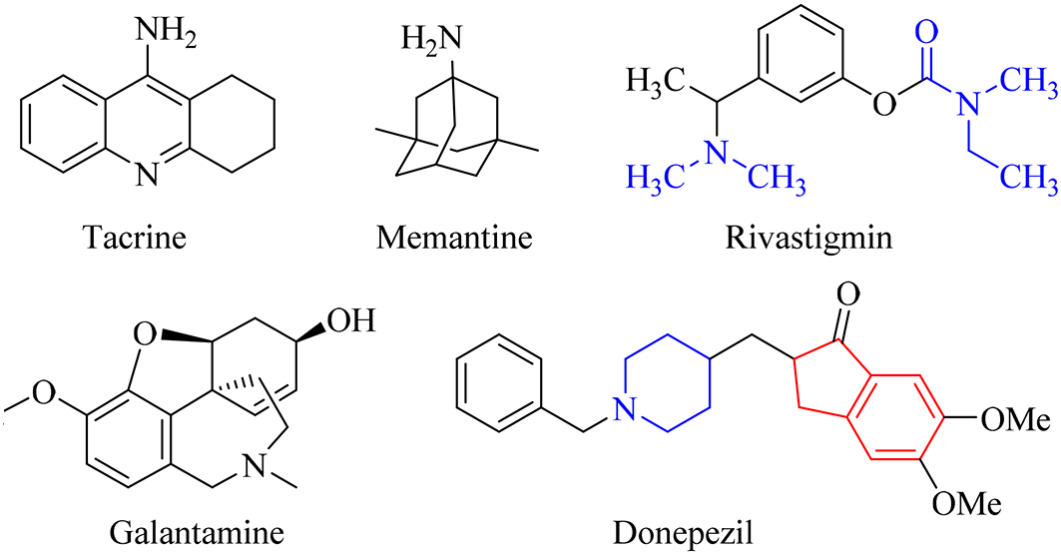
Some FDA-approved anti-Alzheimer drugs

Since 1998, approximately 100 potential anti-Alzheimer drug candidates have been tested in clinical trials, but only 5 drugs have been approved for clinical use by the FDA (Fig. 1). Among these are tacrine [8] with an acridine ring, memantine [9] with an adamantyl group, rivastigmine [10] has an *N*-methyl-*N*-ethyl carbamate functional group, galantamine [11] with an azepane coupled with a benzofuran structure, and donepezil [12] with indene and piperidine. Tacrine, on the other hand, was withdrawn from the market due to the occurrence of severe adverse effects, such as hepatotoxicity, in a significant proportion of patients [13]. Currently, despite the availability of memantine, rivastigmine, galantamine, and donepezil on the market, several studies are being conducted to investigate the problems caused by the overuse of these medications and the numerous negative effects associated with their long-term use [14]. As a result, the development of new drugs for the treatment of AD has become a critical topic in recent years.

Researchers developed a multitarget-directed ligand (MTDL) strategy based on failed clinical trial drug candidates [15]. The evolutionary MTDL technique was used to discover that combining multiple drugs into a single formulation improves therapeutic effects. Several novel hybrid compounds with nanomolar cholinesterase inhibitory activity have been produced using synthetic methods for these drugs [16]. Recent synthetic studies have produced AChE inhibitors such as tacrine-benzofuran [17], tacrine-gallamine [18], tacrine-donepezil [18–21], tacrine-benzothiazole [22], and rivastigmine-benzoxazole [23]. Nonetheless, the discovered MTDL candidate compounds had adverse effects comparable to traditional AChE inhibitors and were shown to inhibit cholinesterase with various additional pharmacological features [24, 25].

Fragment-based approaches have been developed to efficiently produce multi-target drugs [26]. A fragment of cyclic amide, for example, is combined into a single pharmacophore molecule. The amino and carbonyl parts of the cyclic amide group have a unique H-bond network that allows them to inhibit several ATP-competitive kinases in AD [27]. Because recently developed hybrid compounds have side effects comparable to commercial AChE inhibitors, research has focused on developing selective, potent, and reversible AChE inhibitors. In this way, knowledge of protein-ligand interactions could be applied to the development of new drugs.

It is well understood that new ligands compatible with the active site of the AChE enzyme must be designed to create novel AChE inhibitors. Newly designed ligands are known to improve AChE enzyme activity by interacting with both the catalytic anionic site (CAS) and the peripheral anionic binding site (PAS) in the active sites [28]. The aromatic groups in compounds designed to inhibit AChE are chemically linked to the PAS region, whereas the groups bearing the basic center are bound to the CAS [29, 30]. Compounds with dual binding properties, such as donepezil, have an extremely potent AChE inhibition profile [30–34].

**Fig. 2:**
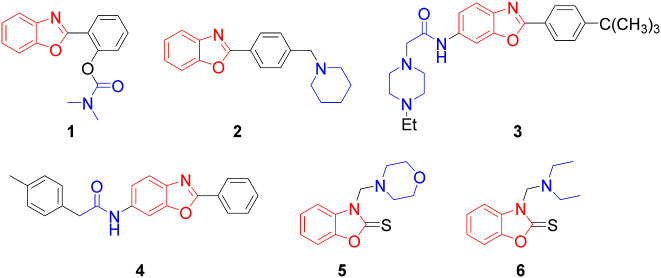
Some benzoxazole derivatives for AChE inhibition

In addition to these, benzo-heterocyclic compounds have been developed, and numerous hybridizations with functional groups can be found in commercially available drugs. The benzoxazole ring structure was chosen as a new scaffold for AChE because it proved to be the most effective. Compound **1** is a benzoxazole derivative with a benzoxazole ring structure derived from thioflavin-T (Fig. 2). The *N,N*-dimethyl carbamate functional group, which mimics the carbamate in rivastigmine, is substituted into the benzoxazole structure of this molecule, and at a concentration of 58.2 nM, it inhibits AChE [23]. The pyrrole and piperidine groups are more effective than the piperazine analogs, with EC_50_ values of 1.03 nM, 1.35 nM, and 62.23 nM, respectively, in compound **2**, where the *p*-position of the 2-phenyl benzoxazole derivatives are substituted with alicyclic amino-methyl derivatives [35]. It has also been discovered that replacing a 2-phenyl group at the *p*-position in the structure increases the activity of several benzoxazole derivatives. Both compound **3**, which contains a piperazine-substituted amide at the 6-position, and compound **4**, which contains a phenylacetamide derivative at the 6-position, were found to be highly potent AChE inhibitors (IC_50_: 3.67 nM and 38.36 nM, respectively) [36, 37]. Compounds **5** (3-morpholino methyl-2-thione benzoxazole, IC_50_: 44 nM) and **6** (3-(diethylamino) methyl-2-thione benzoxazole, IC_50_: 38 nM) were discovered to have potent AChE inhibition activity (Fig. 2) [38].

**Fig. 3:**
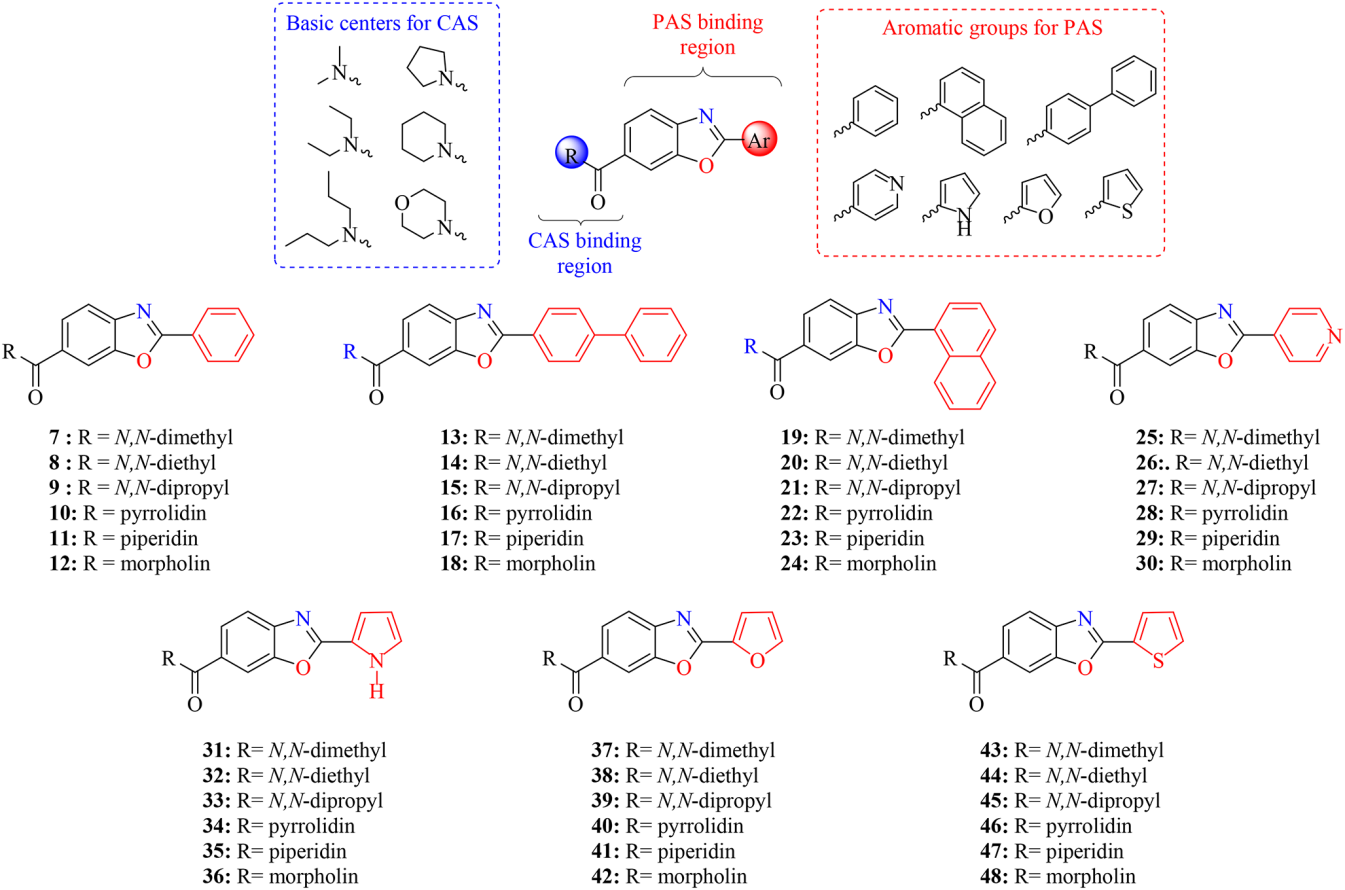
Our designed fragment-based compounds for AChE and BChE inhibition

In this study, we created a series of inhibitors based on new scaffolds by incorporating functional groups that promote cationic, hydrogen bonding, and hydrophobic interactions with conserved and hot-spot residues. We rationally combined benzoxazole and aromatic ring systems with cyclic and acyclic nitrogen amide-containing moieties, which are logically required for effective enzyme-ligand interactions. We developed benzoxazole compounds with aromatic (phenyl, naphthyl, and biphenyl groups) and heteroaromatic (pyridine, pyrrole, furan, and thiophene) groups in the 2-position of benzoxazole to increase PAS binding potential for AChE in our fragment-based strategy. To increase the CAS binding potential for AChE, we modified the acyclic amides (dimethyl, diethyl, and dipropyl) and cyclic amides (pyrrolidine, piperidine, and morpholine) in the 6-position of benzoxazole (Fig. 3).

## Results and discussion

### Chemistry

**Scheme 1:**
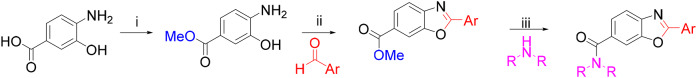
General synthesis method. Reagents and conditions: (i) 1–2 drops H_2_SO_4_, MeOH, reflux (ii) Firstly; EtOH, rt, 6–24 h, Secondly; Catalytic NaCN, DMF, rt, 1 h. (iii) AlCl_3_, DCM, rt, 24 h

In three steps, the target compounds (**7**–**48**) were synthesized from 4-amino-3-hydroxybenzoic acid. In the first step, 4-amino-3-hydroxy-benzoic acid was refluxed in methanol for 12 h with a few drops (5% mol) of concentrated H_2_SO_4_. 4-Amino-3-hydroxybenzoate was successfully obtained in 98% yield after the necessary purification processes. The reaction of 4-amino-3-hydroxybenzoate with related aromatic carboxaldehyde derivatives yielded 2-aryl benzoxazole derivatives in the second step (details are indicated in supporting information). The target compounds were obtained in very high yields in the final step by treating 2-aryl benzoxazole derivatives in DCM and the presence of AlCl_3_ with cyclic or linear aliphatic secondary amines (pyrrolidine, piperidine, morpholine or dimethylamine, diethylamine, and dipropylamine) (Scheme 1).

### Enzyme inhibition study

In the treatment of AD, cholinesterase (ChE) inhibitors are frequently utilized. AChE is a major target for new drug designs for the treatment of AD [39, 40]. Current research indicates that BChE plays a significant role in fiber production and also contributes to the breakdown of acetylcholine, making it an important target for the development of novel drugs for the treatment of AD [41, 42]. The goal of multi-target new drug therapy is to create novel AD medication candidates with anti-cholinesterase, anti-amyloid-β aggregation, and neuroprotective properties. Enzyme inhibitors had a crucial importance in a variety of disease management situations [43]. The inhibition of AChE and BChE by specific inhibitors is an important therapeutic target for controlling AD, Myasthenia gravis, glaucoma, Lewy body, and dementia [44]. Acetylcholinesterase inhibitors (AChEIs) are utilized in clinical practice treating these problems, as they improve cholinergic function by increasing the amount of ACh in cholinergic synapses [45]. In this study, the 2-aryl-6-carboxamide benzoxazole derivatives (**7**–**48**) were investigated in vitro for their ability to inhibit AChE and BChE enzymes. Also, tacrine (TAC) and donepezil were used as positive controls for both cholinergic enzymes. The inhibitory results of the 2-aryl-6-carboxamide benzoxazole derivatives (**7**–**48**) are summarized in Table 1.

The novel synthesized compounds effectively inhibited AChE with IC_50_ values ranging from 12.62 to 67.02 nM and K_i_ values ranging from 7.74 ± 0.75 to 85.33 ± 11.22 nM. The compound of (2-(1 H-pyrrol-2-yl)benzo[d]oxazol-6-yl)(morpholino)methanone (**36**) showed the best inhibition profile (K_i_: 7.74 ± 0.75 nM) (Table 1). As seen in Table 1, all compounds studied for AChE showed more effective inhibition than TAC (K_i_: 95.21 ± 11.87 nM). The CAS binding region and aromatic ring of the benzoxazole group shown in Fig. 3 are changed, and there are also changes in enzyme inhibition.

**Table 1:** Inhibition data of AChE and BChE with the novel series of 2-aryl-6-carboxamide benzoxazole derivatives (**7–48**)

Compounds	IC_50_ (nM)				K_i_ (nM)			Selectivity index (AChE/BChE)
	AChE	r^2^	BChE	r^2^	AChE		BChE	
7	32.88	0.9786	65.77	0.9845	23.04 ± 1.70		50.74 ± 6.43	0.45
8	33.99	0.9713	73.99	0.9713	30.60 ± 3.34		76.95 ± 5.25	0.40
9	48.80	0.9822	116.28	0.9878	59.39 ± 3.69		111.23 ± 4.67	0.53
10	35.68	0.9856	51.35	0.9799	46.53 ± 6.59		36.62 ± 10.97	1.27
11	21.41	0.9843	61.25	0.9816	18.65 ± 2.58		74.38 ± 16.89	0.25
12	29.63	0.9795	34.45	0.9854	28.56 ± 3.96		32.33 ± 3.25	0.88
13	33.23	0.9765	63.46	0.9901	29.82 ± 1.02		65.38 ± 14.05	0.46
14	30.76	0.9815	84.55	0.9845	19.77 ± 3.69		75.25 ± 13.78	0.26
15	40.67	0.9876	113.01	0.9878	38.21 ± 0.66		95.74 ± 7.32	0.40
16	26.33	0.9867	86.25	0.9876	26.70 ± 5.13		84.03 ± 12.45	0.32
17	20.62	0.9889	58.88	0.9813	18.37 ± 3.33		49.18 ± 11.13	0.37
18	21.27	0.9890	65.09	0.9821	20.89 ± 4.22		56.65 ± 1.57	0.37
19	39.58	0.9915	142.35	0.9865	38.75 ± 2.96		121.36 ± 15.45	0.32
20	53.59	0.9954	174.34	0.9901	48.53 ± 9.97		136.03 ± 30.69	0.36
21	67.02	0.9821	188.08	0.9721	71.56 ± 12.01		145.13 ± 11.22	0.50
22	43.05	0.9843	124.22	0.9843	41.91 ± 5.64		72.45 ± 5.66	0.58
23	24.56	0.9902	98.27	0.9744	20.21 ± 1.47		69.37 ± 11.34	0.29
24	50.94	0.9955	83.77	0.9821	45.73 ± 1.65		50.69 ± 14.02	0.90
25	54.66	0.9878	93.59	0.9775	85.33 ± 11.22		66.40 ± 13.58	1.28
26	48.11	0.9741	76.24	0.9815	83.13 ± 1.33		54.39 ± 4.85	1.53
27	49.50	0.9798	87.13	0.9759	50.05 ± 5.13		69.69 ± 4.10	0.72
28	40.51	0.9817	57.17	0.9844	47.92 ± 4.70		52.92 ± 11.67	0.91
29	28.03	0.9832	54.13	0.9835	27.38 ± 2.53	47.90 ± 8.42		0.51
30	24.26	0.9954	49.67	0.9821	18.32 ± 4.37		45.35 ± 3.04	0.40
31	36.75	0.9909	47.33	0.9803	30.70 ± 2.21		41.99 ± 6.32	0.73
32	41.67	0.9943	30.13	0.9924	47.53 ± 9.80		23.01 ± 1.52	2.07
33	40.17	0.9821	55.45	0.9734	26.13 ± 3.35		38.47 ± 8.43	0.68
34	26.68	0.9856	53.16	0.9905	15.51 ± 1.96		48.22 ± 2.56	0.32
35	19.02	0.9735	49.21	0.9876	12.31 ± 1.28		44.46 ± 7.43	0.27
36	12.62	0.9768	25.45	0.9813	7.74 ± 0.75		20.26 ± 1.56	0.38
37	46.37	0.9867	146.92	0.9789	45.74 ± 4.30		123.34 ± 15.34	0.37
38	52.79	0.9711	70.44	0.9865	41.16 ± 7.41		59.44 ± 8.01	0.69
39	28.03	0.9869	82.20	0.9876	38.46 ± 8.48		65.24 ± 8.33	0.59
40	52.06	0.9954	101.44	0.9921	34.87 ± 7.28		95.64 ± 20.22	0.36
41	25.33	0.9912	80.13	0.9855	30.69 ± 6.03		75.48 ± 18.45	0.41
42	20.97	0.9861	58.71	0.9889	14.64 ± 3.01		48.56 ± 4.55	0.30
43	29.16	0.9905	130.27	0.9721	19.86 ± 0.73		120.16 ± 25.67	0.17
44	27.57	0.9857	75.44	0.9856	15.68 ± 0.85		61.02 ± 11.13	0.27
45	30.10	0.9915	85.21	0.9766	21.75 ± 1.45		72.45 ± 12.56	0.30
46	31.45	0.9845	84.33	0.9854	29.24 ± 0.64		87.55 ± 13.56	0.33
47	24.03	0.9743	65.21	0.9824	22.28 ± 4.11		46.56 ± 2.10	0.48
48	17.91	0.9826	33.00	0.9957	10.58 ± 0.58		22.67 ± 3.07	0.47
Tacrine	105.21	0.9854	94.13	0.9878	95.21 ± 11.87		88.34 ± 9.12	1.08
Donepezil	69.30	0.9788	63.00	0.9746	59.21 ± 8.21		51.48 ± 9.64	1.15

In compounds **7–12**, the AChE inhibition order according to the groups in the CAS binding region is piperidine (K_i_: 18.65 ± 2.58 nM) > methyl (K_i_: 23.04 ± 1.70 nM) > morpholino (K_i_: 28.56 ± 3.96 nM) > ethyl (K_i_: 30.60 ± 3.34 nM) > pyrrolidine (K_i_: 46.53 ± 6.59) > propyl (K_i_: 59.39 ± 3.69). There was not much difference between the ethyl and morpholino groups in terms of inhibition value (K_i_: 30.60 ± 3.34 nM for compound **8**; K_i_: 28.56 ± 3.96 nM for compound **12**). The piperidine group showed 3.18 times more effective AChE inhibition than the propyl group. Also, when an additional benzene group is attached to the compounds (**7–12**) construct, the inhibition order according to the groups in the basic centers: piperidine (K_i_: 20.21 ± 1.47 nM) > methyl (K_i_: 38.75 ± 2.96 nM) > pyrrolidine (K_i_: 41.91 ± 5.64 nM) > morpholino (K_i_: 45.73 ± 1.65 nM) > ethyl (K_i_: 48.53 ± 9.97 nM) > propyl (K_i_: 71.56 ± 12.01 nM).

The piperidine group showed the best inhibition feature in the binding of biphenyl and naphthalene groups to the aromatic ring portion of compounds **7–12** compounds (K_i_: 18.37 ± 3.33 nM for compound **17**; K_i_: 20.21 ± 1.47 nM for compound **23**). Compared to the others, a lower inhibition value was observed in the compounds to which the propyl group was attached (K_i_: 38.21 ± 0.66 nM for compound **15**; K_i_: 71.56 ± 12.01 nM for compound **21**).

The aromatic ring is pyridine in the structure (compound **25–30**) as shown in Fig. 3, the inhibition order according to the groups in the basic centers: morpholino (K_i_: 18.32 ± 4.37 nM) > piperidine (K_i_: 27.38 ± 2.53 nM) > pyrrolidine (K_i_: 47.92 ± 4.70 nM) > propyl (K_i_: 50.05 ± 5.13 nM) > ethyl (K_i_: 83.13 ± 1.33 nM) > methyl (K_i_: 85.33 ± 11.22 nM). The methyl or ethyl group attached to the CAS binding site did not show much change in inhibition (K_i_: 85.33 ± 11.22 nM for compound **25**; K_i_: 83.13 ± 1.33 nM for compound **26**). Similarly, being bound to pyrrolidine and propyl groups did not cause much change in inhibition (K_i_: 50.05 ± 5.13 nM for compound **27**; K_i_: 47.92 ± 4.70 nM for compound **28**). The morpholino group showed 4.66 times more effective inhibition than the methyl group. When the aromatic ring is the pyrrole ring, inhibition order according to the groups in the basic centers: morpholino (K_i_: 7.74 ± 0.75 nM) > piperidine (K_i_: 12.31 ± 1.28 nM) > pyrrolidine (K_i_: 15.51 ± 1.96 nM) > propyl (K_i_: 26.13 ± 3.35 nM) > methyl (K_i_: 30.70 ± 2.21 nM) > ethyl (K_i_: 47.53 ± 9.80 nM). The morpholino group showed 6.14 times more effective inhibition than the ethyl group. The order of inhibition did not change when the pyridine and pyrrole structure in the aromatic ring shown in Fig. 3 (compounds **37–42** and compound **43–48**) were replaced by the furan and thiophene ring. Again, the morpholino group showed the best inhibitor feature among the group.

**Fig. 4:**
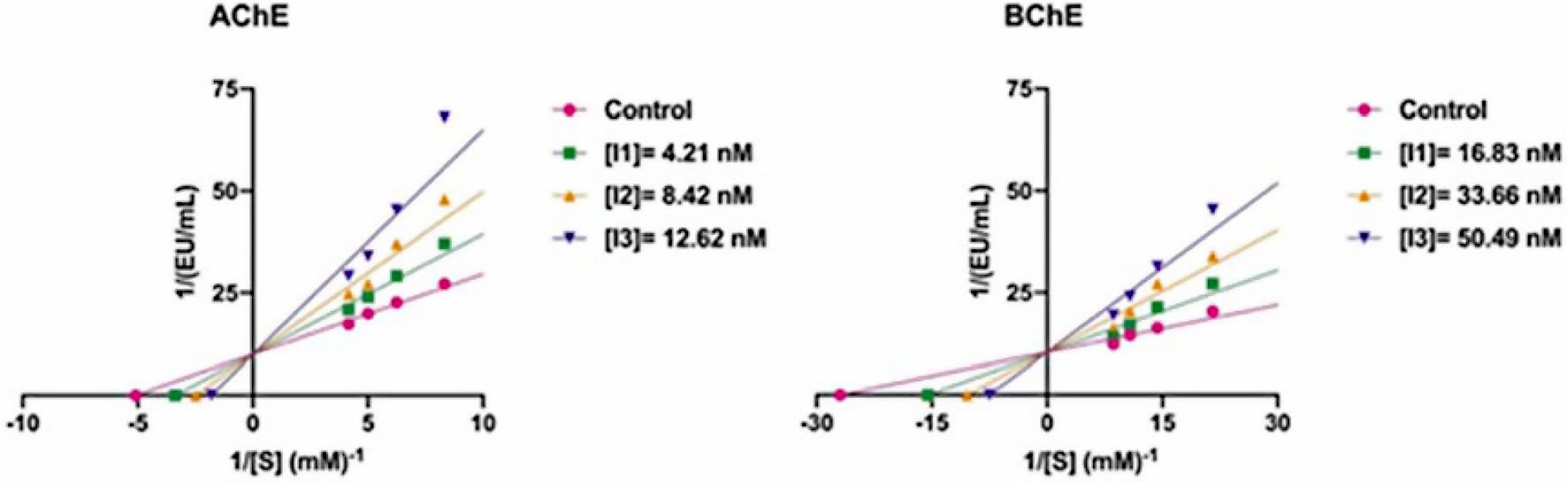
The Lineweaver-Burk graphs of novel 2-aryl-6-carboxamide benzoxazole derivatives (compound **36**) against AChE and BChE.

The novel synthesized 2-aryl-6-carboxamide benzoxazole derivatives (**7–48**) effectively inhibited BChE with IC_50_ values ranging from 25.00 to 188.08 nM and K_i_ values ranging from 20.26 ± 1.56 to 145.13 ± 11.22 nM. As in AChE, compound 2-(*1 H*-pyrrol-2-yl)benzo[d]oxazol-6-yl)(morpholino)methanone (compound **36**) exhibited the best inhibition profile (K_i_: 20.26 ± 1.56 nM) against BChE (Fig. 4). As seen in Table 1, except for 8 compounds (Compounds 9, 15, 19, 20, 21, 37, 40, ve 43), the other compounds demonstrated more effective BChE inhibition effects than TAC (K_i_: 88.34 ± 9.12 nM). The results showed that the compounds of **7–12** exhibited BChE inhibition order according to the groups in the CAS binding region is morpholino (K_i_: 32.33 ± 3.25 nM) > pyrrolidine (K_i_: 36.62 ± 10.97 nM) > methyl (K_i_: 50.74 ± 6.43 nM) > piperidine (K_i_: 74.38 ± 16.89 nM) > ethyl (K_i_: 76.95 ± 5.25 nM) > propyl (K_i_: 111.23 ± 4.67 nM). There was not much difference between the ethyl and piperidine groups in terms of inhibition value (K_i_: 76.95 ± 5.25 nM for compound **8**; K_i_: 74.38 ± 16.89 nM for compound **11**). The morpholino group showed 3.44 times more effective inhibition than the propyl group. When an additional phenyl group is attached to the compound **7–12** constructs, the inhibition order according to the groups in the basic centers: piperidine (K_i_: 49.18 ± 11.13 nM) > morpholino (K_i_: 56.65 ± 1.57 nM) > methyl (K_i_: 65.38 ± 14.05 nM) > ethyl (K_i_: 75.25 ± 13.78 nM) > pyrrolidine (K_i_: 84.03 ± 12.45 nM) > propyl (K_i_: 95.74 ± 7.32 nM). As in AChE, the piperidine aromatic ring showed the most effective inhibition in this group in BChE. Also, when an additional benzene group is linked to the compound **7–12** constructs, inhibition order according to the groups in the basic centers: morpholino (K_i_: 50.69 ± 14.02 nM) > piperidine (K_i_: 69.37 ± 11.34 nM) > pyrrolidine (K_i_: 72.45 ± 5.66 nM) > methyl (K_i_: 121.36 ± 15.45 nM) > ethyl (K_i_: 136.03 ± 30.69 nM) > propyl (K_i_: 145.13 ± 11.22 nM). While the piperidine group was more effective in AChE, the morpholino group was more effective in BChE. The naphthalene group was not effective in BChE inhibition compared to other functional groups.

The aromatic ring is pyridine in the structure (compound **25–30**) shown in Fig. 3, the inhibition order according to the groups in the basic centers: morpholino (K_i_: 45.35 ± 3.04 nM) > piperidine (K_i_: 47.90 ± 8.42 nM) > pyrrolidine (K_i_: 52.92 ± 11.67 nM) > ethyl (K_i_: 54.39 ± 4.85 nM) > methyl (K_i_: 66.40 ± 13.58 nM) > propyl (K_i_: 69.69 ± 4.10 nM). The morpholino or piperidine group attached to the CAS binding site did not show much change in inhibition (K_i_: 47.90 ± 8.42 nM for compound **29**; K_i_: 45.35 ± 3.04 nM for compound **30**). Similarly, being bound to pyrrolidine and ethyl groups did not cause much change in inhibition (K_i_: 54.39 ± 4.85 nM for compound **26**; K_i_: 52.92 ± 11.67 nM for compound **28**). The aromatic ring is pyrrol in the structure the inhibition order according to the groups in the basic centers: morpholino (K_i_: 20.26 ± 1.56 nM) > ethyl (K_i_: 23.01 ± 1.52 nM) > propyl (K_i_: 38.47 ± 8.43 nM) > methyl (K_i_: 41.99 ± 6.32 nM) > piperidine (K_i_: 44.46 ± 7.43 nM) > pyrrolidine (K_i_: 48.22 ± 2.56 nM). The aromatic ring is furan, morpholino (K_i_: 48.56 ± 4.55 nM) > ethyl (K_i_: 59.44 ± 8.01 nM) > propyl (K_i_: 65.24 ± 8.33 nM) > piperidine (K_i_: 75.48 ± 18.45 nM) > pyrrolidine (K_i_: 95.64 ± 20.22 nM) > methyl (K_i_: 123.34 ± 15.34 nM). The aromatic ring is thiophene, morpholino (K_i_: 22.67 ± 3.07 nM) > piperidine (K_i_: 46.56 ± 2.10 nM) > ethyl (K_i_: 61.02 ± 11.13 nM) > propyl (K_i_: 72.45 ± 12.56 nM) > pyrrolidine (K_i_: 87.55 ± 13.56 nM) > methyl (K_i_: 120.16 ± 25.67 nM). As in AChE, while the morpholino group shows the best inhibition, the order of inhibition differs. When the Selectivity index (AChE/BChE) is evaluated, it is seen that the highest Selectivity index (AChE/BChE; 2.07) value was found for the *N,N*-diethyl-2-(1*H*-pyrrol-2-yl)benzo[d]oxazole-6-carboxamide (compound **32**).

In summary, the 42 novel benzoxazole-based derivatives presented here were found to have various inhibitory potentials and selectivity index against AChE and BChE enzymes. Changing the inhibitory effect and selectivity index with small differences in the molecule allows compounds to target the relevant enzyme with a comprehensive structure-activity relationship. It is known that in late-stage Alzheimer’s dementia (AD), the AChE level in the brain gradually decreases, but BuChE activity remains the same or can increase up to 165% of the normal level [46]. Therefore, BuChE is proposed as a proposed drug target for the treatment of late-stage AD, and it has also been reported that inhibitors with more bulky moieties can increase the BChE selectivity index [47]. In this context, compound **32** exhibits approximately 5.5 times higher BChE selectivity compared to compound **36**. This can be explained by the presence of a slightly bulky N-N-diethyl group in compound **32** compared to the morpholine group found in compound **36**. Lastly, the binding affinities of the compounds towards the active sites of AChE and BChe enzymes were investigated by molecular docking and dynamic simulations.

### Molecular docking and molecular dynamics simulations

Molecular modeling and molecular dynamics simulations are critical in the development of novel and efficient cholinesterase inhibitors. The active areas of cholinesterase enzymes contain critical amino acids that could be used in the development of effective drugs. Tyr70, Asp72, Tyr121, Trp279, and Tyr334 are found in the active site of AChE, which is 20 Å deep and 5 wide. Furthermore, the active site of the enzyme, CAS, is deeper. CAS is made up of the catalytic triad His440, Glu327, and Ser200, as well as the essential amino acid Trp84. BChE’s active site is also about 20 deep and slightly wider than AChE’s. The active site contains the catalytic triad of amino acids Ser198, His438, and Glu325. Glu197, Trp231, Phe329 (acyl-binding pocket), Asp70, and Tyr332 are also found in the active site (PAS) [48]. Compounds must interact with these residues to inhibit choline esterase, and this interaction must last for a certain amount of time [49, 50].

### Compound 36 in complex with AChE

In molecular modeling studies, it was aimed determine the binding modes of compounds to the active sites of AChE. Modeling studies were performed with compounds **7–48**, tacrine, and donepezil (co-crystallized ligand in 1EVE). In modeling studies, the proposed binding modes of ligands were inserted into the active gorge of AChE.

The docking scores of the synthesized compounds were calculated as -6.075 and − 7.291 kcal/mol (Table 2). The docking scores of the compounds are proportional to the order of the experimental IC_50_ values. Compound **36** with the highest docking score (-7.291 kcal/mol) has the best IC_50_ (12.62 nM) value, which also supports this situation.

**Table 2:** Docking scores of synthesized compounds **7–48**, tacrine, and donepezil against 1EVE and 4BDS

Compounds	1EVE	4BDS	Compounds	1EVE	4BDS
7	-6.659	-4.749	29	-6.769	-5.227
8	-6.587	-4.215	30	-6.795	-5.427
9	-6.274	-3.773	31	-6.595	-5.450
10	-6.489	-5.331	32	-6.452	-6.360
11	-6.838	-5.041	33	-6.452	-5.275
12	-6.794	-6.080	34	-6.806	-5.295
13	-6.618	-4.808	35	-6.987	-5.412
14	-6.651	-3.941	36	-7.291	-6.719
15	-6.496	-3.841	37	-6.264	-3.598
16	-6.791	-4.057	38	-6.216	-4.492
17	-6.977	-5.138	39	-6.763	-4.067
18	-6.933	-4.746	40	-6.202	-3.899
19	-6.587	-3.600	41	-6.854	-4.201
20	-6.243	-3.095	42	-6.995	-5.167
21	-6.075	-2.974	43	-6.663	-3.609
22	-6.416	-3.704	44	-6.893	-4.223
23	-6.785	-3.978	45	-6.719	-3.975
24	-6.302	-4.009	46	-6.623	-3.999
25	-6.230	-3.817	47	-6.899	-4.667
26	-6.429	-4.211	48	-7.104	-6.202
27	-6.354	-3.996	Donepezil	-6.492	-5.057
28	-6.414	-5.168	TAC	-5.150	-3.822

**Fig. 5:**
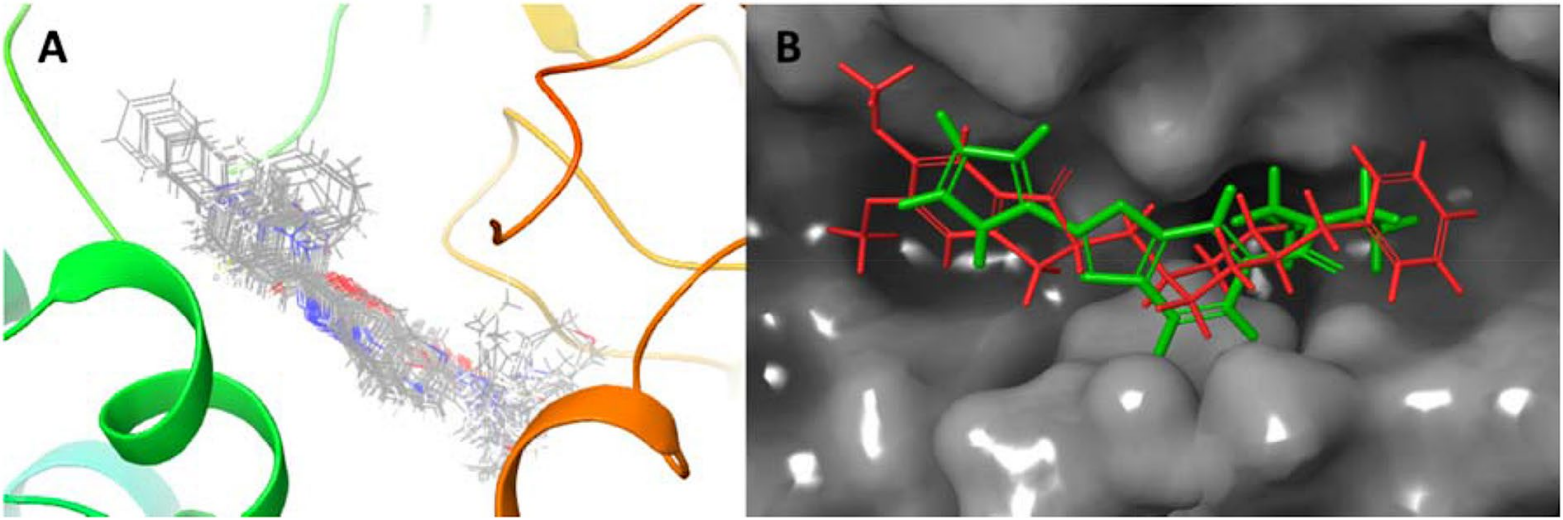
Superimposition of the top docking poses of the compounds **7**–**48** in AChE (**A**). Similar binding modes of donepezil (red) and compound **36** (green) in AChE (**B**)

Overall, we found that the binding modes for all compounds (**7–48**) were similar pose present in AChE (Fig. 5A). In addition, when donepezil and compound **36** were superimposed on the active site of the protein, it was observed that their exposures were close (Fig. 5B). The docking scores of the most active compound 36 (IC50: 12.62 nM) and reference compound donepezil (IC_50_: 69.30 nM) against AChE were calculated as -7.291 and − 6.492 kcal/mol, respectively. As a result of modeling studies of compound **36** against AChE, its interaction with the active site of the protein was investigated.

**Fig. 6:**
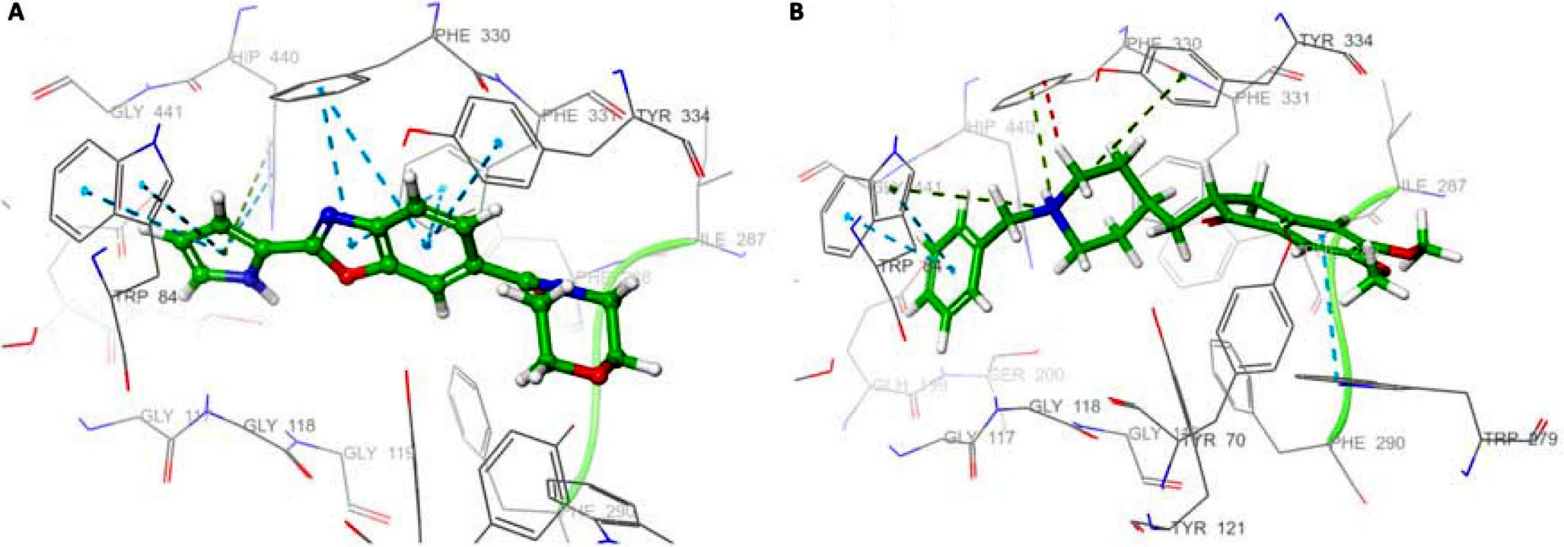
3D mode of interactions of compound **36** (**A**) and donepezil (**B**) with receptor AChE. C atoms are presented in green; N atoms are presented in blue and O atoms are presented in red respectively; π-cation interactions are shown in dark green; π-π interactions are shown in light blue

Compound **36** formed π-π and π-cation interactions with His440, located in the CAS region of the protein, and π-π interaction with Trp84, with the pyrrole ring in the second position. Compound **36** has formed π-π interactions with Phe330 and Tyr334 via the benzoxazole ring and also has hydrophobic interactions with Tyr70, Trp279, and Tyr121 located in the PAS region of the protein (Fig. 6A). The results show that the compound interacts with residues in both PAS and CAS. In the docking study with donepezil, donepezil formed π-cation interactions with Trp84, Phe330, and Tyr334 and formed π-π interactions with Trp84 and Trp279. It can be said that compound **36** has interactions like donepezil in the active gorge of AChE (Fig. 6B). Unlike donepezil, the interaction of compound **36** with one of the important key residues, Trp84, is thought to cause both the IC_50_ and docking score of the compound to be higher than donepezil.

**Fig. 7:**
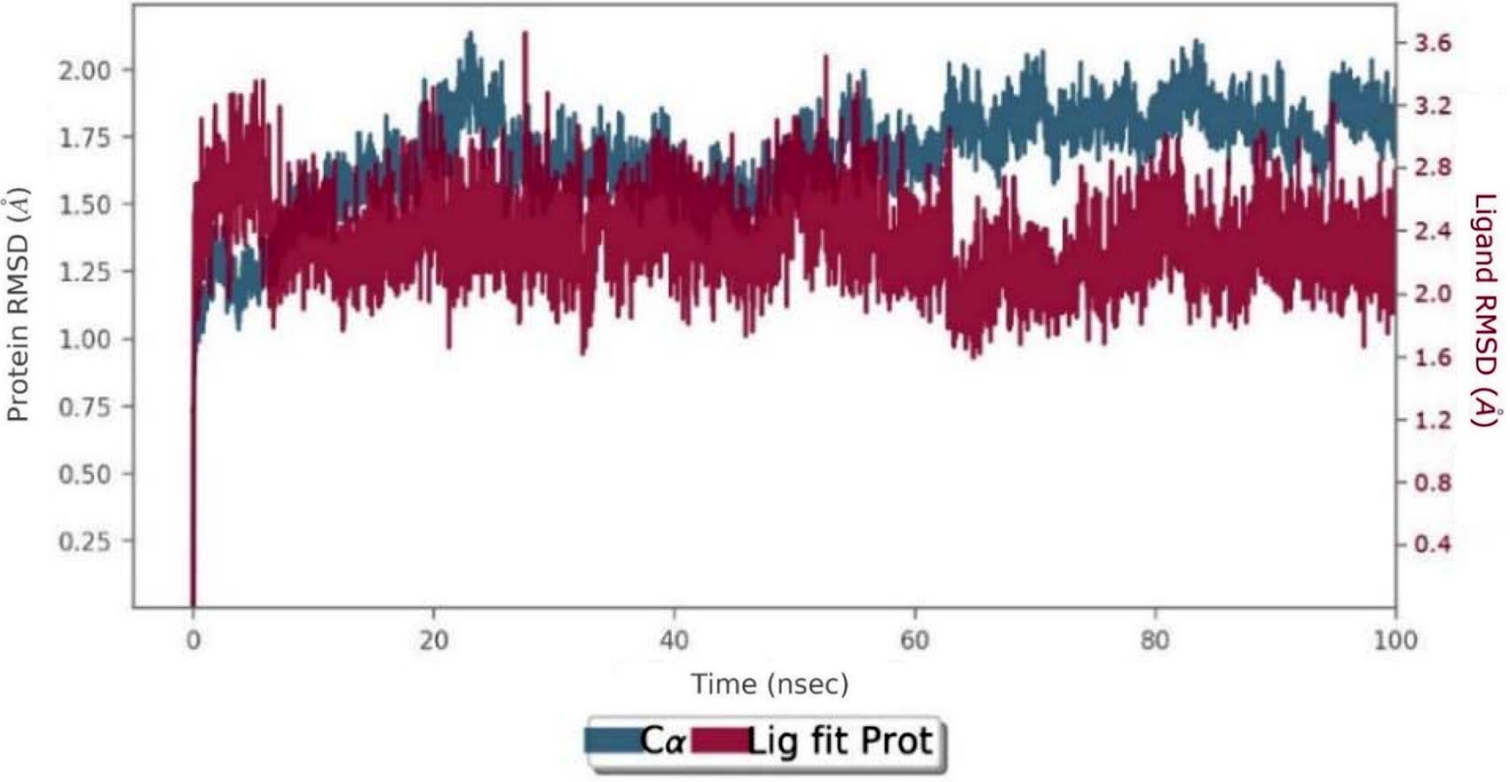
RMSD plot of 100ns molecular dynamic simulations for compound **36** inside AChE. Blue: Cα (RMSD evolution of the protein). Pink: Lig fit Prot (RMSD of the ligand when the protein-ligand complex)

Molecular dynamics simulations were performed for 100 ns with the selected pose of Compound **36** in the AChE complex. Throughout the simulation, the average RMSD values of compound **36** and protein were calculated as 1.98 Å and 1.28 Å, respectively (Fig. 7). The RMSD plot shows that compound **36** localizes to the active site of the protein and remains stable. The results of this simulation indicate that compound **36** binds strongly to the active site of AChE, suggesting that it possesses the lowest IC_50_.

When the interaction of compound **36** with the protein was examined by the simulation, it was observed that it had significant hydrophobic interactions, especially with Tyr70, Trp84, Phe331, Phe330, Tyr334, Trp279, Tyr121, and His440 (Fig. S127). When the time graph of the interaction is examined (the timeline representation of the interactions and contacts), it can be said that compound **36** interacts with Trp84, Phe331, and Tyr334 roughly throughout the simulation (Fig. S128).

### Compound 36 in complex with BChE

**Fig. 8:**
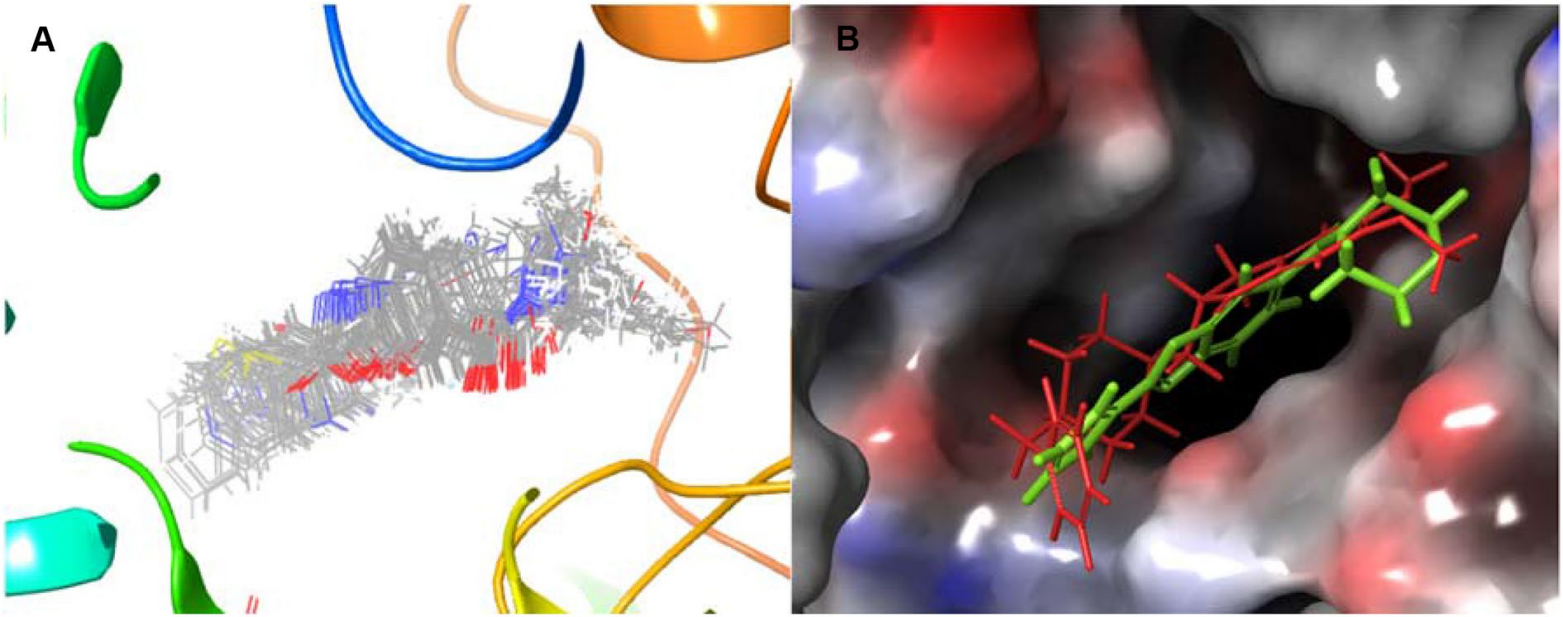
Superimposition of the top docking poses of the compounds **7**–**48** in BChE (**A**). Similar binding modes of compound **36** (green) and donepezil (red) in BChE (**B**)

The synthesized compounds **7–48**, tacrine, and donepezil were docked at the active site of BChE, and binding poses and interactions of the ligands were investigated. The binding poses of compounds **7–48** were found to be similar. Donepezil and compound **36** were superimposed on the active site of the protein, and their binding poses were observed (Fig. 8). As a result of molecular modeling studies, the docking scores of the compounds (**7–48**) were calculated between − 2.549 and − 6.719 kcal/mol (Table 2).

**Fig. 9:**
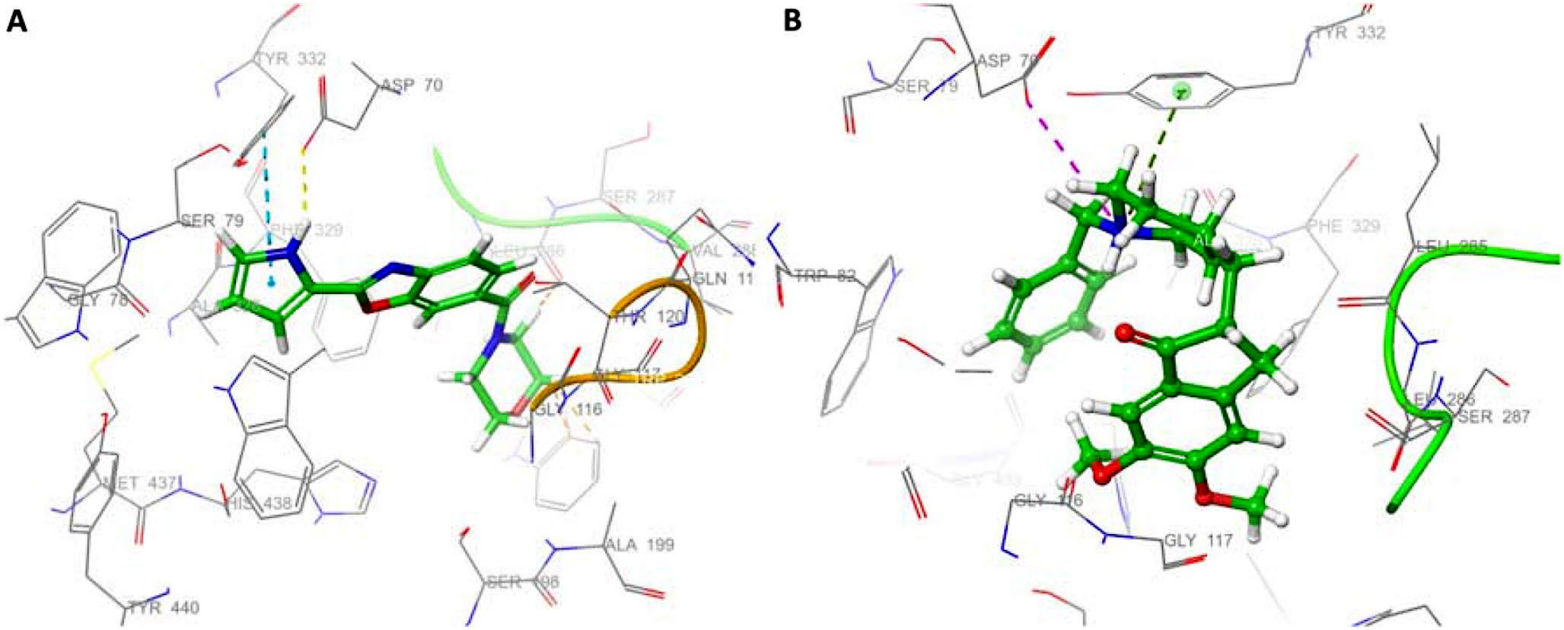
3D mode of interactions of compound **36** (**A**) and donepezil (**B**) with receptor BChE. C atoms are presented in green; N atoms are presented in blue and O atoms are presented in red respectively; hydrogen bonds are shown in yellow, π-cation interactions are shown in dark green; π-π interactions are shown in light blue

Regarding docking studies, the docking scores of the most active compound **36** (IC_50_: 25.45 nM) and reference compound donepezil (IC_50_: 63.00 nM) against BChE were calculated as -6.719 and − 5.818 kcal/mol, respectively. Compound **36** formed hydrogen bond with Asp70 (distance: 2.12 Å) and formed π-π interactions with Tyr332 (PAS) in the active site of BChE via the pyrrole ring. In addition, it interacted hydrophobicically with Phe329 (acyl-binding site) and Trp231 (Fig. 9). When the interaction of donepezil with the active site of BChE is examined, donepezil formed pi-cation with Tyr332 via the piperidine ring and formed a salt bridge with Asp70. Donepezil also had hydrophobic interactions with Phe329 and Trp231, which are important residues of the binding site. Compound **36** and donepezil were found to have similar interactions at the active site of BChE. It can be said that the hydrogen bonding of compound **36** with Asp70 has a significant effect on binding to the active site of the protein more strongly than donepezil.

**Fig. 10:**
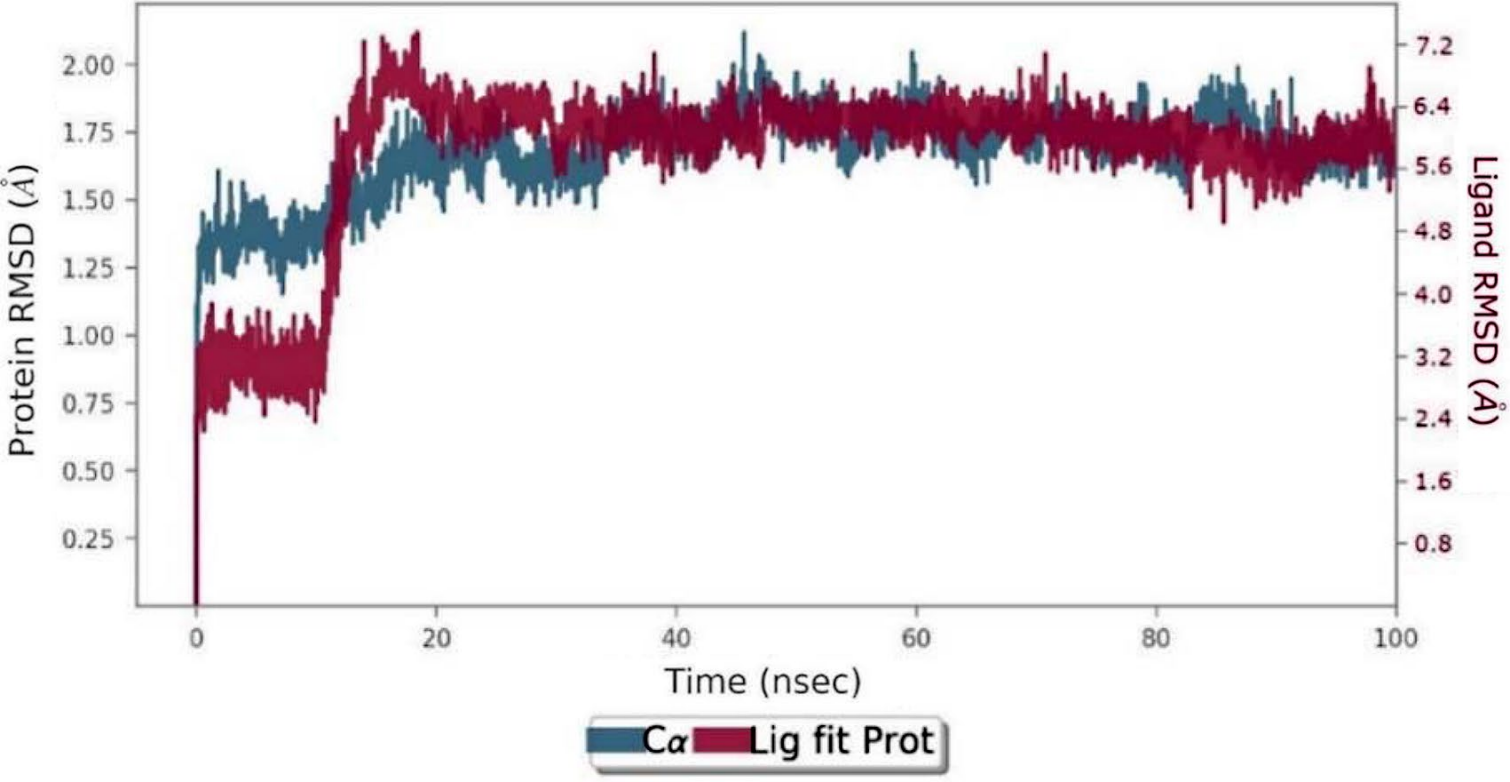
RMSD plot of 100ns molecular dynamic simulations for **36** inside BChE. Blue: Cα (RMSD evolution of the protein). Pink: Lig fit Prot (RMSD of the ligand when the protein-ligand complex)

Molecular dynamic simulations were carried out for 100ns with the selected pose of compound **36** with the best docking score. The RMSD value of the ligand until the 15th ns is between 2.4 Å and 3.2 Å, while the RMSD value of the ligand from the 20th ns until the end of the stimulation is between 4.8 Å and 7.0 Å (Fig. 10).

It is thought that this change in RMSD values is caused by compound **36** interacting with Glu197 at the beginning of the simulation, closing to Asp70, and beginning to interact with Asp70 due to the change in its conformation from about 15 ns (Fig. S129). It can be said that after the 15th ns, the RMSD value for ligand reached roughly the plateau value. During the simulation, the average RMSD value of the protein is 0.4 Å. During the simulation, compound **36** interacted with Asp70, Trp82, Trp231, Phe239, Leu285, and Tyr332 (Fig. S130).

In summary, the 42 synthesized compounds demonstrate a targeted interaction with the active sites of both AChE and BChE enzymes, mimicking the binding position of donepezil and focusing on the CAS and PAS domains of both enzymes. Notably, compound **36**, identified as the most potent inhibitor in in vitro enzyme inhibition studies, exhibited a superior binding affinity for both enzymes compared to donepezil and other compounds, as indicated by docking scores.

The heightened inhibition potential of compound **36**, surpassing that of donepezil, can be attributed to the pyrrole structure establishing a π-π interaction with Trp84, a crucial residue in the AChE active site. This enhanced interaction is further supported by the extended engagement of compound **36** with Trp84 observed in molecular dynamics simulations. Additionally, the pyrrole N-H group in compound **36** displays a significantly stronger binding affinity than donepezil, forming a hydrogen-bonding interaction with Asp70 in the BChE active site. Molecular dynamics studies further illustrate a long-range interaction between compound **36** and Asp70, emphasizing the importance of this residue in the compound’s binding mechanism.

### ADME study

Prediction of the blood-brain barrier (BBB) is useful for drugs targeting the central nervous system (CNS) to have a notion of a compound’s ability to permeate this barrier. Therefore, QPlogBB was used to describe the properties of cholinesterase inhibitors. ADME studies also include estimated central nervous system (CNS) activities, blood/brain partition coefficients, oral percent absorption, cell permeability (MDCK cell permeability), and BBB permeation (Table S1). The majority of the estimated parameters are within acceptable ranges according to the reported guidelines.

All of the compounds (except for compound **30**) have the potential to be active in the CNS. The QlogBB coefficients of the compounds range from − 0.568 to 0.116, with these coefficients in the recommended range (–3.0–1.2). The predicted apparent MDCK cell permeability (QPPMDCK) values of all compounds are within acceptable limits. All but compounds **15**, **21**, **45**, and **48** were found to be BBB permeable. Also, compounds generally obey Lipinski’s rule of five. The percent absorption values of its compounds are between 92.55 and 100. These findings inform us that the compounds may be promising cholinesterase inhibitor candidates.

## Conclusion

In conclusion, we have successfully introduced, for the first time, a new class of compounds — 2-aryl-6-carboxamide benzoxazole derivatives — that exhibit inhibitory effects on cholinesterases. Enzyme assays and kinetics studies were employed to evaluate the inhibitory effects of 42 newly synthesized compounds on both AChE and BChE.

This study holds the potential to provide preliminary data for the development of a design strategy in medicinal chemistry research, contributing to the chemical library for compounds targeting the CAS and PAS binding sites of cholinesterases.

Promisingly, all designed compounds demonstrated higher inhibitory effects on AChE (IC_50_: 12.62 nM − 67.02 nM) compared to donepezil and tacrine (IC_50_: 69.30 nM, 105.21). Specifically, compounds **10–12, 17, 28–36, 42**, and **48** (IC_50_: 25.45 nM − 61.25 nM) exhibited greater effectiveness against BChE than donepezil and tacrine (IC_50_: 63.00 nM, 94.13 nM). The most potent compound among the designed molecules, compound 36, demonstrated inhibition concentrations of 12.62 nM for AChE and 25.45 nM for BChE. In molecular docking studies, compound **36** exhibited higher binding affinity for both AChE (-7.29 kcal/mol) and BChE (-6.71 kcal/mol) compared to donepezil (-6.49 kcal/mol and − 5.057 kcal/mol, respectively). Molecular docking studies revealed that compound 36 binds to both the CAS and PAS domains of proteins, with the pyrrole ring in its structure binding to the CAS region and the benzoxazole ring playing a crucial role in binding to the PAS region.

Molecular dynamics (MD) simulations were conducted to assess the stability of compound **36** in the active sites of target proteins. The results indicated that compound **36** remained stable in the active gorges of both AChE (average RMSD: 1.98 Å) and BChE (average RMSD: 2.2 Å). Furthermore, the presented compounds exhibited suitable ADME properties and druggability potential.

Cholinesterase (ChE) inhibitors, frequently employed in AD treatment, target AChE and BChE. The compounds designed in our study demonstrated significant inhibition effects on cholinesterases, suggesting potential use in the treatment of AD, a prevalent and global disease. However, the effects of these compounds on AD require confirmation through further experimental verification.

## Materials and methods

Commercially available materials were used without further purification. 4-Amino-3-hydroxy-benzoic acid (Acros), benzaldehyde (Merck), biphenyl-4-carboxaldehyde (Sigma-Aldrich) α-naphthaldehyde (Merck), 4-pyridinecarboxaldehyde (Merck), 2-pyrrolecarboxaldehyde (Saffchemical) Furfural (Sigma-Aldrich), 2-thiophenecarboxaldehyde (Sigma-Aldrich), dimethylamine hydrochloride (Acros), diethylamine hydrochloride (Acros), diisopropylamine (Acros), pyrrolidine (Acros) and piperidine (Acros) and morpholine (Acros) were used as synthesis starting materials. As solvent, catalyst and co-reactant; methanol (Isolab), ethanol (Isolab), sulfuric acid (H_2_SO_4_; 98%, Isolab), dichloromethane (DCM, Sigma-Aldrich), *N’N’*-dimethylformamide (DMF, Merck), sodium cyanide (NaCN, Sigma-Aldrich), aluminum trichloride (AlCl_3_, Merck), n-hexane (Abcr), ethylacetate (Abcr), magnesium sulfate (MgSO_4_, Isolab), sodium bicarbonate (NaHCO_3_, Sigma-Aldrich) were used. In addition, Kieselgel 60 F_254_ 2 mm thick coated ready-made aluminum plates (Silicycle) were used in thin layer chromatography (TLC) studies. ^1^H-NMR, and ^13^C-NMR, spectra were recorded at 400, and 100 MHz, respectively, on a Varian-Agilent 400 MHz instrument using Me_4_Si as an internal standard. All column chromatography was performed on silica gel (60 mesh, Silycycle). New compounds for HRMS were tested on a Thermo Scientific Q Exactive MS/MS system ESI spectrometer. Chemical shift multiplicities are represented as follows: (s = singlet, d = doublet, t = triple, dd = double doublet, and m = multiplet).

## Chemistry

### Synthesis of methyl 4-amino-3-hydroxybenzoate

The 4-amino-3-hydroxy-benzoic acid (5 mmol) was dissolved in 10 mL of methanol and refluxed for 12 h under the catalyst of a few drops (5% mol) of concentrated H_2_SO_4_. After the completion of the reaction was checked with TLC, the reaction was finished and cooled to room temperature. It was neutralized with 50 mL of water and 1 N NaHCO_3_ until pH:7.5. The resulting aqueous phase was extracted with 3 × 15 mL of ethyl acetate. The organic phase was separated, the solvent was evaporated and the crude product was purified by column chromatography in the appropriate n-hexane/ethylacetate (5/1) mobile phase (Scheme 1) [51].

### General procedure for the synthesis 2-aryl benzoxazole derivatives

A mixture of methyl 4-amino-3-hydroxybenzoate (1 mmol) was dissolved in 5 mL ethanol and the corresponding aromatic carboxaldehyde derivatives (1.1 mmol) were added to the reaction mixture. The reaction mixture was stirred for a period of 6–24 h at room temperature and the reaction completion was controlled with the TLC method. After observing that the starting materials were disappeared, the reaction mixture was poured into ice water to get the precipitate. The resulting precipitate was filtered off and washed with cold water. The solid products were used in the second step without isolation. Solid products (1 mmol) were dissolved in 5 mL of DMF and a catalytic amount of NaCN was added. The reaction mixture was stirred at room temperature for 1 h. With the starting material disappearing, the reaction mixture was transferred to the ice-water mixture to get the precipitate. The resulting precipitate was filtered off and washed with brine. The final products were purified with column chromatography 5:1 (n-hexane: ethylacetate) (Scheme 1) [52].

### General procedure for the synthesis 2-aryl-6-carboxamide benzoxazole derivatives

2-Aryl benzoxazole derivatives (1 mmol) synthesized in the previous step were dissolved in 4 mL of dichloromethane (DCM) and 1.2 mmol of cyclic or linear aliphatic secondary amine (pyrrolidine, piperidine, morpholine or dimethylamine, diethylamine, dipropylamine) derivatives were added. After the reaction mixture was stirred at room temperature for 10 min, a catalytic amount of aluminum (III) chloride (AlCl_3_) was added. The reaction mixture was stirred at room temperature for 24 h. The completion of the reaction was checked with TLC and the reaction was finished by the addition of water. The resulting mixture was extracted by adding 3 × 15 mL of dichloromethane (DCM) and 50 mL of water. The organic phases were collected, dried with MgSO_4_ and filtered. The crude product obtained by evaporation of the organic solvent was washed successively with diethyl ether and cyclohexane.

In fact, the reaction conditions were first adjusted in dichloromethane (DCM) at room temperature by addition of straight chain or cyclic secondary amines [53]. However, the reaction did not proceed at this temperature and the situation did not change with the increase in temperature. Therefore, in order to increase the reactivity of the ester carbonyl, AlCl_3_ was added to the reaction mixture as a catalytic amount of Lewis acid. The mechanism of the mentioned reaction is presented in Scheme 1.

### ***N,N*** -dimethyl-2-phenylbenzo[***d***]oxazole-6-carboxamide (7)

White solid, m.p. 111–112 °C, R_*f*_; 0.65 in ethylacetate/n-hexane (3/1), Yield; 74%. ^1^H-NMR (400 MHz, CDCl_3_) ppm δ = 8.22–8.18 (m, 2 H, Ar-H), 7.72 (dd, *J* = 0.6 Hz, *J* = 8.2 Hz, 1H, Ar-H), 7.64 (dd, *J* = 0.6 Hz, *J* = 1.5 Hz, 1H, Ar-H), 7.51–7.46 (m, 3 H, Ar-H), 7.39 (dd, *J* = 1.5 Hz, *J* = 8.2 Hz, 1H, Ar-H), 3.09 (bs, 3 H, -CH_3_), 2.98 (bs, 3 H, -CH_3_). ^13^C-NMR (100 MHz, CDCl_3_) ppm δ = 170.8, 164.3, 150.3, 143.1, 133.2, 131.9, 128.9, 127.7, 126.7, 123.9, 119.7, 110.0, 39.7, 35.5. HRMS [M + H] (C_16_H_15_N_2_O_2_): Calculated: 267.1134; Found: 267.1167.

### ***N,N***-diethyl-2-phenylbenzo[***d***]oxazole-6-carboxamide (8)

Gray solid, m.p. 129–130 °C, R_*f*_; 0.74 in ethylacetate/n-hexane (3/1), Yield; 71%. ^1^H-NMR (400 MHz, CDCl_3_) ppm δ = 8.25–8.21 (m, 2 H, Ar-H), 7.75 (dd, *J* = 0.5 Hz, *J* = 8.1 Hz, 1H, Ar-H), 7.61 (dd, *J* = 0.5 Hz, *J* = 1.5 Hz, 1H, Ar-H), 7.54–7.48 (m, 3 H, Ar-H), 7.36 (dd, *J* = 1.5 Hz, *J* = 8.1 Hz, 1H, Ar-H), 3.54 (bs, 2 H, -CH_2_-), 3.29 (bs, 2 H, -CH_2_-), 1.33–1.05 (m, 6 H, -CH_3_(2x)). ^13^C-NMR (100 MHz, CDCl_3_) ppm δ = 170.5, 164.2, 150.4, 142.8, 134.2, 131.8, 128.9, 127.7, 126.8, 123.2, 119.9, 109.1, 43.4, 39.5, 14.1, 13.0. HRMS [M + H] (C_18_H_19_N_2_O_2_): Calculated: 295.1447; Found: 295.1153.

### 2-Phenyl-***N,N***dipropylbenzo[d]oxazole-6-carboxamide (9

Orange viscous liquid, R_*f*_; 0.65 in ethylacetate/n-hexane (2/1), Yield; 95%. ^1^H-NMR (400 MHz, CDCl_3_) ppm δ = 8.27–8.23 (m, 2 H, Ar-H), 7.76 (dd, *J* = 0.60 Hz, *J* = 8.13 Hz, 1H, Ar-H), 7.61 (dd, *J* = 0.60 Hz, *J* = 1.47 Hz, 1H, Ar-H), 7.55–7.50 (m, 3 H, Ar-H), 7.35 (dd, *J* = 1.47 Hz, *J* = 8.13 Hz, 1H, Ar-H), 3.48 (bs, 2 H, -CH_2_-), 3.21 (bs, 2 H, -CH_2_-), 1.71 (bs, 2 H, -CH_2_-), 1.56 (bs, 3 H, -CH_2_-), 0.99 (bs, 2 H, -CH_3_), 0.74 (bs, 3 H, -CH_3_). ^13^C-NMR (100 MHz, CDCl_3_) ppm δ = 171.0, 164.2, 150.4, 142.7, 134.4, 131.8, 129.0, 127.7, 126.8, 123.3, 119.9, 109.3, 50.9, 46.5, 21.9, 20.7, 11.4, 11.1. HRMS [M + H] (C_20_H_23_N_2_O_2_): Calculated: 323.1760; Found: 323.1784.

### (2-Phenylbenzo[***d*** oxazol-6-yl)(pyrrolidin-1-yl)methanone (10)

White solid, m.p. 144–145 °C, R_*f*_; 0.68 in ethylacetate/n-hexane (1/1), Yield; 80%. ^1^H-NMR (400 MHz, CDCl_3_) ppm δ = 8.29–8.25 (m, 2 H, Ar- H), 7.80–7.75 (m, 2 H, Ar-H), 7.58–7.51 (m, 4 H, Ar-H), 3.69 (t, *J* = 7.0 Hz, 2 H, -CH_2_-), 3.50 (t, *J* = 6.6 Hz, 2 H, 2 H, -CH_2_-), 2.03–1.96 (m, 2 H, 2 H, -CH_2_-), 1.93–1.87 (m, 2 H, 2 H, -CH_2_-). ^13^C-NMR (100 MHz, CDCl_3_) ppm δ = 169.0, 164.5, 150.3, 143.3, 134.2, 131.9, 129.0, 127.8, 126.8, 124.0, 119.6, 110.0, 49.9, 46.4, 26.5, 24.5. HRMS [M + H] (C_18_H_17_N_2_O_2_): Calculated: 293.1290; Found: 293.1324.

### (2-Phenylbenzo[***d*** oxazol-6-yl)(piperidin-1-yl)methanone (11)

Light orange solid, m.p. 127–128 °C, R_*f*_; 0.80 in ethylacetate/n-hexane (2/1), Yield; 65%. ^1^H-NMR (400 MHz, CDCl_3_) ppm δ = 8.29–8.24 (m, 2 H, Ar-H), 7.77 (dd, *J* = 0.6 Hz, *J* = 8.2 Hz, 1H, Ar-H), 7.66 (dd, *J* = 0.6 Hz, *J* = 1.5 Hz, 1H, Ar-H), 7.57–7.53 (m, 3 H, Ar-H), 7.40 (dd, *J* = 1.5 Hz, *J* = 8.2 Hz, 1H, Ar-H), 3.74 (bs, 2 H, -CH_2_-), 3.40 (bs, 2 H, -CH_2_-), 1.70 (bs, 4 H, -CH_2_-), 1.56 (bs, 2 H, -CH_2_-). ^13^C-NMR (100 MHz, CDCl_3_) ppm δ = 169.7, 164.3, 150.4, 143.0, 133.4, 131.9, 129.0, 127.8, 126.8, 123.7, 119.9, 109.7, 48.9, 43.4, 26.7, 25.8, 24.6. HRMS [M + H] (C_19_H_19_N_2_O_2_): Calculated: 307.1447; Found: 307.1480.

### Morpholino(2-phenylbenzo[***d*** oxazol-6-yl)methanone (12)

Light brown solid, m.p. 117–118 °C, R_*f*_; 0.70 in ethylacetate/n-hexane (2/1), Yield; 74%. ^1^H-NMR (400 MHz, CDCl_3_) ppm δ = 8.28–2.24 (m, 2 H, Ar-H), 7.79 (dd, *J* = 0.6 Hz, *J* = 8.1 Hz, 1H, Ar-H), 7.68 (dd, *J* = 0.6 Hz, *J* = 1.5 Hz, 1H, Ar-H), 7.57–7.52 (m, 3 H, Ar-H), 7.41 (dd, *J* = 1.5 Hz, *J* = 8.1 Hz, 1H, Ar-H), 3.86–3.50 (m, 8 H). ^13^C-NMR (100 MHz, CDCl_3_) ppm δ = 169.7, 169.8, 164.6, 150.5, 143.5, 132.1, 132.0, 129.0, 127.8, 126.7, 123.9, 120.0, 110.1, 66.9. HRMS [M + H] (C_18_H_17_N_2_O_3_): Calculated: 309.1239; Found: 309.1253.

### 2-([1,1’-Biphenyl]-4-yl)-***N,N***-dimethylbenzo[***d***]oxazole-6-carboxamide (13)

Dark orange solid, m.p. 185–186°C, R_*f*_; 0.84 in ethylacetate/n-hexane (3/1), Yield; 70%. ^1^H-NMR (400 MHz, CDCl_3_) ppm δ = 8.36–8.31 (m, AA’BB’ system, 2 H, Ar-H), 7.81–7.76 (m, 3 H, Ar-H), 7.76–7.70 (m, 3 H, Ar-H), 7.52–7.47 (m, 2 H, Ar-H), 7.44 (dd, *J* = 1.5 Hz, *J* = 8.2 Hz, 1H, Ar-H), 7.43–7.38 (m, 1H, Ar-H), 3.16 (bs, 3 H, -CH_3_), 3.06 (bs, 3 H, -CH_3_). ^13^C-NMR (100 MHz, CDCl_3_) ppm δ = 170, 9, 164.3, 150.4, 144.6, 143.2, 139.9, 133.2, 129.0, 128.3, 128.2, 127.6, 127.2, 125.5, 124.0, 119.7, 110.0, 39.8, 35.6. HRMS [M + H] (C_22_H_19_N_2_O_2_): Calculated: 343.1447; Found: 343.1481.

### 2-([1,1’-Biphenyl]-4-yl)-***N,N***-diethylbenzo[***d***oxazole-6-carboxamide (14)

Dark orange viscous liquid, R_*f*_; 0.70 in ethylacetate/n-hexane (3/1), Yield; 66%. ^1^H-NMR (400 MHz, CDCl_3_) ppm δ = 8.37–8.29 (m, AA’BB’ system, 2 H, Ar-H), 7.80–7.76 (m, 3 H, Ar-H), 7.70–7.64 (m, 3 H, Ar-H), 7.52–7.46 (m, 2 H, Ar-H), 7.43–7.37 (m, 2 H, Ar-H),3.58 (bs, 2 H, -CH_2_-), 3.33 (bs, 2 H, -CH_2_-), 1.28–1.14 (m, 6 H, -CH_3_(2x)). ^13^C-NMR (100 MHz, CDCl_3_) ppm δ = 170.6, 164.1, 150.4, 144.6, 142.9, 139.9, 134.2, 129.0, 128.2, 128.1, 127.6, 127.2, 125.6, 123.2, 119.9, 109.2, 43.6, 39.5, 14.1, 13.0. HRMS [M + H] (C_24_H_23_N_2_O_2_): Calculated: 371.1760; Found: 371.1781.

### 2-([1,1’-Biphenyl]-4-yl)-***N,N***-dipropylbenzo[***d***]oxazole-6-carboxamide (15)

Light yellow solid, m.p. 87–88°C, R_*f*_; 0.85 in ethylacetate/n-hexane (1/1), Yield; 85%. ^1^H-NMR (400 MHz, CDCl_3_) ppm δ = 8.35–8.32 (m, AA’BB’ system, 2 H, Ar-H), 7.80–7.76 (m, 3 H, Ar-H), 7.70–7.65 (m, 2 H, Ar-H), 7.63 (dd, *J* = 0.6 Hz, *J* = 1.5 Hz, 1H, Ar-H), 7.52–7.46 (m, 2 H, Ar-H), 7.44–7.39 (m, 1H, Ar-H), 7.37 (dd, *J* = 1.5 Hz, *J* = 8.2 Hz, 1H, Ar-H), 3.50 (bs, 2 H, -CH_2_-), 3.23 (bs, 2 H, -CH_2_-), 1.7. (bs, 2 H, -CH_2_-), 1.56 (bs, 2 H, -CH_2_-), 1.01 (bs, 3 H, -CH_3_), 0.76 (bs, 2 H, -CH_3_). ^13^C-NMR (100 MHz, CDCl_3_) ppm δ = 171.0, 164.1, 150.4, 144.6, 142.8, 139.9, 134.3, 129.0, 128.2, 128.1, 127.6, 127.2, 125.6, 123.4, 119.9, 109.3, 50.9, 46.5, 20.7, 20.3, 11.5, 11.1. HRMS [M + H] (C_26_H_27_N_2_O_2_): Calculated: 399.2073; Found: 399.2084.

### (2-([1,1’-Biphenyl]-4-yl)benzo[***d***]oxazol-6-yl)(pyrrolidin-1-yl)methanone (16)

White solid, m.p. 159–160°C, R_*f*_; 0.86 in ethylacetate/n-hexane (3/1), Yield; 61%. ^1^H-NMR (400 MHz, CDCl_3_) ppm δ = 8.37–8.31 (m, AA’BB’ system, 2 H, Ar-H), 7.82–7.74 (m, 4 H, Ar-H), 7.70–7.66 (m, 2 H, Ar-H), 7.56 (dd, *J* = 1.5 Hz, *J* = 8.2 Hz, 1H, Ar-H), 7.52–7.47 (m, 2 H, Ar-H), 7.44–7.38 (m, 1H, Ar-H), 3.70 (t, *J* = 6.9 Hz, 2 H, -CH_2_-), 3.51 (t, *J* = 6.6 Hz, 2 H, -CH_2_-), 2.03–1.97 (m, 2 H, -CH_2_-), 1.94–1.88 (m, 2 H, -CH_2_). ^13^C-NMR (100 MHz, CDCl_3_) ppm δ = 169.0, 164.4, 150.3, 144.6, 143.4, 139.9, 134.2, 129.0, 128.3, 128.2, 127.6, 127.2, 125.5, 124.1, 119.6, 110.0, 49.9, 46.4, 26.5, 24.5. HRMS [M + H] (C_24_H_21_N_2_O_2_): Calculated: 369.1603; Found: 369.1633.

### (2-([1,1’-Biphenyl]-4-yl)benzo[***d***]oxazol-6-yl)(piperidin-1-yl)methanone (17)

White solid, m.p. 165–166°C, R_*f*_; 0.82 in ethylacetate/n-hexane (2/1), Yield; 88%. ^1^H-NMR (400 MHz, CDCl_3_) ppm δ = 8.35–8.32 (m, AA’BB’ system, 2 H, Ar-H), 7.80–7.76 (m, 3 H, Ar-H), 7.70–7.65 (m, 3 H, Ar-H), 7.52–7.47 (m, 2 H, Ar-H), 7.44–7.38 (m, 2 H, Ar-H), 3.74 (bs, 2 H, -CH_2_-), 3.42 (bs, 2 H, -CH_2_-), 1.76–1.66 (m, 4 H, -CH_2_- (x2)), 1.61–1.57 (m, 2 H, -CH_2_-). ^13^C-NMR (100 MHz, CDCl_3_) ppm δ = 169.7, 164.2, 150.5, 144.6, 143.1, 139.9, 133.4, 129.0, 128.2, 128.1, 127.6, 127.2, 125.5, 123.7, 119.8, 109.7, 49.0, 42.1, 30.9, 24.6. HRMS [M + H] (C_25_H_23_N_2_O_2_): Calculated: 383.1760; Found: 383.1769.

### (2-([1,1’-Biphenyl]-4-yl)benzo[***d***]oxazol-6-yl)(morpholino)methanone (18)

Light yellow solid, m.p. 180–181°C, R_*f*_; 0.90 in ethylacetate/n-hexane (3/1), Yield; 79%. ^1^H-NMR (400 MHz, CDCl_3_) ppm δ = 8.36–8.32 (m, AA’BB’ system, 2 H, Ar-H), 7.82–7.77 (m, 3 H, Ar-H), 7.70 (dd, *J* = 0.6 Hz, *J* = 1.5 Hz, 1H, Ar-H), 7.69–7.66 (m, 2 H, Ar-H), 7.52–7.47 (m, 2 H, Ar-H), 7.45–7.41 (m, 2 H, Ar-H), 3.85–3.55 (m, 8 H, -CH_2_- (x4)). ^13^C-NMR (100 MHz, CDCl_3_) ppm δ = 169.8, 164.5, 150.5, 144.8, 143.6, 139.8, 132.1, 129.0, 128.3, 128.2, 127.7, 127.2, 125.4, 123.9, 120.0, 110.1, 66.9. HRMS [M + H] (C_24_H_21_N_2_O_3_): Calculated: 385.1552; Found: 385.1567.

### ***N,N***dimethyl-2-(naphthalen-1-yl)benzo[***d***]oxazole-6-carboxamide (19)

Light orange solid, m.p. 134–136 °C, R_*f*_; 0.76 in ethylacetate/n-hexane (2/1), Yield; 81%. ^1^H-NMR (400 MHz, CDCl_3_) ppm δ = 9.46 (d, *J* = 8.7 Hz, 1H, Ar-H), 8.43 (dd, *J* = 1.2 Hz, *J* = 7.3 Hz, 1H, Ar-H), 8.03 (d, *J* = 8.2 Hz, 1H, Ar-H), 7.92 (d, *J* = 8.2 Hz, 1H, Ar-H), 7.87 (dd, *J* = 0.6 Hz, *J* = 8.2 Hz, 1H, Ar-H), 7.73 (m, 2 H, Ar-H), 7.61–7.56 (m, 2 H, Ar-H), 7.46 (dd, *J* = 1.5 Hz, *J* = 8.2 Hz, 1H, Ar-H), 3.15 (bs, 3 H, -CH_3_), 3.04 (bs, 3 H, -CH_3_). ^13^C-NMR (100 MHz, CDCl_3_) ppm δ = 171.0, 164.2, 149.7, 143.3, 133.9, 133.4, 132.7, 130.6, 129.6, 128.7, 128.0, 126.5, 126.2, 124.9, 123.9, 123.1, 120.0, 109.9, 39.8, 35.6. HRMS [M + H] (C_20_H_17_N_2_O_2_): Calculated: 317.1290; Found: 317.1277.

### ***N,N***diethyl-2-(naphthalen-1-yl)benzo[***d***]oxazole-6-carboxamide (20)

Dark orange viscous liquid, R_*f*_; 0.92 in ethylacetate/n-hexane (3/1), Yield; 63%. ^1^H-NMR (400 MHz, CDCl_3_) ppm δ = 9.47 (dd, *J* = 0.7 Hz, *J* = 8.4 Hz, 1H, Ar-H), 8.44 (dd, *J* = 1.3 Hz, *J* = 7.3 Hz, 1H, Ar-H), 8.05 (d, *J* = 8.4 Hz, 1H, Ar-H), 7.94 (d, *J* = 8.4 Hz, 1H, Ar-H), 7.88 (dd, *J* = 0.7 Hz, *J* = 8.4 Hz, 1H, Ar-H), 7.74–7.68 (m, 2 H, Ar-H), 7.64–7.56 (m, 2 H, Ar-H), 7.42 (dd, *J* = 1.3 Hz, *J* = 8.4 Hz, 1H, Ar-H), 3.57 (bs, 2 H, -CH_2_-), 3.34 (bs, 2 H, -CH_2_-), 1.35 (bs, 3 H, -CH_3_), 1.12 (bs, 3 H, -CH_3_).^13^C-NMR (100 MHz, CDCl_3_) ppm δ = 170.6, 164.0, 149.8, 143.0, 134.4, 134.0, 132.7, 130.6, 129.5, 128.7, 128.0, 126.5, 126.2, 124.9, 123.2, 123.1, 120.2, 109.0, 43.4, 39.5, 14.1, 13.0. HRMS [M + H] (C_22_H_21_N_2_O_2_): Calculated: 345.1603; Found: 345.1625.

### 2-(Naphthalen-1-yl)-***N,N***dipropylbenzo[***d***]oxazole-6-carboxamide (21)

Light yellow viscous liquid, R_*f*_; 0.84 in ethylacetate/n-hexane (1/1), Yield; 91%. ^1^H-NMR (400 MHz, CDCl_3_) ppm δ = 8.79 (s, 1H, Ar-H), 8.31 (dd, *J* = 1.7 Hz, *J* = 8.6 Hz, 1H, Ar-H), 8.02–7.97 (m, 2 H, Ar-H), 7.93–7.88 (m, 1H, Ar-H), 7.81 (dd, *J* = 0.6 Hz, *J* = 8.1 Hz, 1H, Ar-H), 7.67–7.65 (m, 1H, Ar-H), 7.62–7.56 (m, 2 H, Ar-H), 7.38 (dd, *J* = 1.5 Hz, *J* = 8.1 Hz, 1H, Ar-H), 3.50 (bs, 2 H, -CH_2_-), 3.23 (bs, 2 H, -CH_2_-), 1.73 (bs, 2 H, -CH_2_-), 1.57 (bs, 2 H, -CH_2_-), 1.01 (bs, 3 H, -CH_3_), 0.76 (bs, 3 H, -CH_3_). ^13^C-NMR (100 MHz, CDCl_3_) ppm δ = 171.1, 164.4, 150.5, 142.8, 134.9, 134.4, 132.9, 129.0, 128.9, 128.4, 128.0, 127.9, 127.0,0 124.0, 123.9, 123.4, 119.9, 109.3, 50.9, 46.5, 21.9, 20.7, 11.4, 11.1. HRMS [M + H] (C_24_H_25_N_2_O_2_): Calculated: 373.1916; Found: 373.1925.

### (2-(Naphthalen-1-yl)benzo[***d***oxazol-6-yl)(pyrrolidin-1-yl)methanone (22)

Light brown solid, m.p. 165–166 °C, R_*f*_; 0.90 in ethylacetate/n-hexane (4/1), Yield; 56%. ^1^H-NMR (400 MHz, CDCl_3_) ppm δ = 9.47 (dd, *J* = 1.0 Hz, *J* = 7.9 Hz, 1H, Ar-H), 8.46 (dd, *J* = 1.0 Hz, *J* = 7.9 Hz, 1H, Ar-H), 8.06 (d, *J* = 8.2 Hz, 1H, Ar-H), 7.95 (d, *J* = 8.2 Hz, 1H, Ar-H), 7.88 (dd, *J* = 0.6 Hz, *J* = 8.2 Hz, 1H, Ar-H), 7.84 (dd, *J* = 0.6 Hz, *J* = 1.5 Hz, 1H, Ar-H), 7.75–7.70 (m, 1H, Ar-H), 7.64–7.58 (m, 3 H, Ar-H), 3.71 (t, *J* = 6.8 Hz, 2 H, -CH_2_-), 3.52 (t, *J* = 6.8 Hz, 2 H, -CH_2_-), 2.04–1.98 (m, 2 H, -CH_2_-), 1.95–1.88 (m, 2 H, -CH_2_-). ^13^C-NMR (100 MHz, CDCl_3_) ppm δ = 169.0, 164.3, 149.7, 143.5, 134.4, 134.0, 132.7, 130.7, 129.6, 128.7, 128.1, 126.5, 126.2, 124.9, 123.9, 123.2, 119.9, 109.9, 49.9, 46.4, 26.5, 24.5. HRMS [M + H] (C_22_H_19_N_2_O_2_): Calculated: 343.1447; Found: 343.1437.

### (2-(Naphthalen-1-yl)benzo[***d***oxazol-6-yl)(piperidin-1-yl)methanone (23)

Light yellow solid, m.p. 158–159 °C, R_*f*_; 0.70 in ethylacetate/n-hexane (1/1), Yield; 68%. ^1^H-NMR (400 MHz, CDCl_3_) ppm δ = 9.47 (d, *J* = 8.7 Hz, 1H, Ar-H), 8.46 (dd, *J* = 1.2 Hz, *J* = 7.4 Hz, 1H, Ar-H), 8.06 (d, *J* = 8.2 Hz, 1H, Ar-H), 7.95 (d, *J* = 8.2 Hz, 1H, Ar-H), 7.88 (d, *J* = 8.2 Hz, 1H, Ar-H), 7.75–7.69 (m, 2 H, Ar-H), 7.65–7.58 (m, 2 H, Ar-H), 7.45 (dd, *J* = 1.2 Hz, *J* = 8.2 Hz, 1H, Ar-H), 3.76 (bs, 2 H, -CH_2_-), 3.44 (bs, 2 H, -CH_2_-), 1.76–1.67 (m, 4 H, -CH_2_- (x2)), 1.62–1.52 (m, 2 H, -CH_2_-). ^13^C-NMR (100 MHz, CDCl_3_) ppm δ = 169.7, 164.1, 149.8, 143.2, 134.0, 133.6, 132.7, 130.7, 129.6, 128.7, 128.0, 126.5, 126.2, 124.9, 123.6, 123.2, 120.1, 109.6, 49.9, 43.5, 26.9, 25.6. HRMS [M + H] (C_23_H_21_N_2_O_2_): Calculated: 357.1603; Found: 357.1611.

### Morpholino(2-(naphthalen-1-yl)benzo[***d***]oxazol-6-yl)methanone (24)

Light gray solid, m.p. 153–154 °C, R_*f*_; 0.80 in ethylacetate/n-hexane (1/1), Yield; 70%. ^1^H-NMR (400 MHz, CDCl_3_) ppm δ = 9.47 (d, *J* = 8.6 Hz, 1H, Ar-H), 8.46 (d, *J* = 7.4 Hz, 1H, Ar-H), 8.07 (d, *J* = 8.1 Hz, 1H, Ar-H), 7.96 (d, *J* = 7.5 Hz, 1H, Ar-H), 7.90 (d, *J* = 8.1 Hz, 1H, Ar-H), 7.76–7.70 (m, 2 H, Ar-H), 7.66–7.58 (m, 2 H, Ar-H), 7.48–7.45 (m, 1H, Ar-H), 3.87–3.54 (m, 8 H, -CH_2_-). ^13^C-NMR (100 MHz, CDCl_3_) ppm δ = 169.9, 164.4, 149.8, 143.6, 134.0, 132.9, 132.3, 130.6, 129.7, 128.8, 128.1, 126.6, 126.1, 124.9, 123.8, 123.0, 120.3, 110.0, 66.9, 65.8. HRMS [M + H] (C_22_H_19_N_2_O_3_): Calculated: 359.1396; Found: 359.1415.

### ***N,N***dimethyl-2-(pyridin-4-yl)benzo[***d***]oxazole-6-carboxamide (25)

White solid, m.p. 157–158 °C, R_*f*_; 0.90 in ethylacetate/n-hexane (3/1), Yield; 96%. ^1^H-NMR (400 MHz, CDCl_3_) ppm δ = 8.81 (dd, *J* = 1.6 Hz, *J* = 4.5 Hz, 2 H, Ar-H), 8.06 (dd, *J* = 1.6 Hz, *J* = 4.5 Hz, 2 H, Ar-H), 7.81 (dd, *J* = 8.2 Hz, 1H, Ar-H), 7.70 (d, *J* = 1.2 Hz, 1H, Ar-H), 7.45 (dd, *J* = 1.2 Hz, *J* = 8.2 Hz, 1H, Ar-H), 3.13 (bs, 3 H, -CH_3_), 3.01 (bs, 3 H, -CH_3_). ^13^C-NMR (100 MHz, CDCl_3_) ppm δ = 170.5, 161.9, 150.8, 150.4, 142.6, 134.5, 133.9, 124.4, 121.0, 120.5, 110.3, 39.7, 35.6. HRMS [M + H] (C_15_H_14_N_3_O_2_): Calculated: 268.1086; Found: 268.1104.

### ***N,N***diethyl-2-(pyridin-4-yl)benzo[d]oxazole-6-carboxamide (26)

Gray solid, m.p. 110–111 °C, R_*f*_; 0.85 in ethylacetate/n-hexane (1/1), Yield; 84%. ^1^H-NMR (400 MHz, CDCl_3_) ppm δ = 8.81 (dd, *J* = 1.6 Hz, *J* = 4.5 Hz, 2 H, Ar-H), 8.06 (dd, *J* = 1.6 Hz, *J* = 4.5 Hz, 2 H, Ar-H), 7.81 (d, *J* = 8.2 Hz, 1H, Ar-H), 7.65 (d, *J* = 1.2 Hz, 1H, Ar-H), 7.40 (dd, *J* = 1.2 Hz, *J* = 8.2 Hz, 1H, Ar-H), 3.56 (bs, 2 H, -CH_2_-), 3.28 (bs, 2 H, -CH_2_-), 1.25 (bs, 3 H, -CH_3_), 1.13 (bs, 3 H, -CH_3_). ^13^C-NMR (100 MHz, CDCl_3_) ppm δ = 170.1, 161.8, 150.8, 150.5, 142.3, 135.5, 133.9, 123.7, 121.0, 120.7, 109.5, 43.4, 39.5, 14.1, 12.9. HRMS [M + H] (C_17_H_18_N_3_O_2_): Calculated: 296.1399; Found: 296.1413.

### ***N,N***dipropyl-2-(pyridin-4-yl)benzo[***d***]oxazole-6-carboxamide (27)

White solid, m.p. 95–96 °C, R_*f*_; 0.95 in ethylacetate/n-hexane (3/1), Yield; 98%. ^1^H-NMR (400 MHz, CDCl_3_) ppm δ = 8.83 (dd, *J* = 1.6 Hz, *J* = 4.5 Hz, 2 H, Ar-H), 8.09 (dd, *J* = 1.6 Hz, *J* = 4.5 Hz, 2 H, Ar-H), 7.83 (dd, *J* = 0.6 Hz, *J* = 8.2 Hz, 1H, Ar-H), 7.65 (dd, *J* = 0.6 Hz, *J* = 1.5 Hz, 1H, Ar-H), 7.41 (dd, *J* = 1.5 Hz, *J* = 8.2 Hz, 1H, Ar-H), 3.49 (bs, 2 H, -CH_2_-), 3.20 (bs, 2 H, -CH_2_-), 1.72 (bs, 2 H, -CH_2_-), 1.55 (bs, 2 H, -CH_2_-), 1.00 (bs, 3 H, -CH_3_), 0.75 (bs, 2 H, -CH_3_). ^13^C-NMR (100 MHz, CDCl_3_) ppm δ = 170.6, 161.8, 150.8, 150.5, 142.2, 135.6, 134.0, 123.8, 121.0, 120.6, 109.6, 50.9, 21.9, 20.7, 11.4, 11.1. HRMS [M + H] (C_19_H_22_N_3_O_2_): Calculated: 324.1712; Found: 324.1728.

### (2-(Pyridin-4-yl)benzo[***d***oxazol-6-yl)(pyrrolidin-1-yl)methanone (28)

White solid, m.p. 144–145 °C, R_*f*_; 0.60 in ethylacetate/n-hexane (1/1), Yield; 90%. ^1^H-NMR (400 MHz, CDCl_3_) ppm δ = 8.90–8.76 (m, 2 H, Ar-H), 8.08 (dd, *J* = 1.5 Hz, *J* = 4.6 Hz, 2 H, Ar-H), 7.83–7.79 (m, 2 H, Ar-H), 7.58 (dd, *J* = 1.4 Hz, *J* = 8.3 Hz, 1H, Ar-H), 3.68 (t, *J* = 6.8 Hz, 2 H, -CH_2_-), 3.47 (t, *J* = 6.8 Hz, 2 H, -CH_2_-), 2.02–1.96 (m, 2 H, -CH_2_-), 1.93–1.86 (m, 2 H, -CH_2_-). ^13^C-NMR (100 MHz, CDCl_3_) ppm δ = 168.6, 162.0, 150.8, 150.4, 142.8, 135.4, 133.9, 124.5, 121.1, 120.4, 120.3, 110.3, 49.8, 46.4, 26.5, 24.5. HRMS [M + H] (C_17_H_16_N_3_O_2_): Calculated: 294.1243; Found: 294.1255.

### Piperidin-1-yl(2-(pyridin-4-yl)benzo[***d***]oxazol-6-yl)methanone (29)

Gray solid, m.p. 127–128 °C, R_*f*_; 0.75 in ethylacetate/n-hexane (2/1), Yield; 87%. ^1^H-NMR (400 MHz, CDCl_3_) ppm δ = 8.83 (d, *J* = 5.1 Hz, 2 H, Ar-H), 8.08 (dd, *J* = 5.1 Hz, *J* = 7.3 Hz, 2 H, Ar-H), 7.82 (dd, *J* = 0.6 Hz, *J* = 8.2 Hz, 1H, Ar-H), 7.69 (dd, *J* = 0.6 Hz, *J* = 1.4 Hz, 1H, Ar-H), 7.44 (dd, *J* = 1.4 Hz, *J* = 8.2 Hz, 1H, Ar-H), 3.73 (bs, 2 H, -CH_2_-), 3.38 (bs, 2 H, -CH_2_-), 1.70–146 (m, 6 H). ^13^C-NMR (100 MHz, CDCl_3_) ppm δ = 169.2, 161.9, 150.8, 150.5, 142.5, 134.7, 133.9, 124.1, 121.0, 120.6, 110.0, 49.0, 43.5, 26.4, 25.6, 24.5. HRMS [M + H] (C_18_H_18_N_3_O_2_): Calculated: 308.1399; Found: 308.1407.

### Morpholino(2-(pyridin-4-yl)benzo[***d***]oxazol-6-yl)methanone (30)

Pink solid, m.p. 214–215 °C, R_*f*_; 0.70 in ethylacetate/n-hexane (2/1), Yield; 73%. ^1^H-NMR (400 MHz, CDCl_3_) ppm δ = 8.85 (dd, *J* = 1.7 Hz, *J* = 4.5 Hz, 2 H, Ar-H), 8.09 (dd, *J* = 1.7 Hz, *J* = 4.5 Hz, 2 H, Ar-H), 7.85 (dd, *J* = 0.6 Hz, *J* = 8.2 Hz, 1H, Ar-H), 7.73 (dd, *J* = 0.6 Hz, *J* = 1.5 Hz, 1H, Ar-H), 7.46 (dd, *J* = 1.5 Hz, *J* = 8.2 Hz, 1H, Ar-H), 4.01–3.94 (m, 1H), 3.85–3.52 (m, 7 H). ^13^C-NMR (100 MHz, CDCl_3_) ppm δ = 169.4, 162.2, 150.9, 143.0, 133.8, 133.4, 124.3, 121.1, 120.8, 112.7, 110.5, 66.8, 52.5. HRMS [M + H] (C_17_H_16_N_3_O_3_): Calculated: 310.1192; Found: 310.1181.

### ***N,N***-dimethyl-2-(1 H-pyrrol-2-yl)benzo[***d***]oxazole-6-carboxamide (31)

Dark brown solid, m.p. 195–196 °C, R_*f*_; 0.50 in ethylacetate/n-hexane (1/1), Yield; 52%. ^1^H-NMR (400 MHz, CDCl_3_) ppm δ = 10.62 (bs, 1H, -NH), 7.63 (dd, *J* = 3.0 Hz, *J* = 4.8 Hz, 2 H, Ar-H), 7.39 (dd, *J* = 1.4 Hz, *J* = 8.2 Hz, 1H, Ar-H), 7.13–7.09 (m, 1H, Ar-H), 7.06–7.02 (m, 1H, Ar-H), 6.40–6.35 (m, 1H, Ar-H), 3.14 (bs, 3 H, -CH_3_), 3.04 (bs, 3 H, -CH_3_). ^13^C-NMR (100 MHz, CDCl_3_) ppm δ = 171.0, 159.4, 149.7, 143.0, 132.3, 124.0, 123.5, 119.4, 118.4, 113.9, 111.0, 109.8, 39.8, 35.6. HRMS [M + H] (C_14_H_14_N_3_O_2_): Calculated: 256.1086; Found: 256.1097.

### ***N,N***-diethyl-2-(1 H-pyrrol-2-yl)benzo[***d***]oxazole-6-carboxamide (32)

Brown solid, m.p. 137–138 °C, R_*f*_; 0.70 in ethylacetate/n-hexane (3/1), Yield; 49%. ^1^H-NMR (400 MHz, CDCl_3_) ppm δ = 10.72 (bs, 1H, -NH), 7.62 (dd, *J* = 0.6 Hz, *J* = 8.1 Hz, 1H, Ar-H), 7.57 (dd, *J* = 0.6 Hz, *J* = 1.5 Hz, 1H, Ar-H), 7.34 (dd, *J* = 1.5 Hz, *J* = 8.1 Hz, 1H, Ar-H), 7.13–7.08 (m, 1H, Ar-H), 7.06–7.01 (m, 1H, Ar-H), 6.37 (dd, *J* = 2.7 Hz, *J* = 3.6 Hz, 1H, Ar-H), 3.56 (bs, 2 H, -CH_2_-), 3.33 (bs, 2 H, -CH_2_-), 1.37–1.08 (m, 6 H, - CH_3_(2x)). ^13^C-NMR (100 MHz, CDCl_3_) ppm δ = 170.7, 159.3, 149.7, 142.6, 133.3, 123.5, 123.2, 119.4, 118.6, 113.8, 111.0, 108.9, 43.4, 39.5, 14.1, 13.0. HRMS [M + H] (C_16_H_18_N_3_O_2_): Calculated: 284.1399; Found: 284.1409.

### ***N,N***-dipropyl-2-(1 H-pyrrol-2-yl)benzo[***d***]oxazole-6-carboxamide (33)

Brown viscous liquid, R_*f*_; 0.90 in ethylacetate/n-hexane (3/1), Yield; 76%. ^1^H-NMR (400 MHz, CDCl_3_) ppm δ = 10.99 (bs, 1H, -NH), 7.61 (dd, *J* = 0.4 Hz, *J* = 8.1 Hz, 1H, Ar-H), 7.56 (d, *J* = 1.3 Hz, 1H, Ar-H), 7.31 (dd, *J* = 1.3 Hz, *J* = 8.1 Hz, 1H, Ar-H), 7.10 (dd, *J* = 1.3 Hz, *J* = 3.7 Hz, 1H, Ar-H), 7.05–6.99 (m, 1H, Ar-H), 7.06–7.01 (m, 1H, Ar-H), 6.36 (dd, *J* = 2.6 Hz, *J* = 3.7 Hz, 1H, Ar-H), 3.48 (bs, 2 H, -CH_2_-), 3.22 (bs, 2 H, -CH_2_-), 1.70 (bs, 2 H, -CH_2_-), 1.56 (bs, 2 H, -CH_2_-), 0.98 (bs, 3 H, -CH_3_), 0.74 (bs, 3 H, -CH_3_). ^13^C-NMR (100 MHz, CDCl_3_) ppm δ = 171.2, 159.4, 149.7, 142.5, 133.4, 123.6, 123.4, 119.3, 118.5, 113.9, 110.9, 109.1, 50.9, 46.6, 21.9, 20.8, 11.3, 11.1. HRMS [M + H] (C_18_H_22_N_3_O_2_): Calculated: 312.1712; Found: 312.1725.

### (2-(1 H-pyrrol-2-yl)benzo[***d***]oxazol-6-yl)(pyrrolidin-1-yl)methanone (34)

Light yellow solid, m.p. 207–208 °C, R_*f*_; 0.80 in ethylacetate/n-hexane (1/1), Yield; 65%. ^1^H-NMR (400 MHz, CDCl_3_) ppm δ = 9.61 (bs, 1H, -NH), 7.72 (dd, *J* = 0.6 Hz, *J* = 1.5 Hz, 1H, Ar-H), 7.64 (dd, *J* = 0.6 Hz, *J* = 8.2 Hz, 1H, Ar-H), 7.52 (dd, *J* = 1.5 Hz, *J* = 8.2 Hz, 1H, Ar-H), 7.13–7.09 (m, 1H, Ar-H), 7.08–7.05 (m, 1H, Ar-H), 6.41–6.37 (m, 1H, Ar-H), 3.69 (t, *J* = 7.0 Hz, 2 H, -CH_2_-), 3.50 (t, *J* = 6.5 Hz, 2 H, -CH_2_-), 2.03–1.95 (m, 2 H, -CH_2_-), 1.93–1.87 (m, 2 H, -CH_2_-). ^13^C-NMR (100 MHz, CDCl_3_) ppm δ = 169.0, 159.1, 149.7, 143.2, 133.3, 124.1, 123.2, 119.5, 118.5, 113.6, 111.1, 109.7, 49.9, 46.4, 26.5, 24.5. HRMS [M + H] (C_16_H_16_N_3_O_2_): Calculated: 282.1243; Found: 282.1228.

### (2-(1 H-pyrrol-2-yl)benzo[***d***]oxazol-6-yl)(piperidin-1-yl)methanone (35)

White solid, m.p. 215–216 °C, R_*f*_; 0.82 in ethylacetate/n-hexane (1/1), Yield; 38%. ^1^H-NMR (400 MHz, CDCl_3_) ppm δ = 9.67 (bs, 1H, -NH), 7.64 (d, *J* = 8.1 Hz, 1H, Ar-H), 7.59 (d, *J* = 1.4 Hz, 1H, Ar-H), 7.37 (dd, *J* = 1.4 Hz, *J* = 8.1 Hz, 1H, Ar-H), 7.12–7.10 (m, 1H, Ar-H), 7.07–7.06 (m, 1H, Ar-H), 6.40–6.38 (m, 1H, Ar-H), 3.80–3.64 (m, 2 H, -CH_2_-), 3.47–3.33 (m, 2 H, -CH_2_-), 1.74–1.65 (m, 6 H, -CH_2_-(x3)). ^13^C-NMR (100 MHz, CDCl_3_) ppm δ = 169.7, 149.8, 142.9, 132.6, 123.7, 123.2, 119.5, 118.7, 118.3, 113.6, 111.1, 109.5, 47.9, 43.5, 31.6, 26.9, 24.6. HRMS [M + H] (C_17_H_18_N_3_O_2_): Calculated: 296.1399; Found: 296.1412.

### (2-(1 H-pyrrol-2-yl)benzo[***d***]oxazol-6-yl)(morpholino)methanone (36)

Orange solid, m.p. 135–136 °C, R_*f*_; 0.65 in ethylacetate/n-hexane (1/1), Yield; 44%. ^1^H-NMR (400 MHz, CDCl_3_) ppm δ = 12.33 (bs, 1H, -NH), 7.74 (dd, *J* = 0.6 Hz, *J* = 1.5 Hz, 1H, Ar-H), 7.69 (dd, *J* = 0.6 Hz, *J* = 8.1 Hz, 1H, Ar-H), 7.38 (dd, *J* = 1.5 Hz, *J* = 8.1 Hz, 1H, Ar-H), 7.15–7.11 (m, 1H, Ar-H), 6.99 (dd, *J* = 1.4 Hz, *J* = 3.6 Hz, 1H, Ar-H), 6.29 (dd, *J* = 2.5 Hz, *J* = 3.6 Hz, 1H, Ar-H), 3.72–3.44 (m, 8 H, -CH_2_-). ^13^C-NMR (100 MHz, CDCl_3_) ppm δ = 169.0, 159.4, 149.5, 143.3, 131.9, 125.1, 124.5, 118.9, 118.8, 113.9, 110.8, 110.0, 66.5. HRMS [M + H] (C_16_H_16_N_3_O_3_): Calculated: 298.1192; Found: 298.1224.

### 2-(Furan-2-yl)-N,N-dimethylbenzo[***d***]oxazole-6-carboxamide (37)

Light brown solid, m.p. 94–95 °C, R_*f*_; 0.84 in ethylacetate/n-hexane (1/1), Yield; 86%. ^1^H-NMR (400 MHz, CDCl_3_) ppm δ = 7.72 (dd, *J* = 0.6 Hz, *J* = 8.2 Hz, 1H, Ar-H), 7.66 (dd, *J* = 0.8 Hz, *J* = 1.8 Hz, 1H, Ar-H), 7.63 (dd, *J* = 0.6 Hz, *J* = 1.5 Hz, 1H, Ar-H), 7.40 (dd, *J* = 1.5 Hz, *J* = 8.2 Hz, 1H, Ar-H), 7.28 (dd, *J* = 0.8 Hz, *J* = 3.5 Hz, 1H, Ar-H), 6.60 (dd, *J* = 1.8 Hz, *J* = 3.5 Hz, 1H, Ar-H), 3.11 (bs, 3 H, -CH_3_), 3.00 (bs, 3 H, -CH_3_). ^13^C-NMR (100 MHz, CDCl_3_) ppm δ = 170.7, 156.5, 149.7, 146.1, 142.7, 142.2, 133.4, 124.2, 119.8, 115.0, 112.4, 109.9, 39.7, 35.5. HRMS [M + H] (C_14_H_13_N_2_O_3_): Calculated: 257.0926; Found: 257.0944.

### ***N,N***-diethyl-2-(furan-2-yl)benzo[***d***]oxazole-6-carboxamide (38)

Dark brown solid, m.p. 101–102 °C, R_*f*_; 0.75 in ethylacetate/n-hexane (1/2), Yield; 79%. ^1^H-NMR (400 MHz, CDCl_3_) ppm δ = 7.73 (dd, *J* = 0.7 Hz, *J* = 8.1 Hz, 1H, Ar-H), 7.67–7.65 (m, 1H, Ar-H), 7.59–7.57 (m, 1H, Ar-H), 7.36 (dd, *J* = 1.6 Hz, *J* = 8.1 Hz, 1H, Ar-H), 7.29–7.27 (m, 1H, Ar-H), 6.60 (dd, *J* = 1.6 Hz, *J* = 3.5 Hz, 1H, Ar-H), 3.54 (bs, 2 H, -CH_2_-), 3.28 (bs, 2 H, -CH_2_-), 1.22 (bs, 3 H, -CH_3_), 1.07 (bs, 3 H, -CH_3_). ^13^C-NMR (100 MHz, CDCl_3_) ppm δ = 170.4, 156.3, 149.8, 146.1, 142.3, 142.2, 134.4, 123.4, 120.0, 114.9, 112.4, 109.1, 43.4, 39.5, 14.1, 12.9. HRMS [M + H] (C_16_H_17_N_2_O_3_): Calculated: 285.1239; Found: 285.1251.

### 2-(Furan-2-yl)-***N,N***-dipropylbenzo[***d***]oxazole-6-carboxamide (39)

Light Brown viscous liquid, R_*f*_; 0.88 in ethylacetate/n-hexane (1/3), Yield; 94%. ^1^H-NMR (400 MHz, CDCl_3_) ppm δ = 7.71 (dd, *J* = 0.58 Hz, *J* = 8.13 Hz, 1H, Ar-H), 7.65 (dd, *J* = 0.78 Hz, *J* = 1.75 Hz, 1H, Ar-H), 7.55 (dd, *J* = 0.58 Hz, *J* = 1.45 Hz, 1H, Ar-H), 7.33 (dd, *J* = 1.45 Hz, *J* = 8.13 Hz, 1H, Ar-H), 7.27 (dd, *J* = 0.78 Hz, *J* = 3.54 Hz, 1H, Ar-H), 6.59 (dd, *J* = 1.75 Hz, *J* = 3.54 Hz, 1H, Ar-H), 3.44 (bs, 2 H, -CH_2_-), 3.16 (bs, 2 H, -CH_2_-), 1.67 (bs, 2 H, -CH_2_-), 1.52 (bs, 2 H, -CH_2_-), 0.95 (bs, 3 H, -CH_3_), 0.70 (bs, 3 H, -CH_3_). ^13^C-NMR (100 MHz, CDCl_3_) ppm δ = 170.8, 156.3, 149.8, 149.7, 146.1, 142.2, 134.5, 123.6, 120.0, 114.9, 112.4, 109.3, 50.8, 46.5, 21.9, 20.7, 11.4, 11.0. HRMS [M + H] (C_18_H_21_N_2_O_3_): Calculated: 313.1552; Found: 313.1518.

### (2-(Furan-2-yl)benzo[***d***oxazol-6-yl)(pyrrolidin-1-yl)methanone (40)

White solid, m.p. 98–100 °C, R_*f*_; 0.90 in ethylacetate/n-hexane (1/1), Yield; 65%. ^1^H-NMR (400 MHz, CDCl_3_) ppm δ = 7.77–7.74 (m, 2 H, Ar-H), 7.70 (dd, *J* = 0.8 Hz, *J* = 1.8 Hz, 1H, Ar-H), 7.55 (dd, *J* = 1.4 Hz, *J* = 8.3 Hz, 1H, Ar-H), 7.32 (dd, *J* = 0.8 Hz, *J* = 3.5 Hz, 1H, Ar-H), 6.64 (dd, *J* = 1.8 Hz, *J* = 3.5 Hz, 1H, Ar-H), 3.68 (t, *J* = 6.8 Hz, 2 H, -CH_2_-), 3.48 (t, *J* = 6.8 Hz, 2 H, -CH_2_-), 2.01–1.96 (m, 2 H, -CH_2_-), 1.93–1.87 (m, 2 H, -CH_2_-). ^13^C-NMR (100 MHz, CDCl_3_) ppm δ = 168.8, 156.6, 149.7, 146.1, 142.9, 142.3, 134.4, 124.3, 119.7, 115.0, 112.4, 110.0, 49.9, 46.4, 26.5, 24.4. HRMS [M + H] (C_16_H_15_N_2_O_3_): Calculated: 283.1083; Found: 283.1107.

### (2-(Furan-2-yl)benzo[***d***oxazol-6-yl)(piperidin-1-yl)methanone (41)

Light Brown viscous liquid, R_*f*_; 0.90 in ethylacetate/n-hexane (1/1), Yield; 87%. ^1^H-NMR (400 MHz, CDCl_3_) ppm δ = 7.76 (dd, *J* = 0.6 Hz, *J* = 8.2 Hz, 1H, Ar-H), 7.69 (dd, *J* = 0.8 Hz, *J* = 1.8 Hz, 1H, Ar-H), 7.63 (dd, *J* = 0.6 Hz, *J* = 1.5 Hz, 1H, Ar-H), 7.40 (dd, *J* = 1.5 Hz, *J* = 8.2 Hz, 1H, Ar-H), 7.32 (dd, *J* = 0.8 Hz, *J* = 3.5 Hz, 1H, Ar-H), 6.64 (dd, *J* = 1.8 Hz, *J* = 3.5 Hz, 1H, Ar-H), 3.81–3.65 (bs, 2 H, -CH_2_-), 3.46–3.33 (bs, 2 H, -CH_2_-), 1.75–1.65 (m, 2 H, -CH_2_-), 1.61–1.51 (bs, 4 H, -CH_2_-(x2)). ^13^C-NMR (100 MHz, CDCl_3_) ppm δ = 169.5, 156.4, 149.8, 146.1, 142.6, 142.3, 133.6, 123.9, 120.0, 115.0, 112.4, 109.7, 49.1, 43.5, 29.7, 26.5, 24.6. HRMS [M + H] (C_17_H_17_N_2_O_3_): Calculated: 297.1239; Found: 297.1244.

### (2-(Furan-2-yl)benzo[***d***oxazol-6-yl)(morpholino)methanone (42)

Light orange solid, m.p. 108–109 °C, R_*f*_; 0.72 in ethylacetate/n-hexane (1/1), Yield; 81%. ^1^H-NMR (400 MHz, CDCl_3_) ppm δ = 7.78 (dd, *J* = 0.6 Hz, *J* = 8.1 Hz, 1H, Ar-H), 7.70 (dd, *J* = 0.8 Hz, *J* = 1.8 Hz, 1H, Ar-H), 7.66 (0.6 Hz, *J* = 1.5 Hz, 1H, Ar-H), 7.42 (dd, *J* = 1.5 Hz, *J* = 8.1 Hz, 1H, Ar-H), 7.33 (dd, *J* = 0.8 Hz, *J* = 3.5 Hz, 1H, Ar-H), 6.65 (dd, 1.8 Hz, *J* = 3.5 Hz, 1H, Ar-H), 3.88–3.36 (m, 8 H, -CH_2_-(x4)). ^13^C-NMR (100 MHz, CDCl_3_) ppm δ = 169.7, 156.7, 149.9, 146.2, 143.0, 142.1, 132.3, 124.2, 120.1, 115.2, 112.5, 110.1, 66.9. HRMS [M + H] (C_16_H_15_N_2_O_4_): Calculated: 299.1032; Found: 299.1061.

### ***N,N***dimethyl-2-(thiophen-2-yl)benzo[***d***]oxazole-6-carboxamide (43)

Light yellow solid, m.p.128–129 °C, R_*f*_; 0.84 in ethylacetate/n-hexane (1/1), Yield; 78%. ^1^H-NMR (400 MHz, CDCl_3_) ppm δ = 7.93 (dd, *J* = 1.2 Hz, *J* = 3.8 Hz, 1H, Ar-H), 7.72 (d, *J* = 8.2 Hz, 1H, Ar-H), 7.64 (d, *J* = 1.2 Hz, 1H, Ar-H), 7.58 (dd, *J* = 1.2 Hz, *J* = 5.0 Hz, 1H, Ar-H), 7.41 (dd, *J* = 1.2 Hz, *J* = 8.2 Hz, 1H, Ar-H), 7.19 (dd, *J* = 3.8 Hz, *J* = 5.0 Hz, 1H, Ar-H), 3.14 (bs, 3 H, -CH_3_), 3.02 (bs, 3 H, -CH_3_). ^13^C-NMR (100 MHz, CDCl_3_) ppm δ = 170.8, 160.3, 150.0, 143.1, 133.2, 130.9, 130.5, 129.2, 128.4, 124.1, 119.5, 109.8, 39.8, 35.6. HRMS [M + H] (C_14_H_13_N_2_O_2_S): Calculated: 273.0698; Found: 273.0729.

### ***N,N***-2-(thiophen-2-yl)benzo[***d***]oxazole-6-carboxamide (44)

Brown solid, m.p. 130–131 °C, R_*f*_; 0.95 in ethylacetate/n-hexane (1/1), Yield; 71%. ^1^H-NMR (400 MHz, CDCl_3_) ppm δ = 7.93 (dd, *J* = 1.4 Hz, *J* = 3.7 Hz, 1H, Ar-H), 7.73 (dd, *J* = 0.6 Hz, *J* = 8.1 Hz, 1H, Ar-H), 7.60–7.58 (m, 2 H, Ar-H), 7.37 (dd, *J* = 1.4 Hz, *J* = 8.1 Hz, 1H, Ar-H), 7.20 (dd, *J* = 3.7 Hz, *J* = 5.0 Hz, 1H, Ar-H), 3.56 (bs, 2 H, -CH_2_-), 3.32 (bs, 2 H, -CH_2_-), 1.29–1.11 (m, 6 H, -CH_3_(2x)). ^13^C-NMR (100 MHz, CDCl_3_) ppm δ = 170.5, 160.2, 150.1, 142.7, 134.2, 130.8, 130.4, 129.2, 128.3, 123.3, 119.7, 109.0, 43.4, 39.6, 14.1, 13.1. HRMS [M + H] (C_16_H_17_N_2_O_2_S): Calculated: 301.1011; Found: 301.1040.

### ***N,N***dipropyl-2-(thiophen-2-yl)benzo[***d***]oxazole-6-carboxamide (45)

Dark yellow viscous liquid, R_*f*_; 0.96 in ethylacetate/n-hexane (3/1), Yield; 85%. ^1^H-NMR (400 MHz, CDCl_3_) ppm δ = 7.92 (dd, *J* = 1.22 Hz, *J* = 3.75 Hz, 1H, Ar-H), 7.71 (d, *J* = 8.15 Hz, 1H, Ar-H), 7.58–7.56 (m, 2 H, Ar-H), 7.34 (dd, *J* = 1.45 Hz, *J* = 8.15 Hz, 1H, Ar-H), 7.19 (dd, *J* = 3.75 Hz, *J* = 4.95 Hz, 1H, Ar-H), 3.47 (bs, 2 H, -CH_2_-), 3.19 (bs, 2 H, -CH_2_-), 1.70 (bs, 2 H, -CH_2_-), 1.54 (bs, 2 H, -CH_2_-), 0.99 (bs, 3 H, -CH_3_), 0.73 (bs, 3 H, -CH_3_). ^13^C-NMR (100 MHz, CDCl_3_) ppm δ = 171.0, 160.1, 150.1, 142.6, 134.3, 130.8, 130.4, 129.2, 128.4, 123.5, 119.6, 109.2, 50.9, 46.5, 21.9, 20.7, 11.4, 11.1. HRMS [M + H] (C_18_H_21_N_2_O_2_S): Calculated: 329.1324; Found: 329.1310.

### Pyrrolidin-1-yl(2-(thiophen-2-yl)benzo[***d***]oxazol-6-yl)methanone (46)

Light brown solid, m.p. 140–141 °C, R_*f*_; 0.80 in ethylacetate/n-hexane (1/1), Yield; 80%. ^1^H-NMR (400 MHz, CDCl_3_) ppm δ = 7.94 (dd, *J* = 1.2 Hz, *J* = 3.8 Hz, 1H, Ar-H), 7.76–7.68 (m, 2 H, Ar-H), 7.60 (dd, *J* = 1.2 Hz, *J* = 5.0 Hz, 1H, Ar-H), 7.53 (dd, *J* = 1.5 Hz, *J* = 8.2 Hz, 1H, Ar-H), 7.21 (dd, *J* = 3.8 Hz, *J* = 5.0 Hz, 1H, Ar-H), 3.69 (t, *J* = 6.8 Hz, 2 H, -CH_2_-), 3.48 (t, *J* = 6.8 Hz, 2 H, -CH_2_-), 2.04–1.86 (m, 4 H, -CH_2_-(x2)). ^13^C-NMR (100 MHz, CDCl_3_) ppm δ = 168.9, 160.4, 150.0, 143.2, 134.1, 130.9, 130.5, 129.2, 128.4, 124.2, 119.4, 109.9, 49.9, 46.4, 26.5, 24.5. HRMS [M + H] (C_16_H_15_N_2_O_2_S): Calculated: 299.0854; Found: 299.0871.

### Piperidin-1-yl(2-(thiophen-2-yl)benzo[***d***]oxazol-6-yl)methanone (47)

Light yellow solid, m.p.151–152 °C, R_*f*_; 0.60 in ethylacetate/n-hexane (1/2), Yield; 75%. ^1^H-NMR (400 MHz, CDCl_3_) ppm δ = 7.94 (dd, *J* = 1.2 Hz, *J* = 3.7 Hz, 1H, Ar-H), 7.73 (dd, *J* = 0.6 Hz, *J* = 8.2 Hz, 1H, Ar-H), 7.62 (dd, *J* = 0.6 Hz, *J* = 1.5 Hz, 1H, Ar-H), 7.59 (dd, *J* = 1.2 Hz, *J* = 5.0 Hz, 1H, Ar-H), 7.39 (dd, *J* = 1.5 Hz, *J* = 8.2 Hz, 1H, Ar-H), 7.21 (dd, *J* = 3.7 Hz, *J* = 5.0 Hz, 1H, Ar-H), 3.72 (bs, 2 H, -CH_2_-), 3.40 (bs, 2 H, -CH_2_-), 1.75–1.66 (m, 4 H,-CH_2_-(x2)), 1.64–1.55 (m, 2 H, -CH_2_-). ^13^C-NMR (100 MHz, CDCl_3_) ppm δ = 169.6, 160.3, 150.1, 143.0, 133.4, 130.8, 130.5, 129.2, 128.4, 123.8, 119.6, 109.6, 48.9, 43.4, 26.1, 25.5, 24.6. HRMS [M + H] (C_17_H_17_N_2_O_2_S): Calculated: 313.1012; Found: 313.1059.

### Morpholino(2-(thiophen-2-yl)benzo[***d***]oxazol-6-yl)methanone (48)

Brown solid, m.p. 213–214 °C, R_*f*_; 0.85 in ethylacetate/n-hexane (2/1), Yield; 82%. ^1^H-NMR (400 MHz, CDCl_3_) ppm δ = 7.95 (dd, *J* = 1.2 Hz, *J* = 3.8 Hz, 1H, Ar-H), 7.75 (dd, *J* = 0.6 Hz, *J* = 8.1 Hz, 1H, Ar-H), 7.65 (dd, *J* = 0.6 Hz, *J* = 1.5 Hz, 1H, Ar-H), 7.61 (dd, *J* = 1.2 Hz, *J* = 5.0 Hz, 1H, Ar-H), 7.41 (dd, *J* = 1.5 Hz, *J* = 8.1 Hz, 1H, Ar-H), 7.21 (dd, *J* = 3.8 Hz, *J* = 5.0 Hz, 1H, Ar-H), 3.84–3.55 (m, 8 H, -CH_2_-(x4)). ^13^C-NMR (100 MHz, CDCl_3_) ppm δ = 169.7, 160.5, 150.1, 143.4, 132.0, 131.1, 130.7, 129.0, 128.4, 124.1, 119.8, 110.0, 66.9. HRMS [M + H] (C_16_H_15_N_2_O_3_S): Calculated: 315.0803; Found: 315.0788.

### Biochemistry

In the present work, the in vitro effects of 2-aryl-6-carboxamide benzoxazole derivatives (**7**–**48**) on AChE/BChE from electric eel (*Electrophorus electricus*)/equine serum was realized according to Ellman’s method (1961) as previously described [54]. These analogs were dissolved in DMSO at an initial concentration of 1 mg/mL. Around 1% of DMSO was present in the final reaction mixture. Acetylthiocholine iodide (AChI)/butyrylcholine iodide (BChI) were used as substrates for enzymatic reactions. For this aim, an aliquot (0.1 mL) of Tris/HCl buffer (pH 8.0, 1.0 M) and different 2-aryl-6-carboxamide benzoxazole derivatives (**7**–**48**) concentrations (10–30 µg/mL) were added to 50 µL of AChE/BChE solution (5.30 × 10^− 3^ EU). The mixture was incubated at 20 ^o^C for 20 min. Then, 50 µL of 5,5’-dithio-bis 2-nitro-benzoic acid (DTNB, 0.5 mM) and AChI/BChI were added and enzymatic reactions were initiated. Then, AChE/BChE activities were spectrophotometrically determined at 412 nm [55].

### AChE and BChE inhibition assay

The half maximal inhibition concentration (IC_50_) was calculated from activity (%) versus 2-aryl-6-carboxamide benzoxazole derivatives (**7**–**48**) plots [56]. Lineweaver-Burk graphs were used for the calculation of K_i_ and the other parameters as described in prior studies [57]. The K_i_ values were taken out from this graph [58]. All analyses were conducted independently in triplicate, and result data are expressed as mean values ± SD [59].

### Molecular docking

Molecular docking studies were carried out with Maestro (Schrodinger, 12.8.117). Compound **36**, which is the most active compound against AChE and BChE, was chosen for docking studies. The crystal structure of 1EVE pdb-encoded protein of the complex of donepezil in AChE was downloaded from https://www.rcsb.org/. The homology model of *Equus caballus* (horse) BChE was built [50, 60]. There is no crystal structure available for ECBChE. For this reason, a homology model of *Equus caballus* (horse) BChE (ECBChE) was constructed utilizing comparative modeling techniques. In homology modeling, Q9N1N9 was used as the sequence for ECBChE, and 4TPK was used as a template structure. Homology modeling was performed by simply aligning the sequences of these enzymes with the most homologous sequences of the proteins with available 3D structure, hBChE (homo sapiens), and threading the target model structures. Sequence identity between ECBChE and hBChE, co-crystallized ligand (3F9) in the active site of hBChE, and solvent molecules were transferred to the respective model structures using a specific MODELLER script. The structural quality of the model was confirmed by the PROCHECK analyses [50].

Water molecules, heteroatoms, and ligands in the structure of proteins have been removed, and hydrogen atoms were added to the protein by using Protein Preparation Wizard module. In this method, the ionization and tautomeric states were created by Epik (Schrodinger, 12.8.117), whereas the proton orientations were determined by PROPKA. Impact (Schrodinger, 12.8.117) was used to perform constrained reduction with a convergence of heavy atoms to an RMSD of 3 Å. The structures have become suitable for protein docking studies. The 20 Å sphere was identified as binding sites at active sites of proteins using the Receptor Grid Generation module. The 3D structures of the ligands were created with MacroModel. Ligands were prepared using LigPrep to include possible ionization and tautomeric states and optimized using OPLS4 force field and conjugate gradient method. Ligands were docked to the binding sites with standard precision (sp) 100 using the Glide program with default settings, and the docking scores were calculated [48, 49]. To confirm the validity of the docking procedure, E20 (donepezil) and 3F9 were re-docked to their respective binding sites; the RMSD values of the resulting docking postures were 0.80 and 0.91, respectively. Maestro (Schrodinger, 12.8.117) and the workspace ligand interaction diagram module were used to create 2D and 3D figures.

### Molecular dynamics simulations

Desmond (Schrodinger, 12.8.117) program was used in all molecular dynamics simulations. Molecular dynamics simulations were performed for 100 ns under NPT ensemble, for compound **36**, the most active compound against AChE and BChE. In these simulations, average RMSD values of ligands and proteins and interactions of ligands with residues were investigated. Ligands were solvated in an octahedral box with TIP3P water molecules leaving at least 10 Å. The systems were neutralized by Na^+^ and Cl^−^ counter ions and the concentrations of the systems were adjusted with 0.15 M NaCl solution. Desmond’s standard relaxation methodology was used, and before production simulations, prepared solvated simulation systems were first put through a series of minimizations and short MD simulations [49, 60]. The Nose-Hoover thermostat was used to regulate the systems’ temperature during MD simulations, and it was initially set to 300 K. With isotropic pressure coupling, the Martyna-Tobias-Klein method was used to regulate pressure around 1.01325 bar. 9 Å cutoff for the short-range nonbonded interactions was used combined with the particle mesh Ewald option [59–62].

### ADME study

Some predicted pharmacokinetic properties of synthesised compounds used in absorption, distribution, metabolism, and excretion (ADME) analyzes were calculated using QikProp (Maestro, Schrodinger, 12.8.117) and SwissADME http://www.swissadme.ch/ software [63].

## Electronic supplementary material

Below is the link to the electronic supplementary material.

Supplementary Material 1
