# Supplementary material for: Structure-based inhibition of acetylcholinesterase and butyrylcholinesterase with 2-Aryl-6-carboxamide benzoxazole derivatives: synthesis, enzymatic assay, and in silico studies
Source: Mol Divers. 2024 Mar 30;29(1):671–93. doi: 10.1007/s11030-024-10828-6 (PMC11785640; doi:10.1007/s11030-024-10828-6)
Supplement: Supplementary file 1 — Supplementary Material 1 [file 11030_2024_10828_MOESM1_ESM.docx]

Structure-Based Inhibition of Acetylcholinesterase and Butyrylcholinesterase with 2-Aryl-6-carboxamide Benzoxazole Derivatives: Synthesis, Enzymatic Assay, and *in silico* Studies

**Burak Kuzu,^a,b^ M. Abdullah Alagoz,^c^ Yeliz Demir,^d^ Ilhami Gulcin,^e^ Serdar Burmaoglu,*^e^ Oztekin Algul, *^a,f^**

^a^ Department of Pharmaceutical Chemistry, Faculty of Pharmacy, Mersin University, Mersin, 33169, Turkey

^b^Department of Pharmaceutical Chemistry, Faculty of Pharmacy, Van Yuzuncu Yil University, Van, 65080, Turkey

^c^Department of Pharmaceutical Chemistry, Faculty of Pharmacy, İnonu University, Malatya, 44280, Turkey

^d^Department of Pharmacy Services, Nihat Delibalta Göle Vocational High School, Ardahan University, 75000, Ardahan, Turkey

^e^Department of Chemistry, Faculty of Science, Ataturk University, Erzurum, 25240, Turkey

^f^Department of Pharmaceutical Chemistry, Faculty of Pharmacy, Erzincan Binali Yildirim University, Erzincan, 24100, Turkey

*Author for correspondence: oztekinalgul@mersin.edu.tr

**Supplementary Material**

| Contents | Pages |
| --- | --- |
| 1. General Information | **2** |
| 1. Synthesis of methyl 4-amino-3-hydroxybenzoate as a synthesis starting point | **2** |
| 1. General procedure for the synthesis of 2-aryl benzoxazole derivatives | **2** |
| 1. General procedure for the synthesis of 2-aryl-6-carboxamide benzoxazole derivatives | **3** |
| 1. ^1^H- and ^13^C-NMR spectrum copies of compounds 7-48 | **4** |
| 1. QTOF spectrum copies of compounds 7-48 | **46** |
| 1. Molecular Dynamics trajectory frame of compound 36 and Fraction of residue interactions for AChE and BChE | **60** |
| 1. Molecular dynamic simulations for donepezil inside AChE and BChE | **62** |
| 1. Predicted molecular properties of compounds 7-48 | **63** |
| 1. References | **64** |

1. **General Information**

Commercially available materials were used without further purification. ^1^H-NMR, ^13^C-NMR, spectra were recorded at 400, and 100 MHz, respectively, on a Varian-Agilent 400 MHz instrument using Me_4_Si as an internal standard. Kieselgel 60 F_254_ 2 mm thick coated ready-made aluminum plates (Silicycle) were used in thin layer chromatography (TLC) studies. All column chromatography was performed on silica gel (60 mesh, Silycycle). New compounds for HRMS were tested on a Thermo Scientific Q Exactive MS/MS system ESI spectrometer. Chemical shift multiplicities are represented as follows: (s = singlet, d = doublet, t = triple, dd = double doublet, and m = multiplet).

1. **Synthesis of methyl 4-amino-3-hydroxybenzoate as a synthesis starting point**

The 4-amino-3-hydroxy-benzoic acid (5 mmol) was dissolved in 10 ml of methanol and refluxed for 12 hours under the catalyst of a few drops of concentrated H_2_SO_4_. After the completion of the reaction was checked with TLC, the reaction was terminated and cooled to room temperature. It was neutralized with 50 ml of water and 1N NaHCO_3_ until pH:7.5. The resulting aqueous phase was extracted with 3x15 ml of ethyl acetate. The organic phase was separated and the solvent was evaporated and purified by column chromatography in the appropriate n-hexane/ethylacetate (5/1) mobile phase.**^1^**

1. **General procedure for the synthesis of 2-aryl benzoxazole derivatives**

A mixture of methyl 4-amino-3-hydroxybenzoate (1 mmol) was dissolved in 5 mL ethanole and the corresponding aromatic carboxaldehyde derivatives (1.1 mmol) were added to the reaction mixture. The reaction flask was stirred for a period of 6-24 hours at room temperature and the reaction completion was controlled with TLC method. After observing that the starting materials were disappeared, the reaction mixture was poured onto ice-water to get the precipitate. The resulting precipitate was filtered off and washed with cold water. The solid products were used in the second step without isolation. Solid products (1 mmol) were dissolved in 5 mL of DMF and a catalytic amount of NaCN was added. The reaction mixture was stirred at room temperature 1 h. With the starting material disappearing, the reaction mixture was transferred to the ice-water mixture to get the precipitate. The resulting precipitate was filtered off and washed with brine. The final products were purified with column chromatography 5:1 (n-hexane: ethyl acetate).**^2^**

1. **General procedure for the synthesis of 2-aryl-6-carboxamide benzoxazole derivatives**

2-Aryl benzoxazole derivatives (1 mmol) synthesized in the previous step were dissolved in 4 ml of dichloromethane (DCM) and 1.2 mmol of cyclic or linear aliphatic secondary amine (pyrrolidine, piperidine, morpholine or dimethylamine, diethylamine, dipropylamine) derivatives were added. After the reaction mixture was stirred at room temperature for 10 minutes, a catalytic amount of aluminum (III) chloride (AlCl_3_) was added. The reaction medium was stirred at room temperature for 24 hours. The completion of the reaction was checked with TLC and the reaction was terminated by the addition of water. The resulting mixture was extracted by adding 3x15 ml of dichloromethane (DCM) and 50 ml of water. The organic phases were collected, treated with MgSO_4_ and filtered. The crude product obtained by evaporation of the organic solvent was washed successively with diethyl ether and cyclohexane.

In fact, the reaction conditions were first adjusted in dichloromethane (DCM) at room temperature by addition of straight chain or cyclic secondary amines.**^3^** However, the reaction did not proceed at this temperature and the situation did not change with the increase in temperature. Therefore, in order to increase the reactivity of the ester carbonyl, AlCl_3_ was added to the reaction medium as a catalytic amount of lewis acid. The mechanism of the mentioned reaction is presented in the figure.

1. **^1^H- and ^13^C-NMR spectrum copies of compounds 7-48**


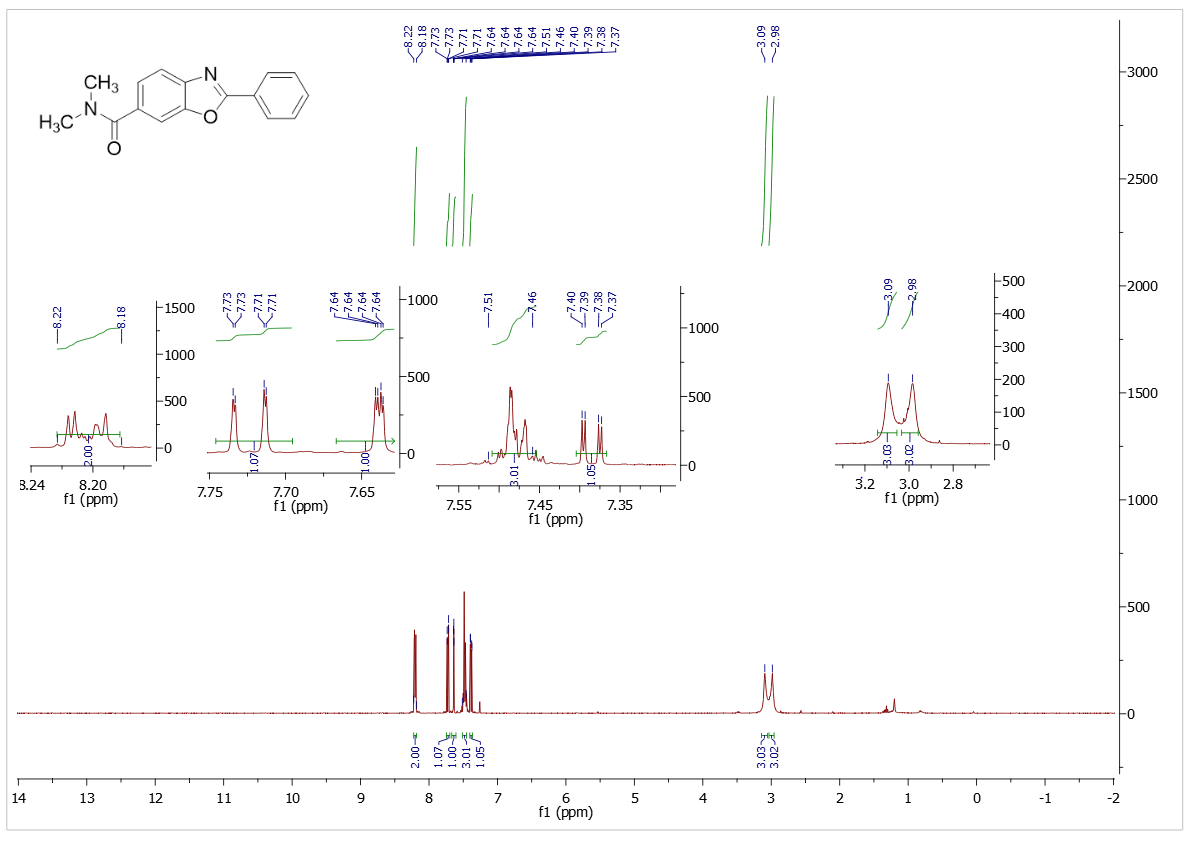


**Figure S1.** ^1^H-NMR Spectrum of compound **7**


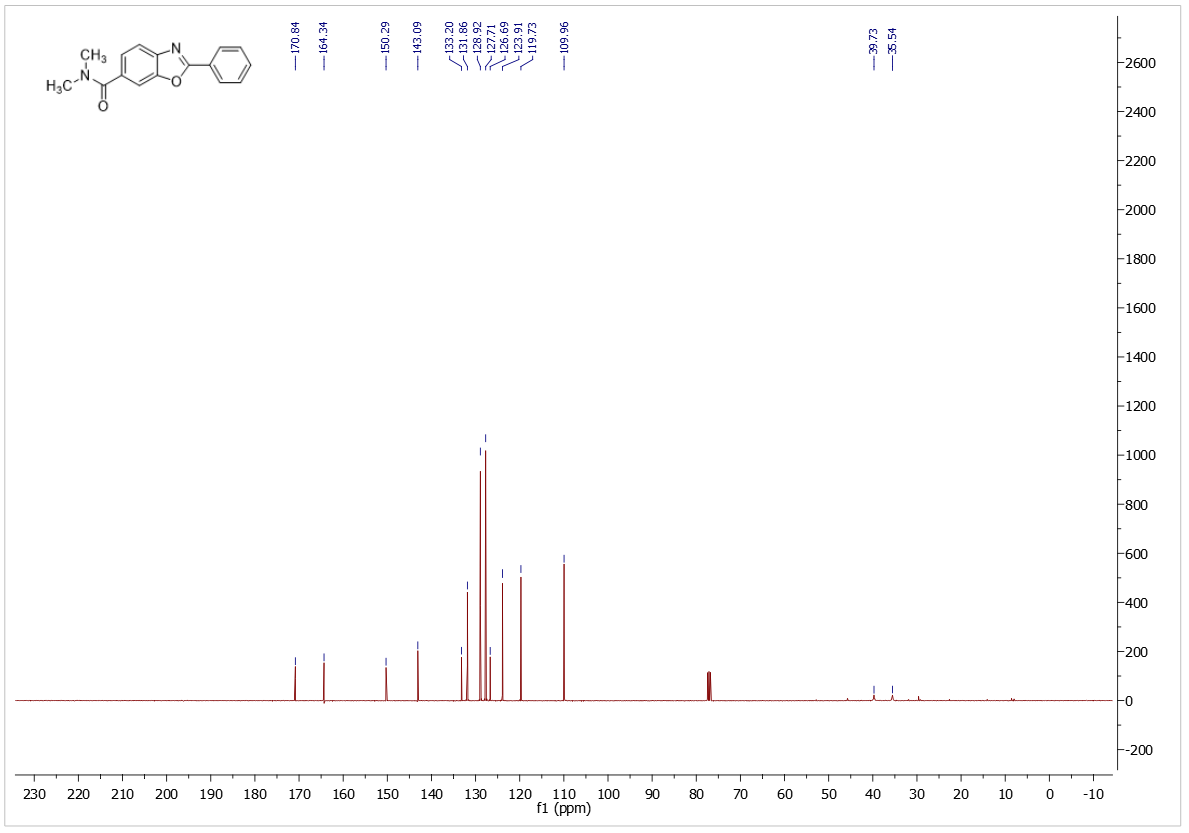


**Figure S2.** ^13^C-NMR Spectrum of compound **7**


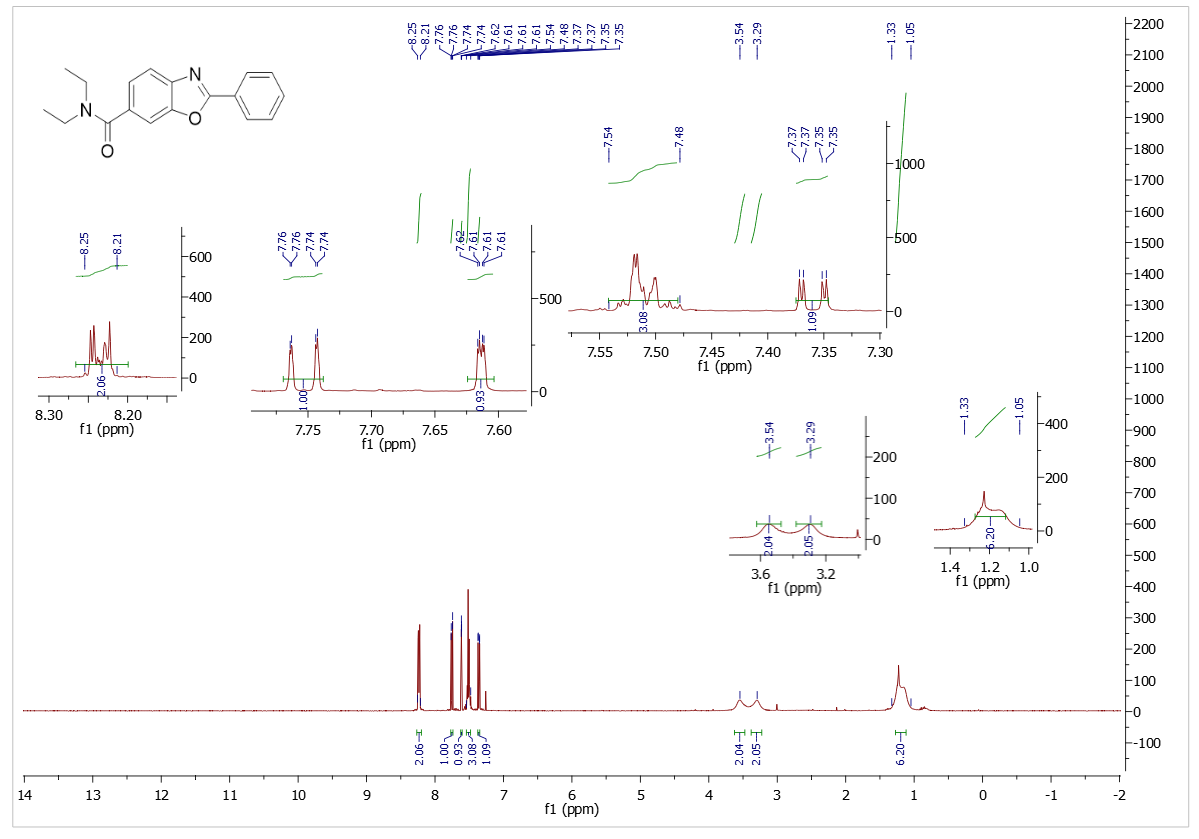


**Figure S3.** ^1^H-NMR Spectrum of compound **8**


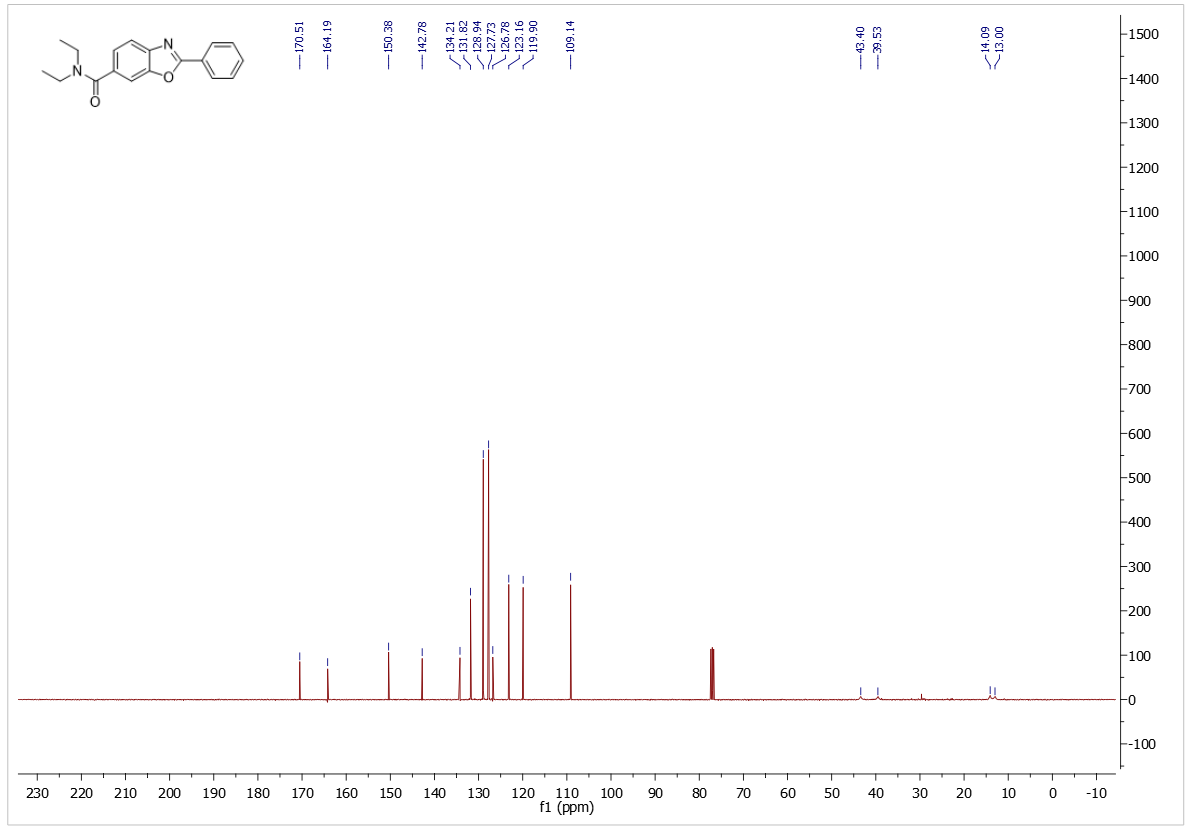


**Figure S4.** ^13^C-NMR Spectrum of compound **8**


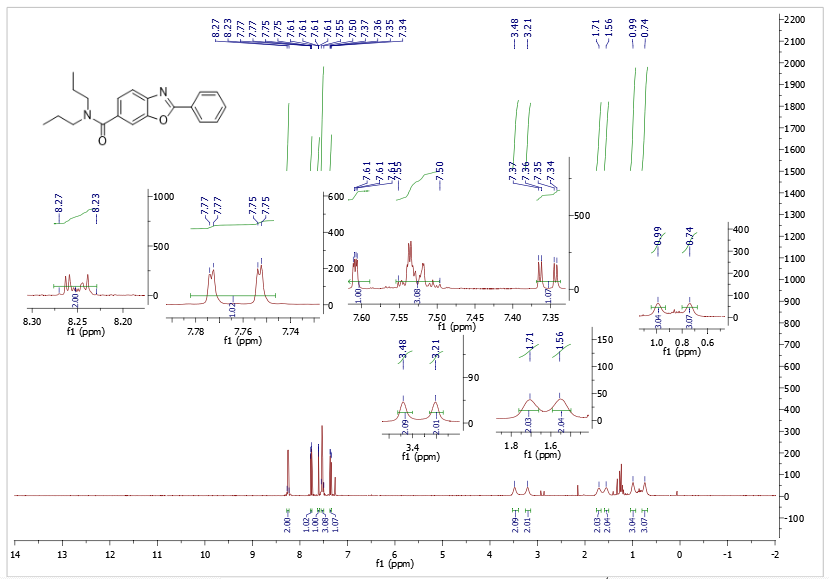


**Figure S5.** ^1^H-NMR Spectrum of compound **9**


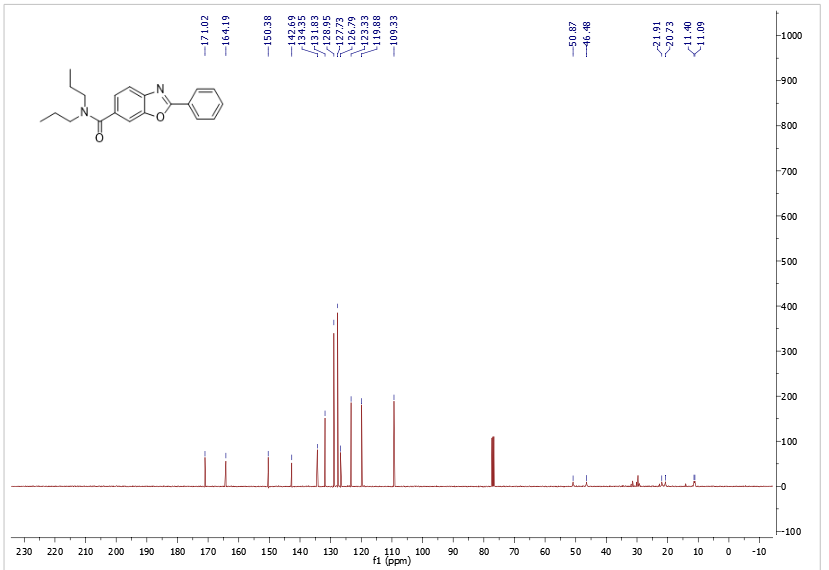


**Figure S6.** ^13^C-NMR Spectrum of compound **9**


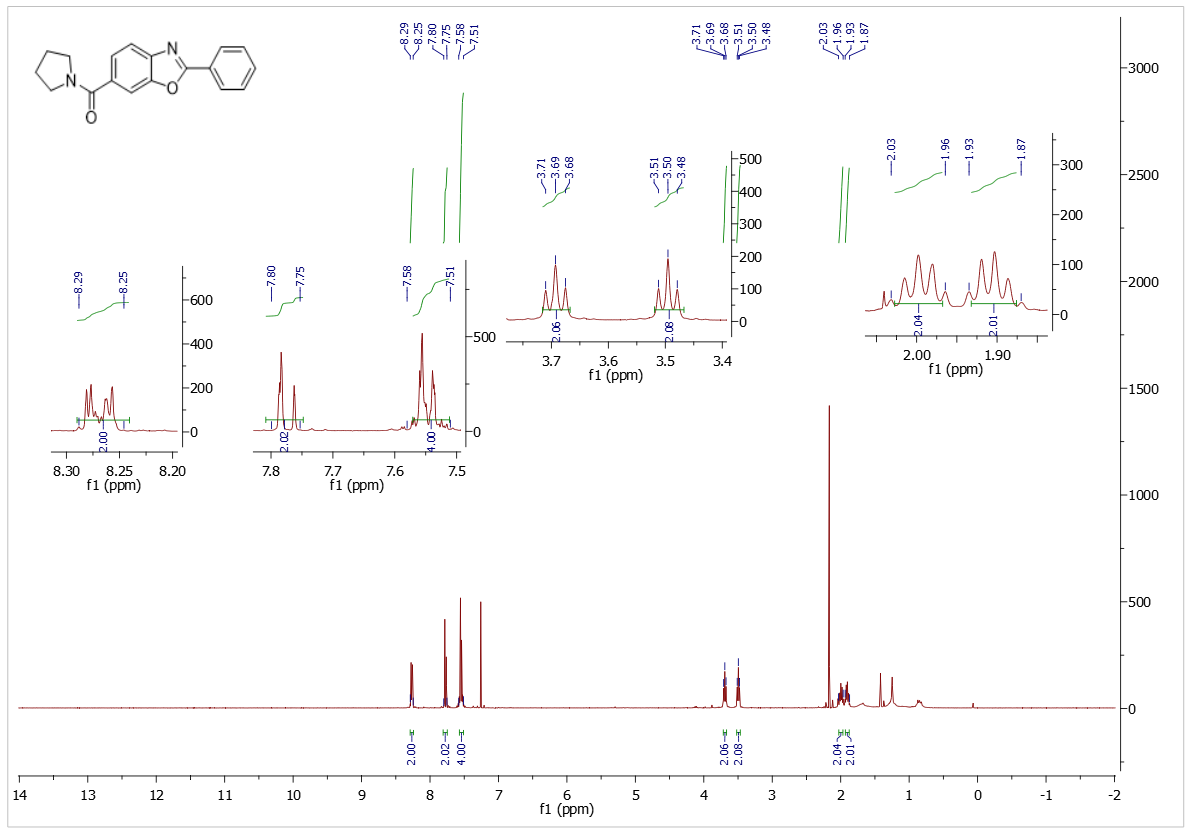


**Figure S7.** ^1^H-NMR Spectrum of compound **10**


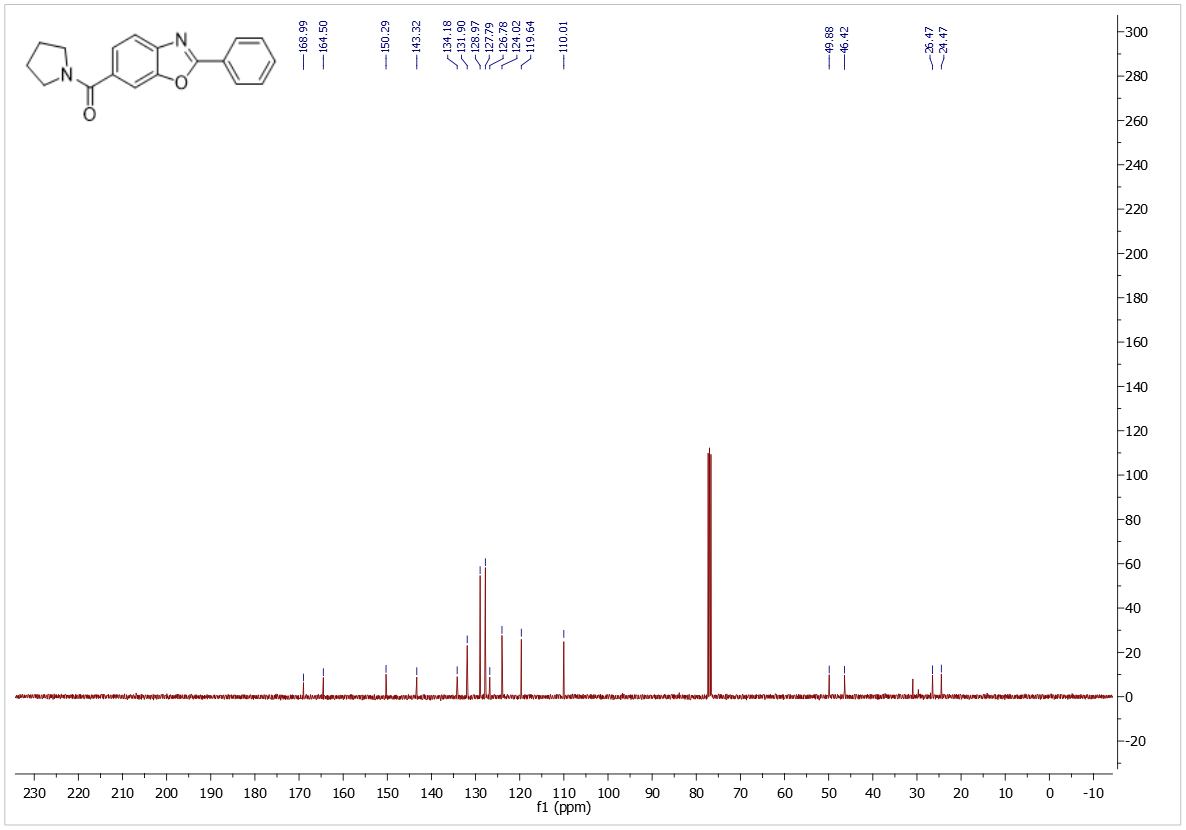


**Figure S8.** ^13^C-NMR Spectrum of compound **10**

**
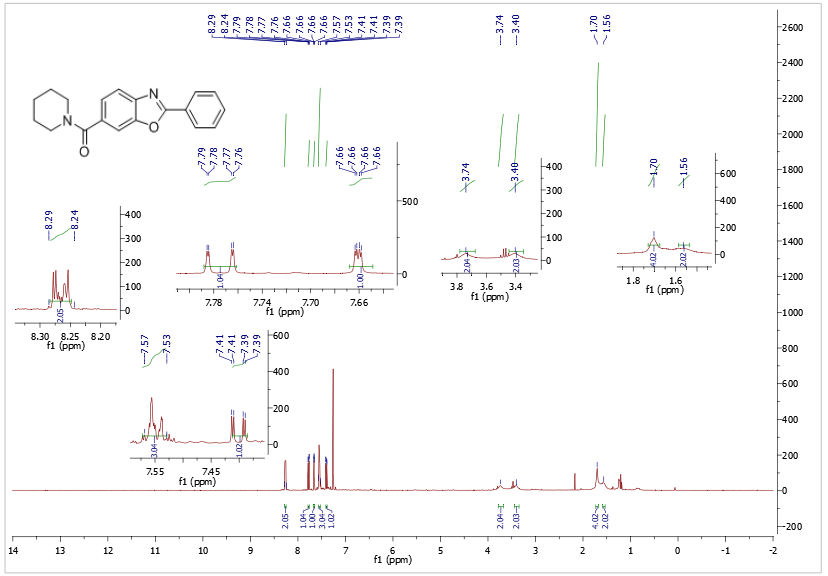
**

**Figure S9.** ^1^H-NMR Spectrum of compound **11**


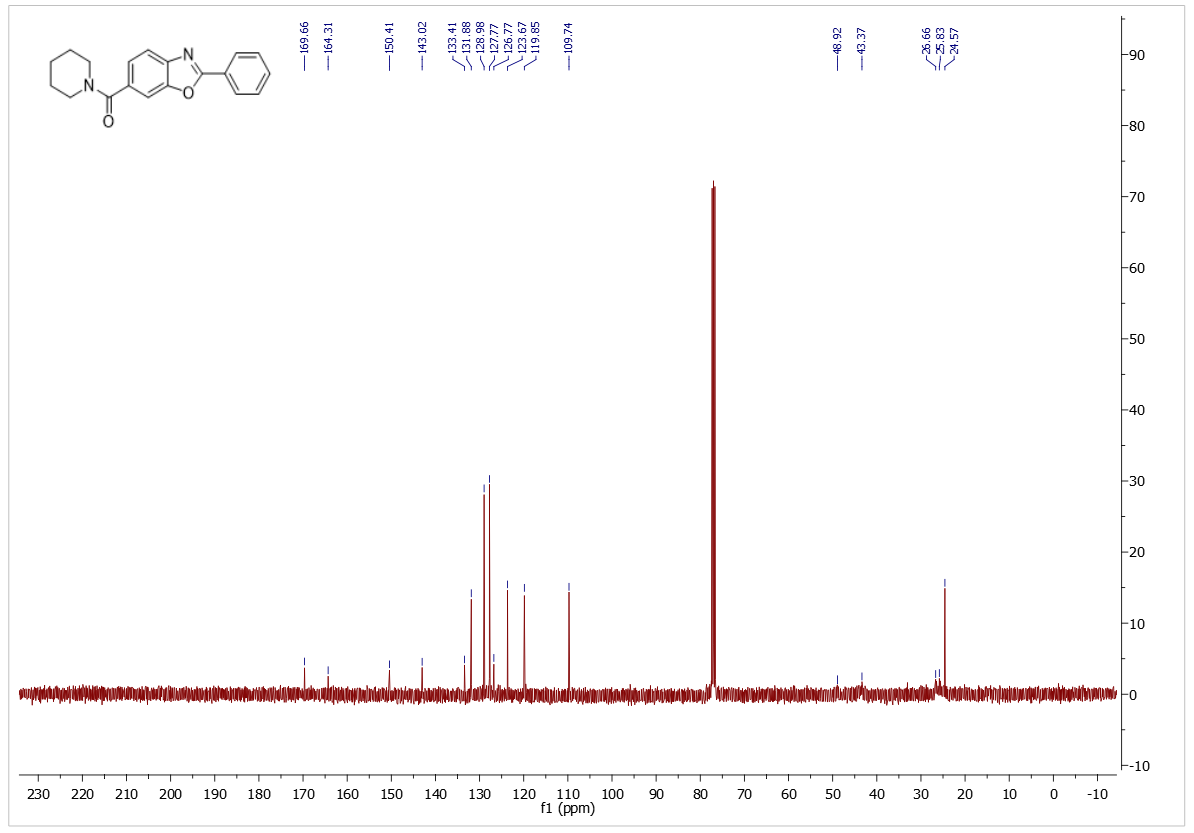


**Figure S10.** ^13^C-NMR Spectrum of compound **11**


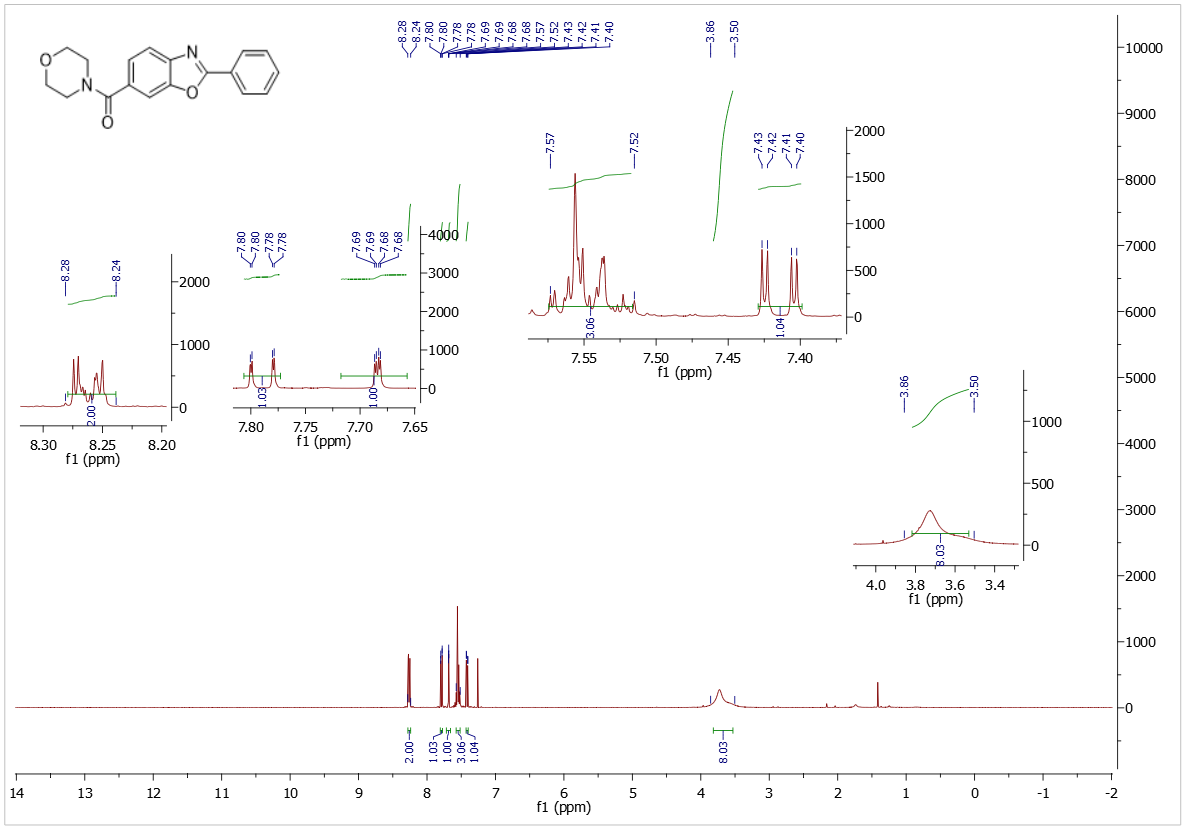


**Figure S11.** ^1^H-NMR Spectrum of compound **12**


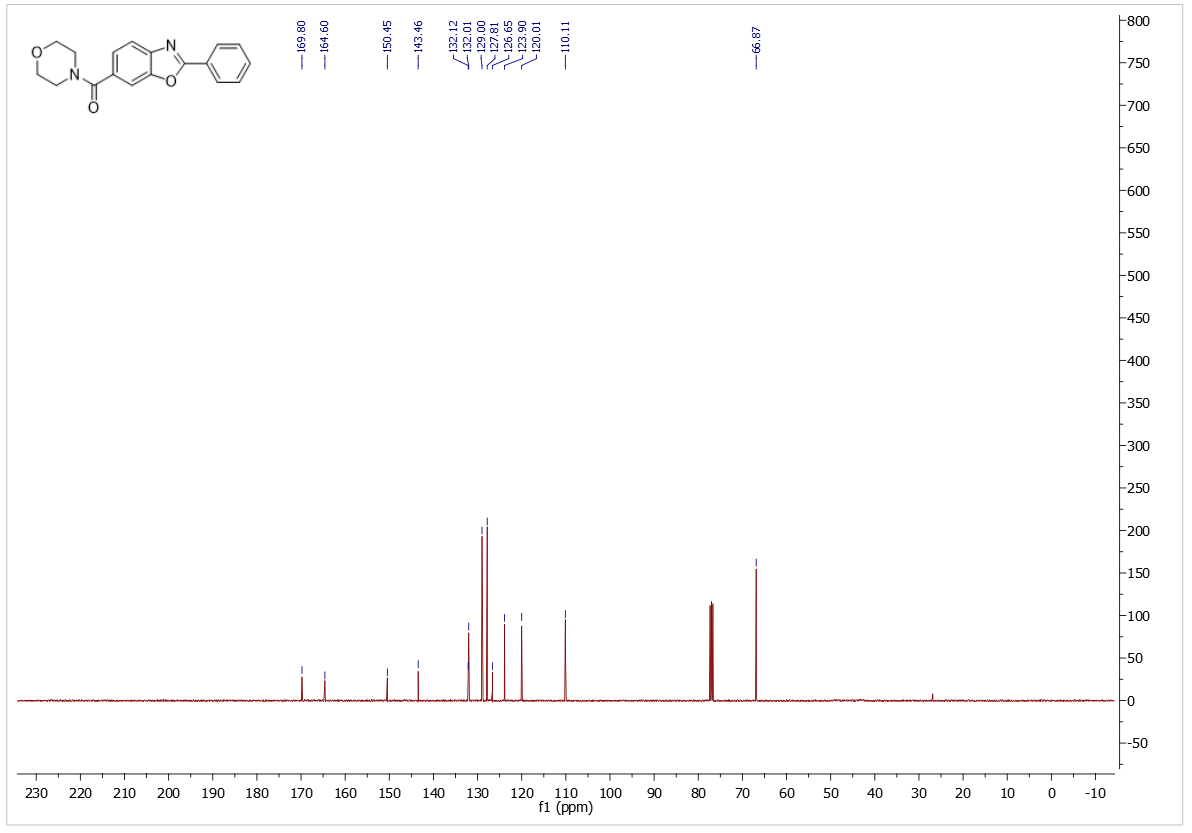


**Figure S12.** ^13^C-NMR Spectrum of compound **12**


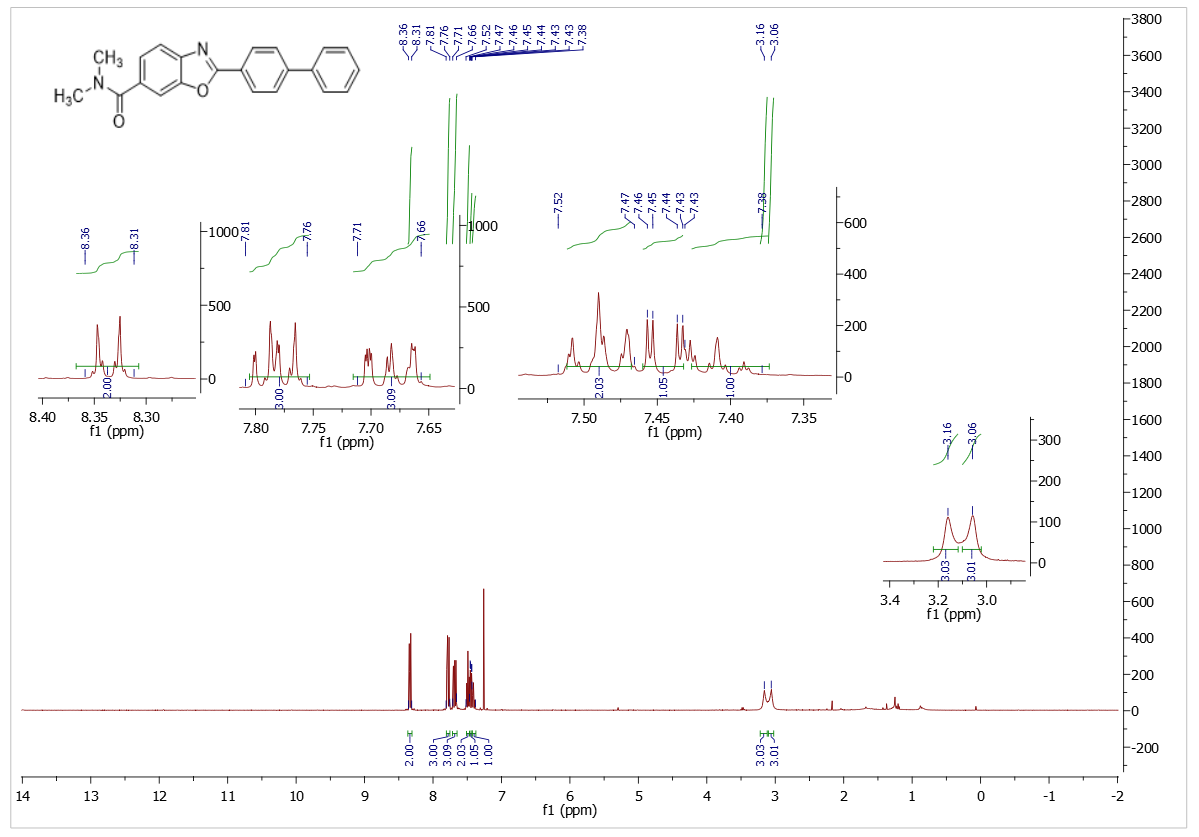


**Figure 13.** ^1^H-NMR Spectrum of compound **13**


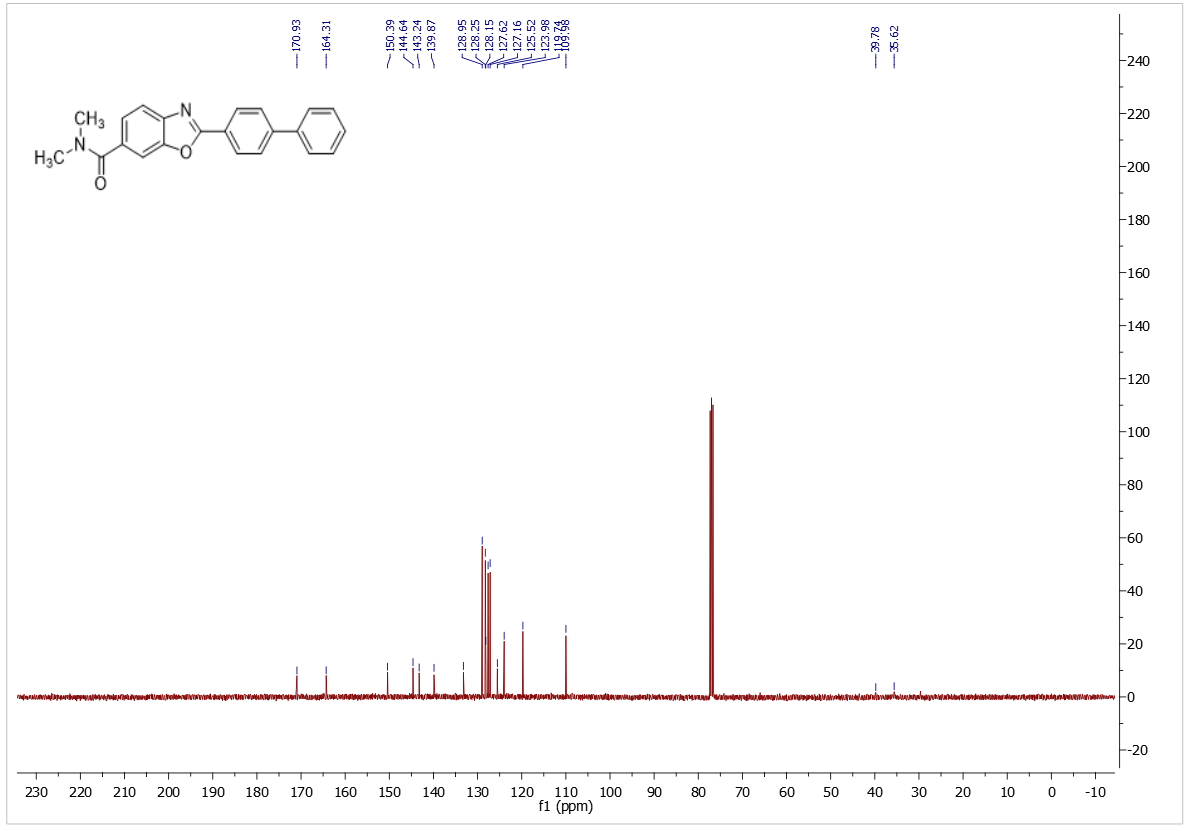


**Figure S14.** ^13^C-NMR Spectrum of compound **13**


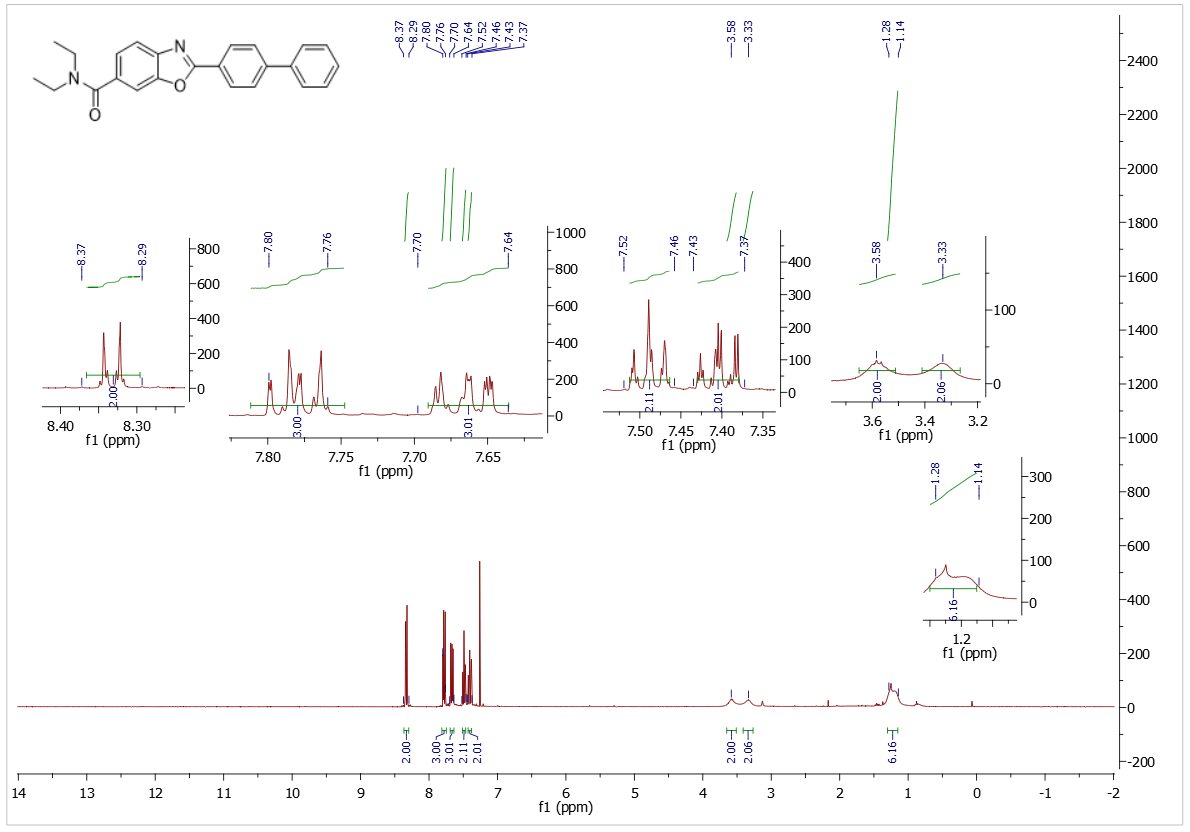


**Figure S15.** ^1^H-NMR Spectrum of compound **14**


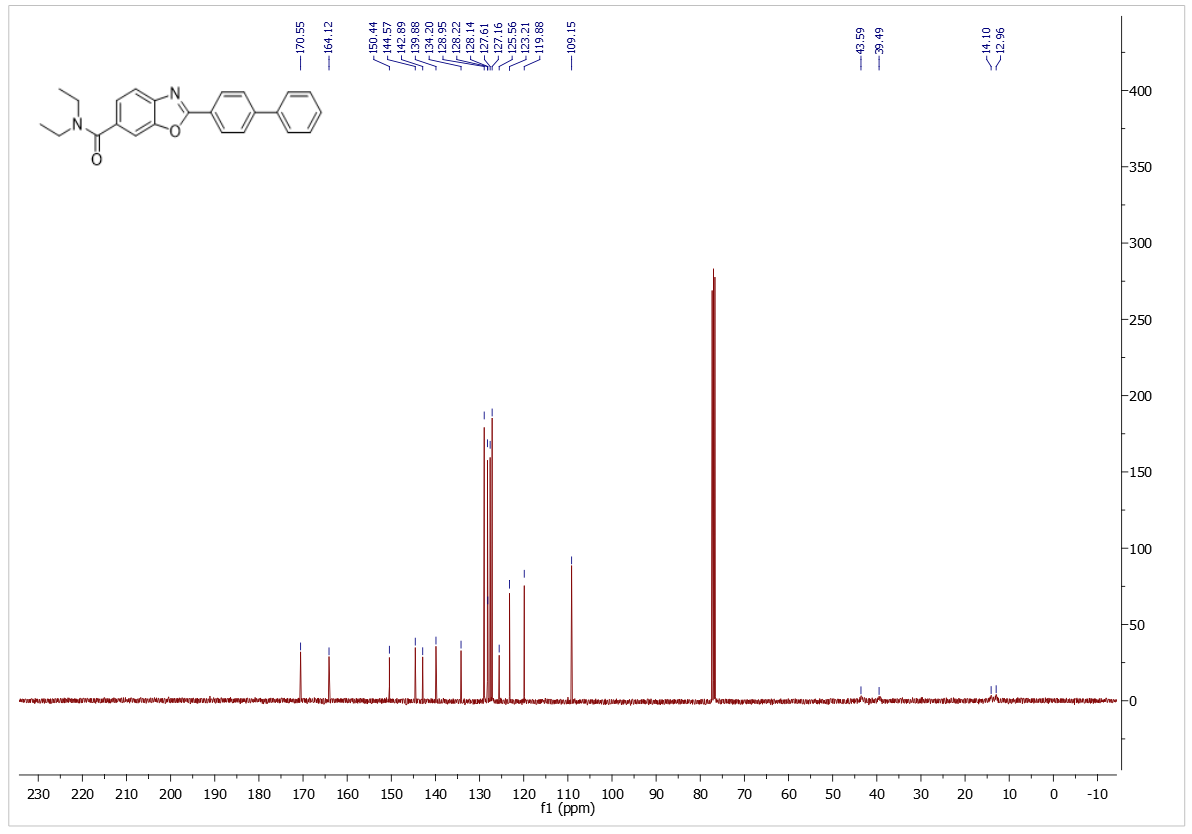


**Figure S16.** ^13^C-NMR Spectrum of compound **14**

**
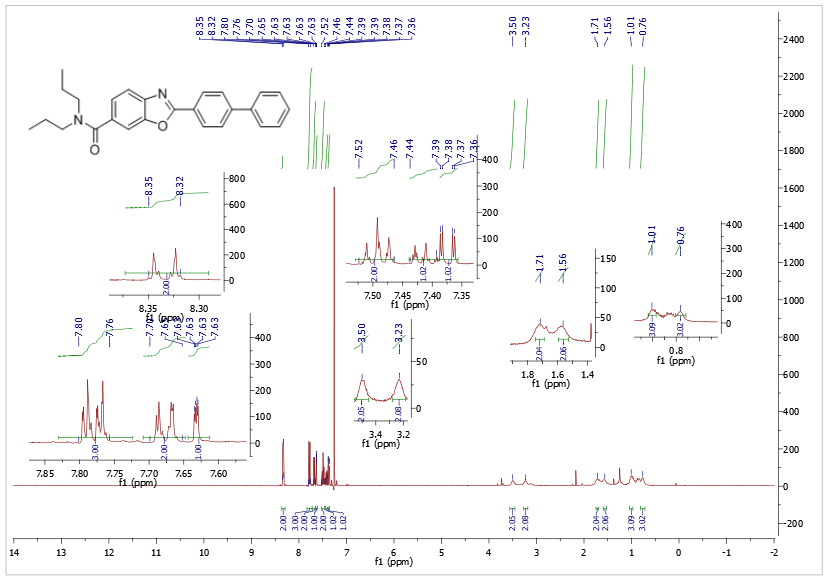
**

**Figure S17.** ^1^H-NMR Spectrum of compound **15**


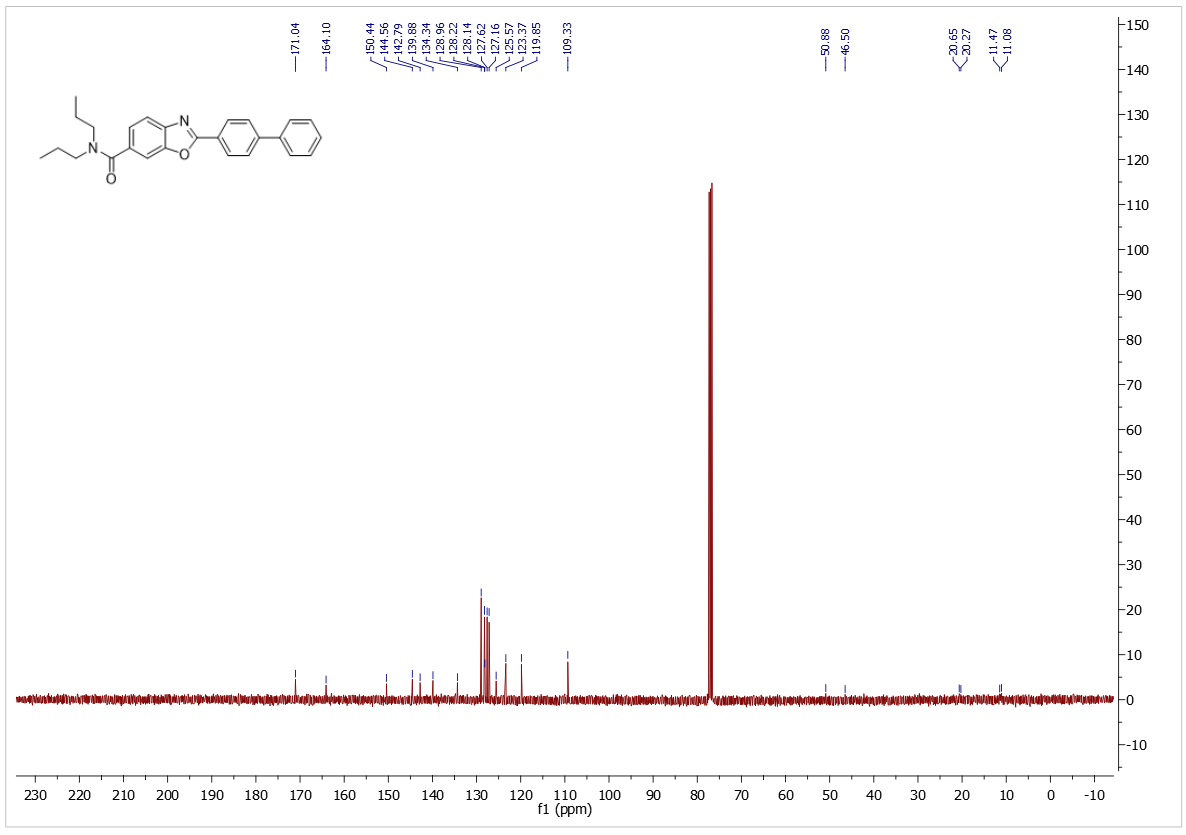


**Figure S18.** ^13^C-NMR Spectrum of compound **15**


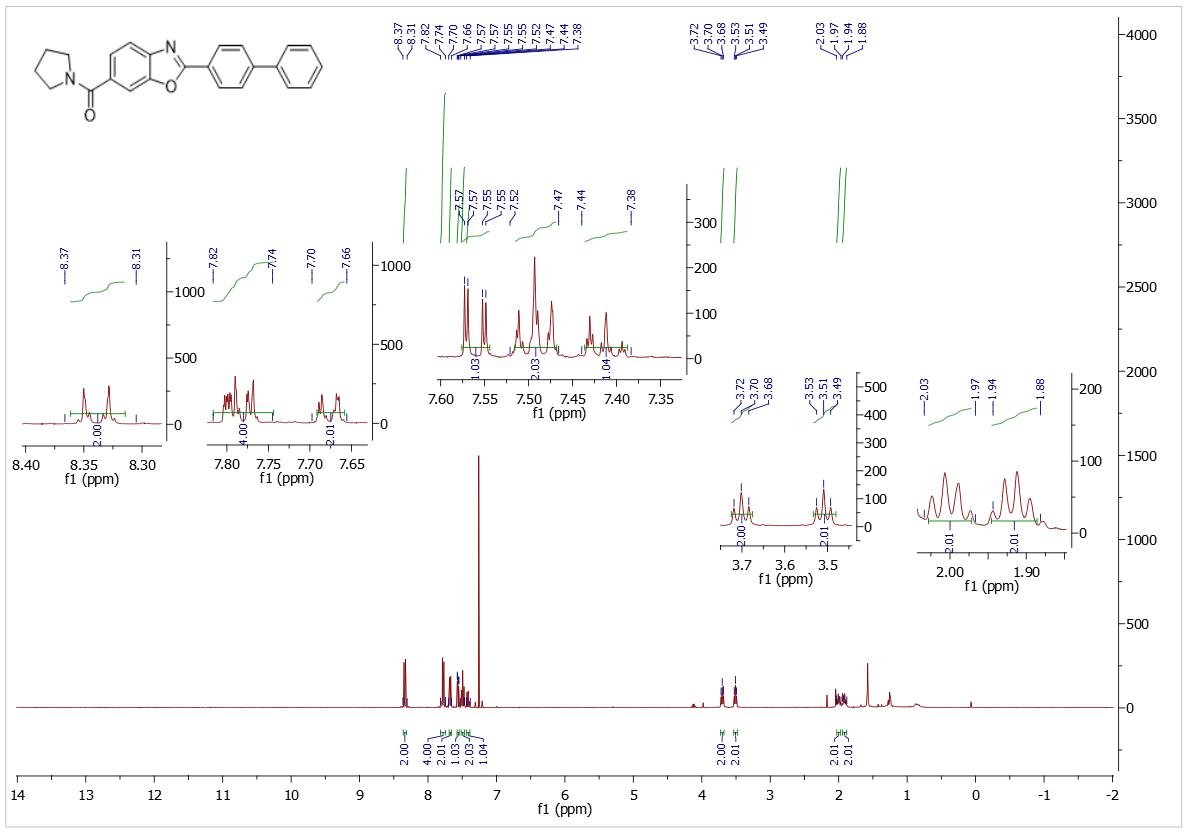


**Figure S19.** ^1^H-NMR Spectrum of compound **16**


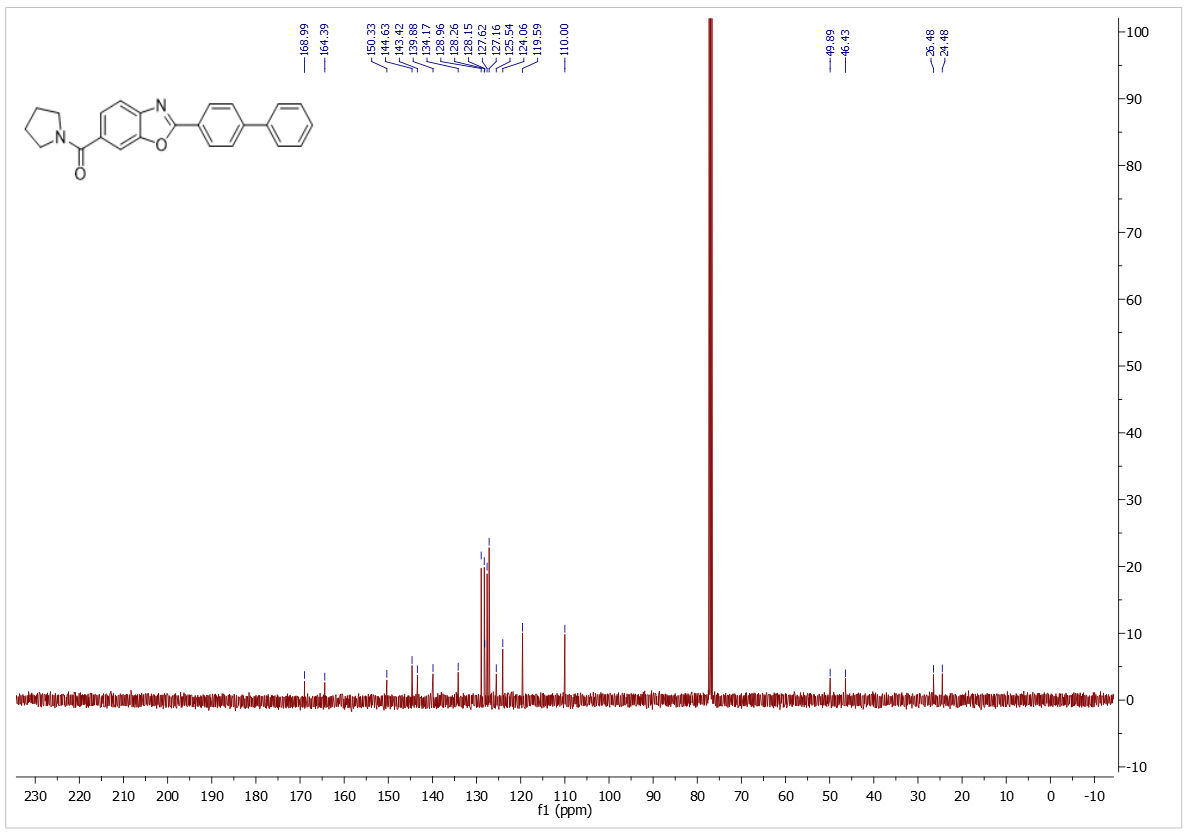


**Figure S20.** ^13^C-NMR Spectrum of compound **16**


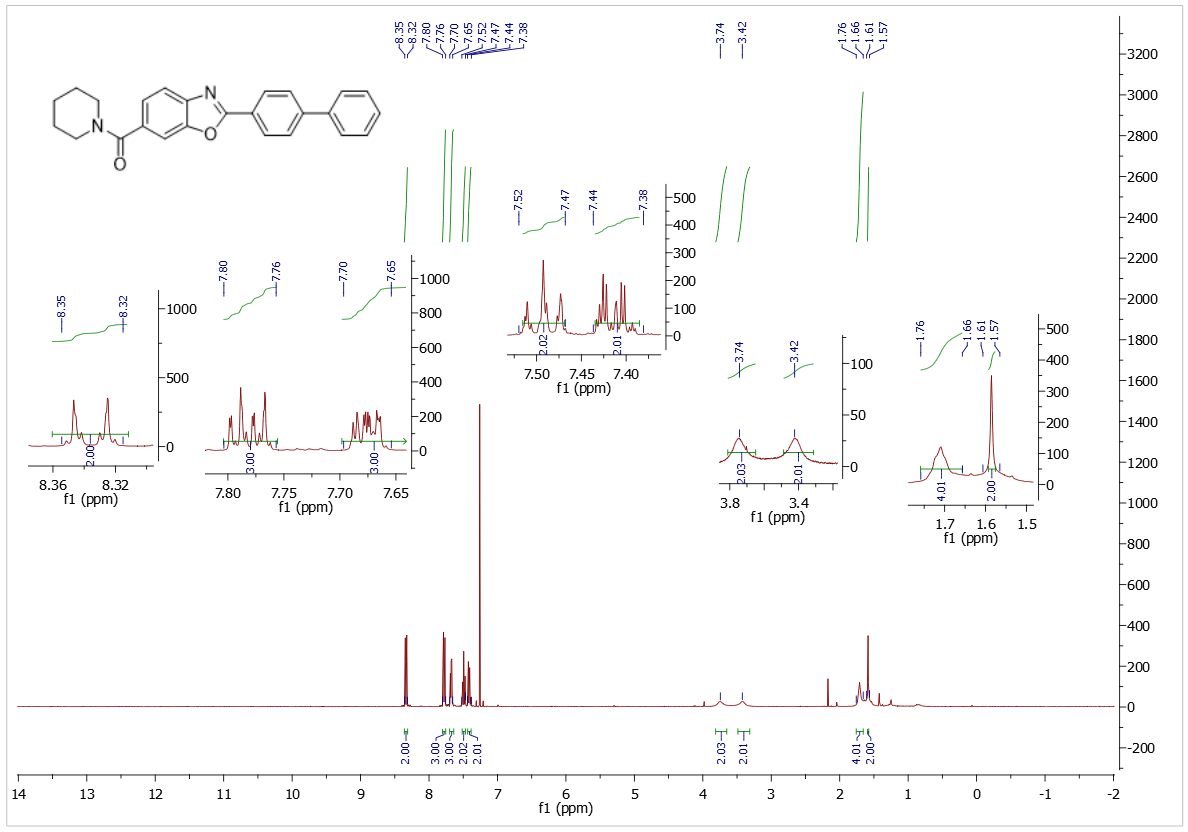


**Figure S21.** ^1^H-NMR Spectrum of compound **17**


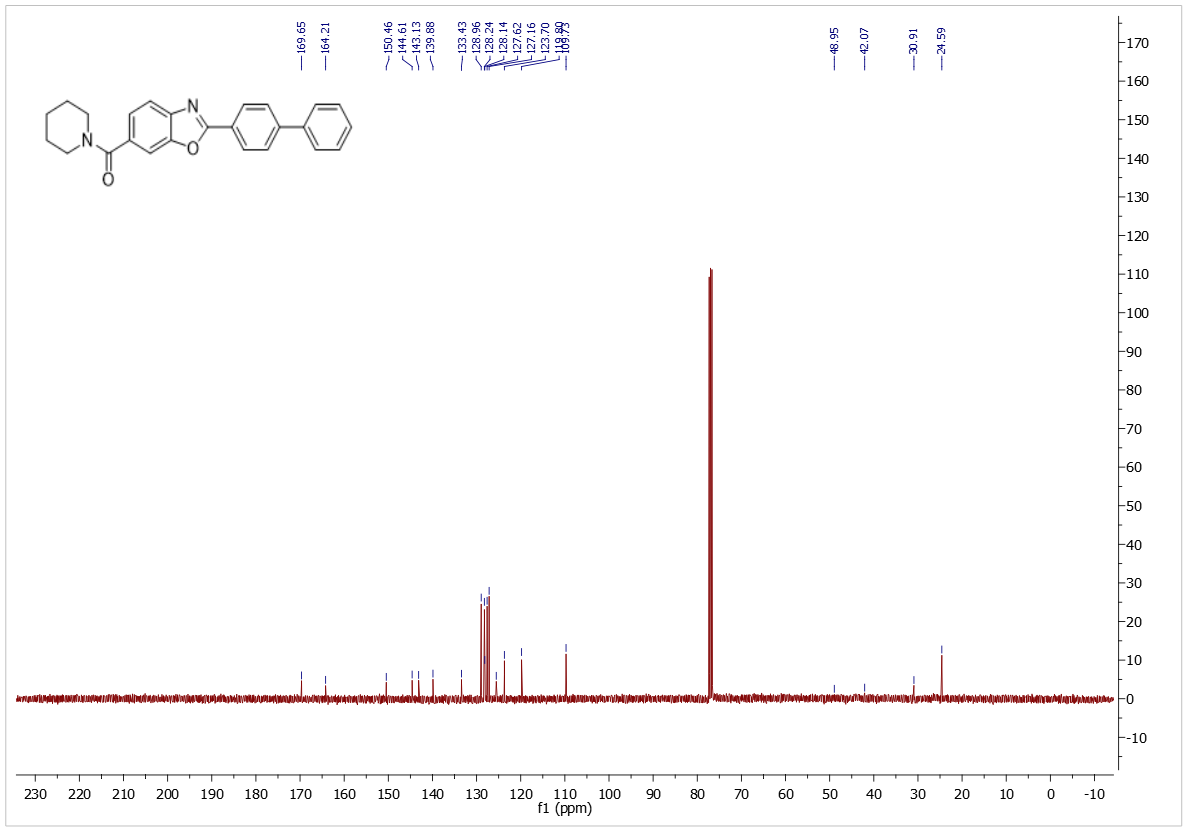


**Figure S22.** ^13^C-NMR Spectrum of compound **17**


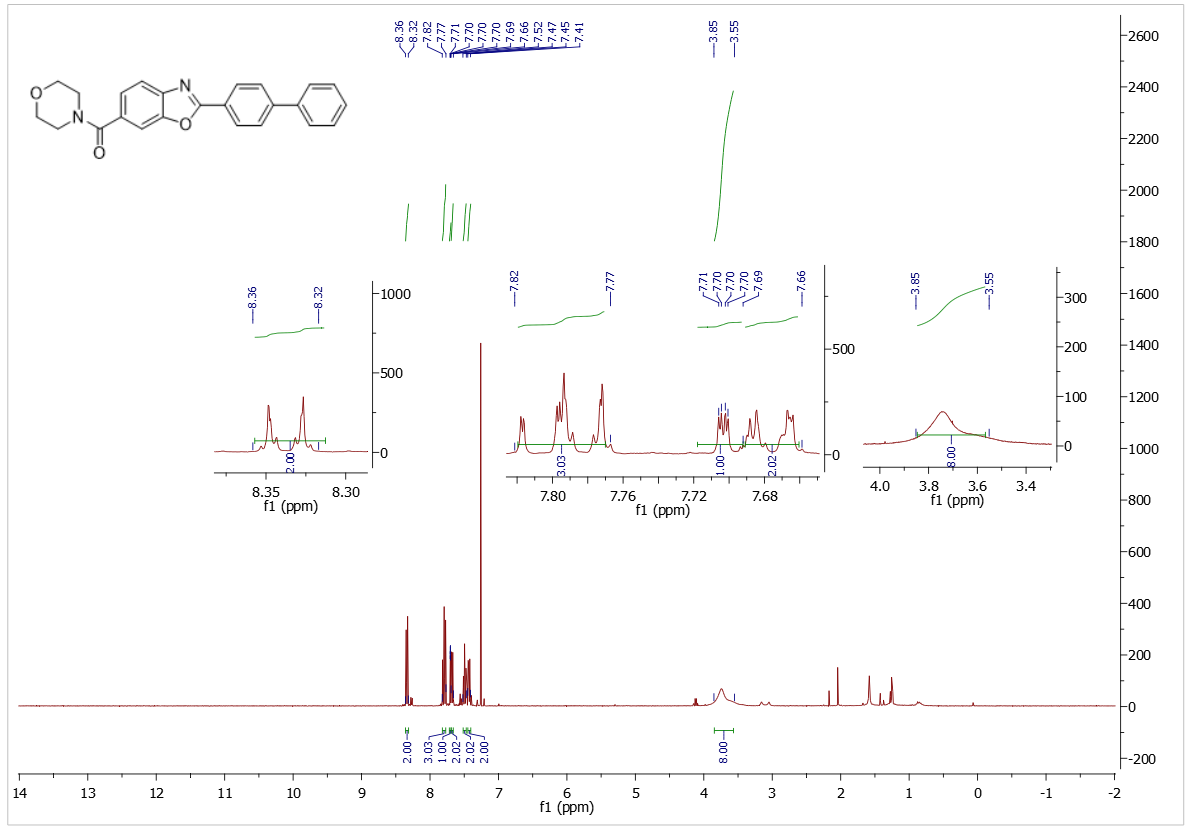


**Figure S23.** ^1^H-NMR Spectrum of compound **18**


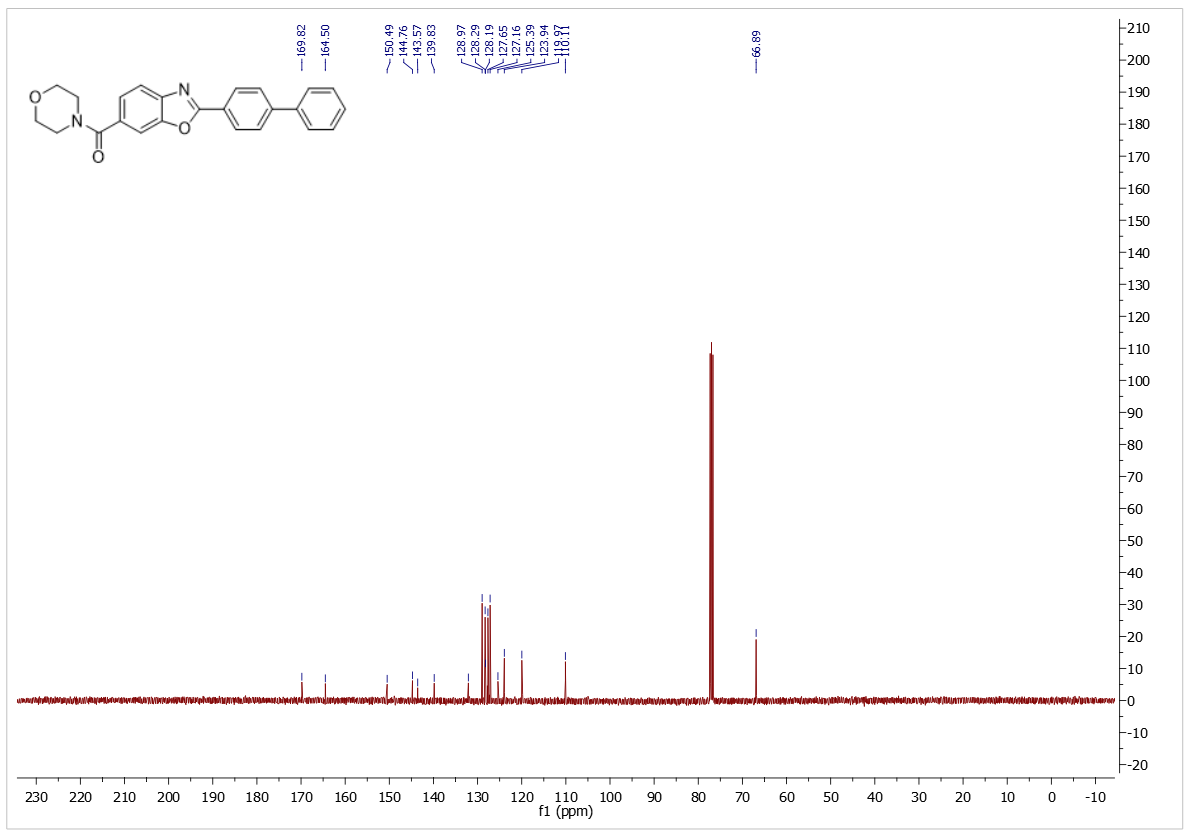


**Figure S24.** ^13^C-NMR Spectrum of compound **18**


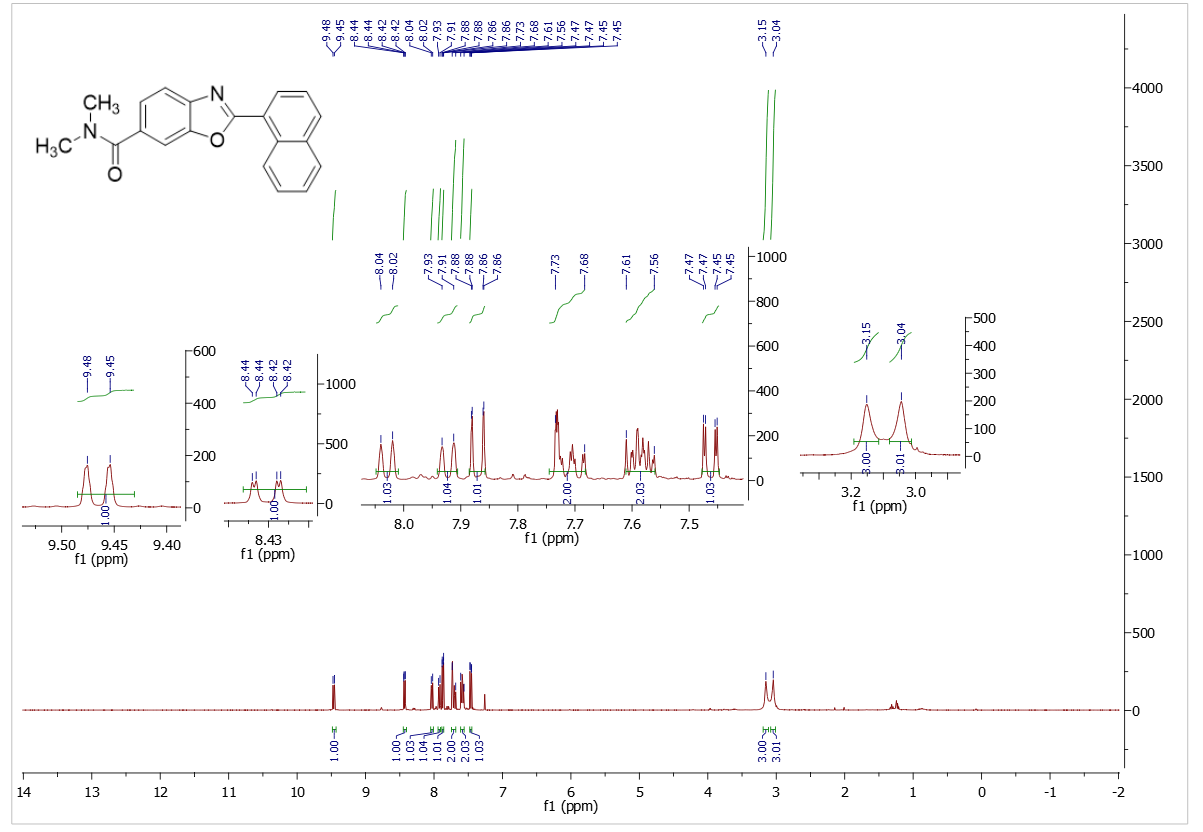


**Figure S25.** ^1^H-NMR Spectrum of compound **19**


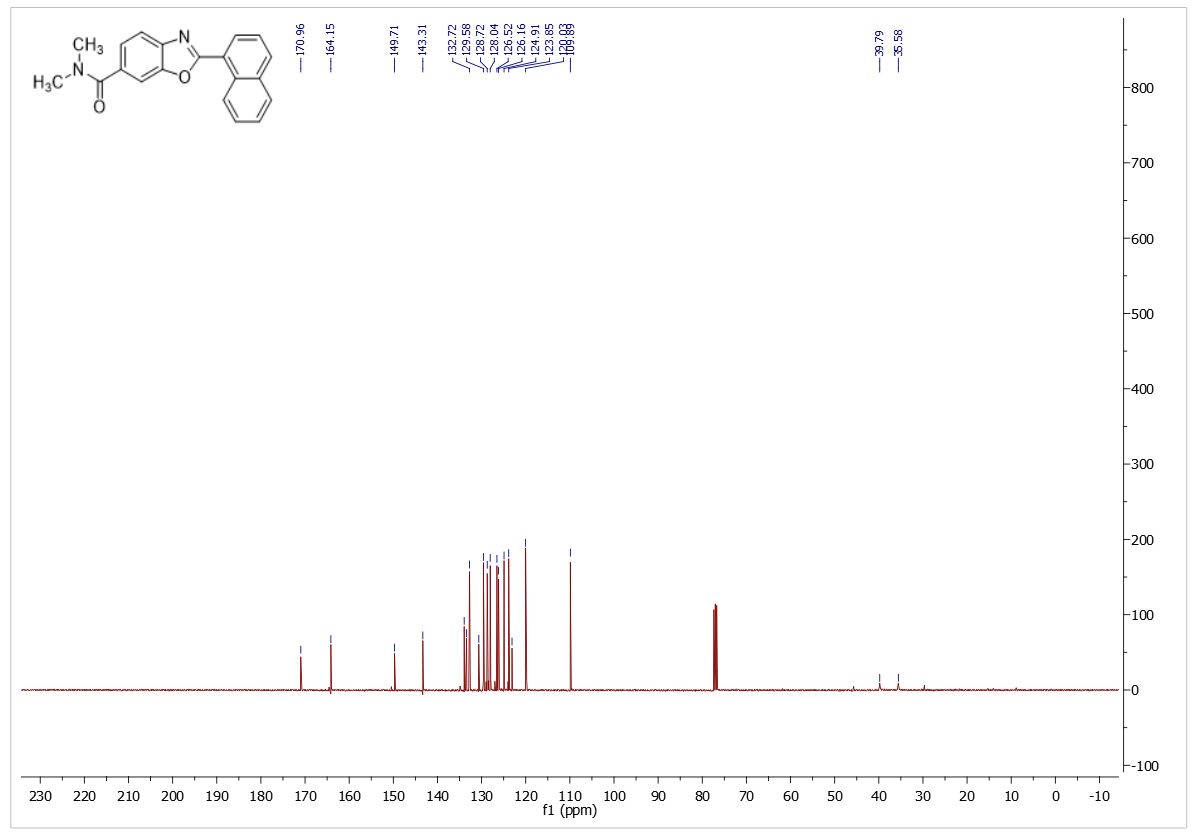


**Figure S26.** ^13^C-NMR Spectrum of compound **19**


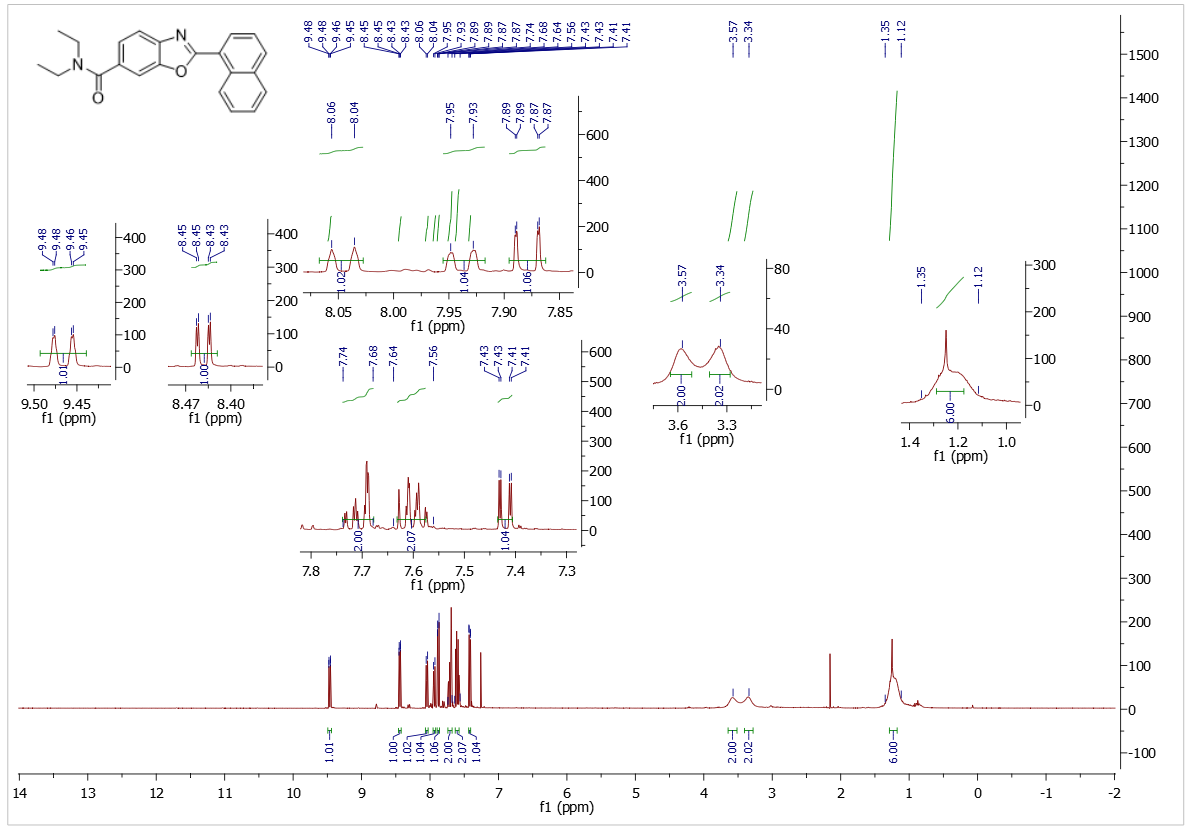


**Figure S27.** ^1^H-NMR Spectrum of compound **20**


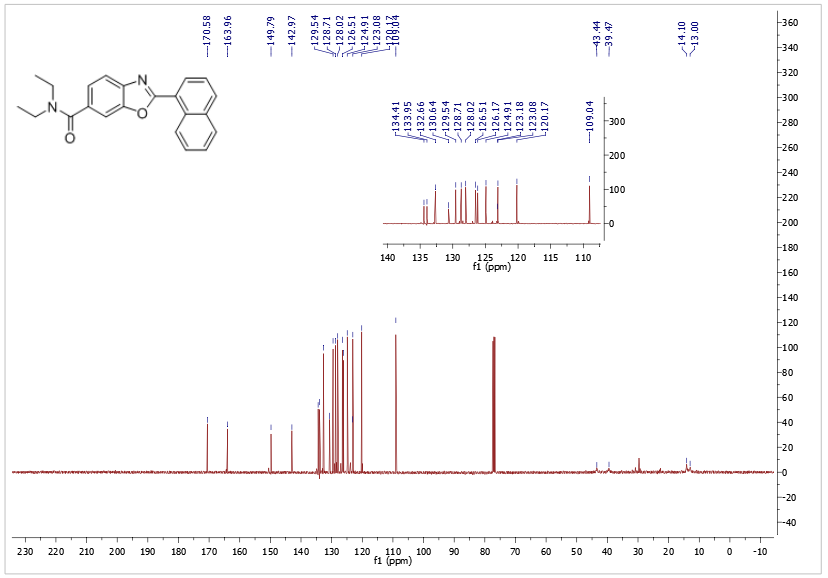


**Figure S28.** ^13^C-NMR Spectrum of compound **20**


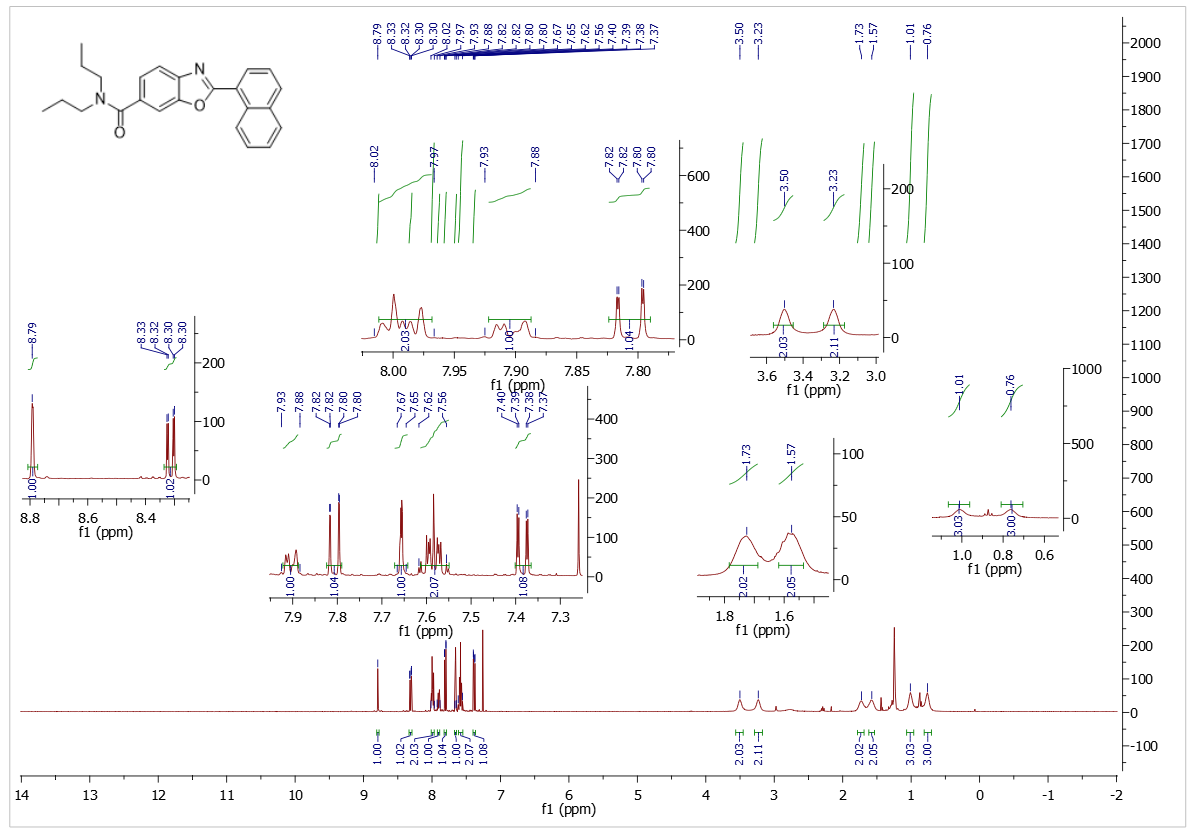


**Figure S29.** ^1^H-NMR Spectrum of compound **21**


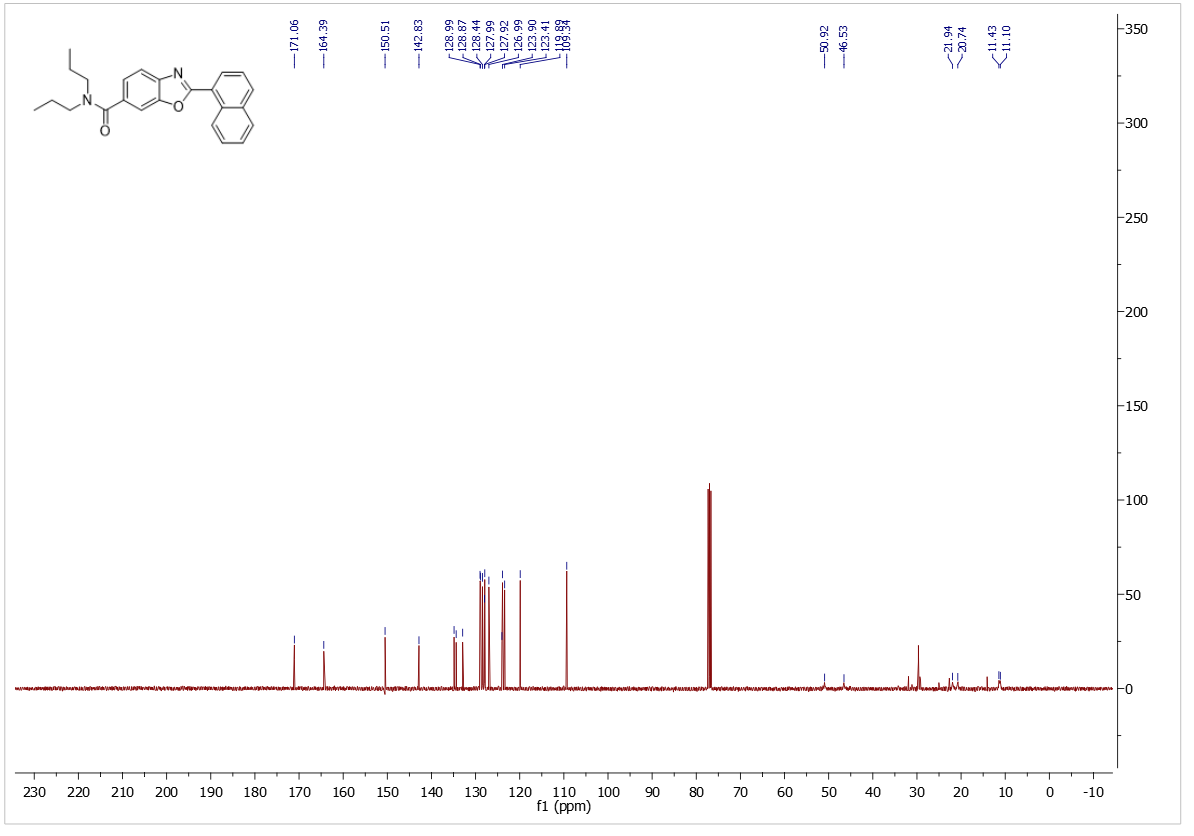


**Figure S30.** ^13^C-NMR Spectrum of compound **21**


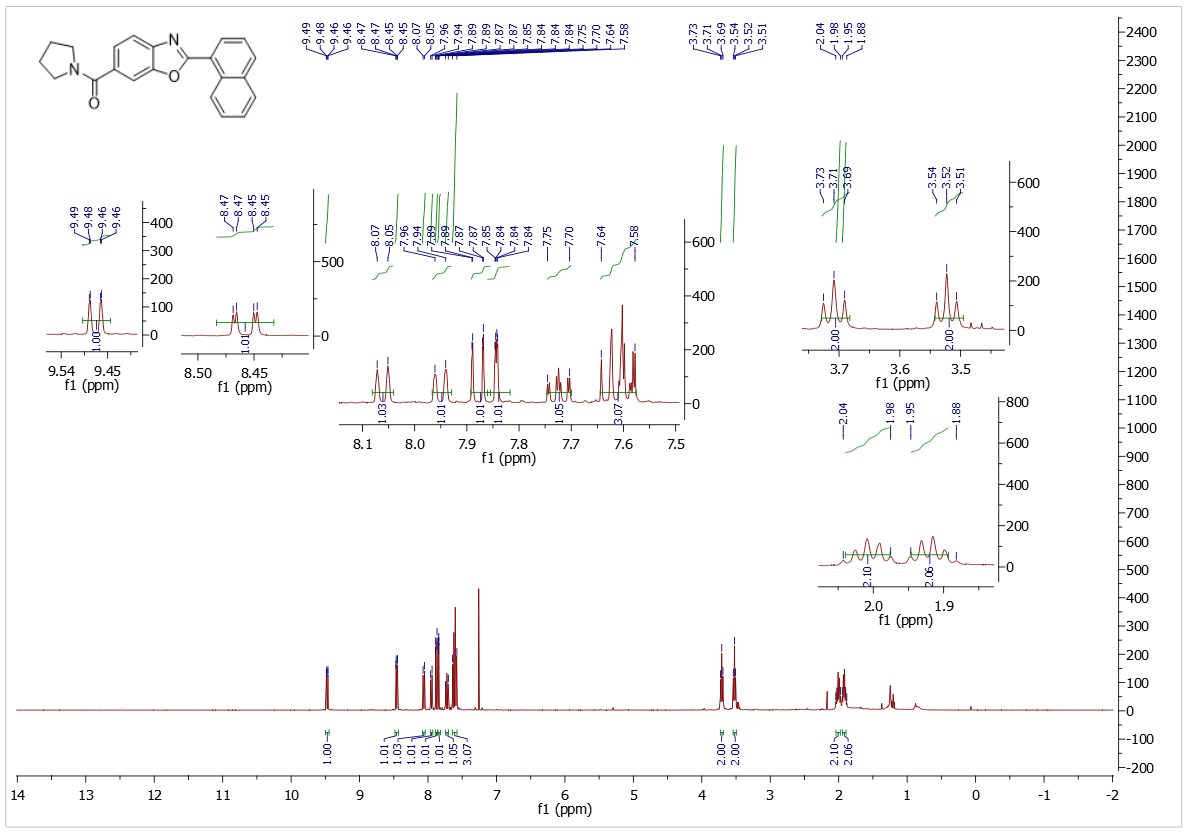


**Figure S31.** ^1^H-NMR Spectrum of compound **22**

**
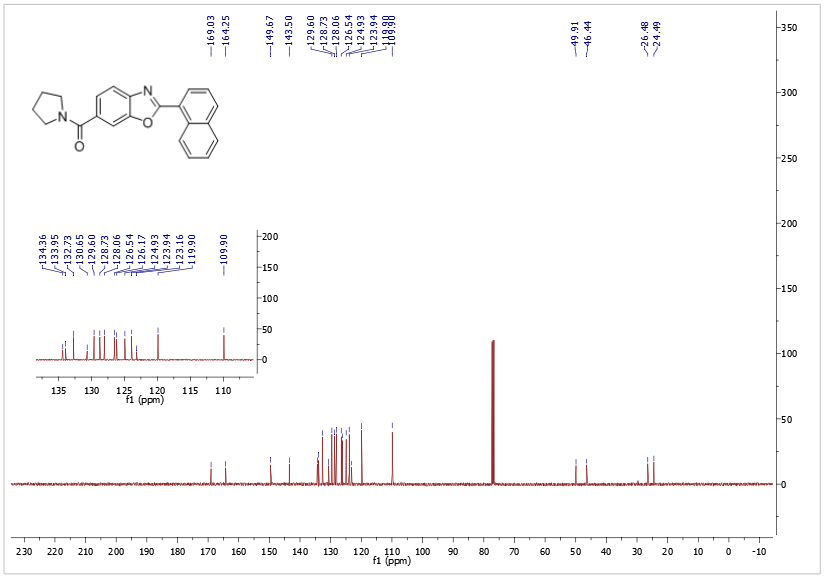
**

**Figure S32.** ^13^C-NMR Spectrum of compound **22**


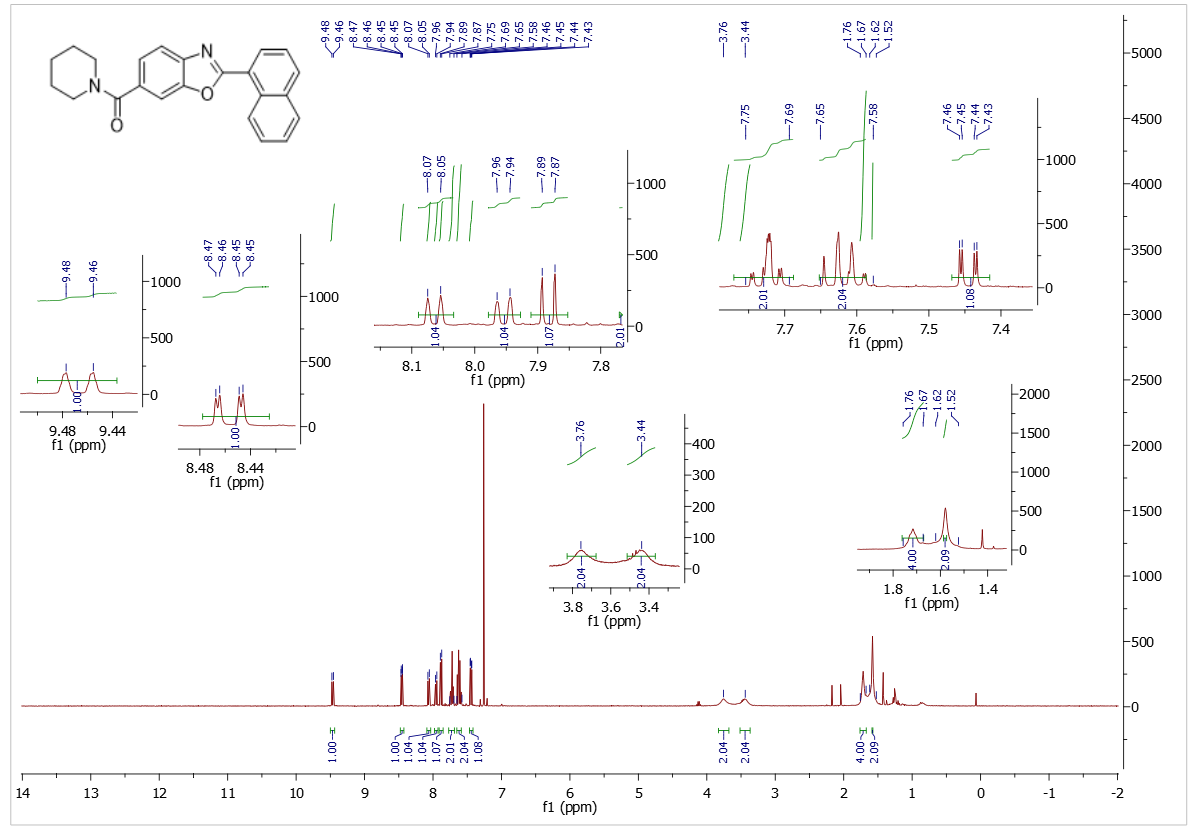


**Figure S33.** ^1^H-NMR Spectrum of compound **23**


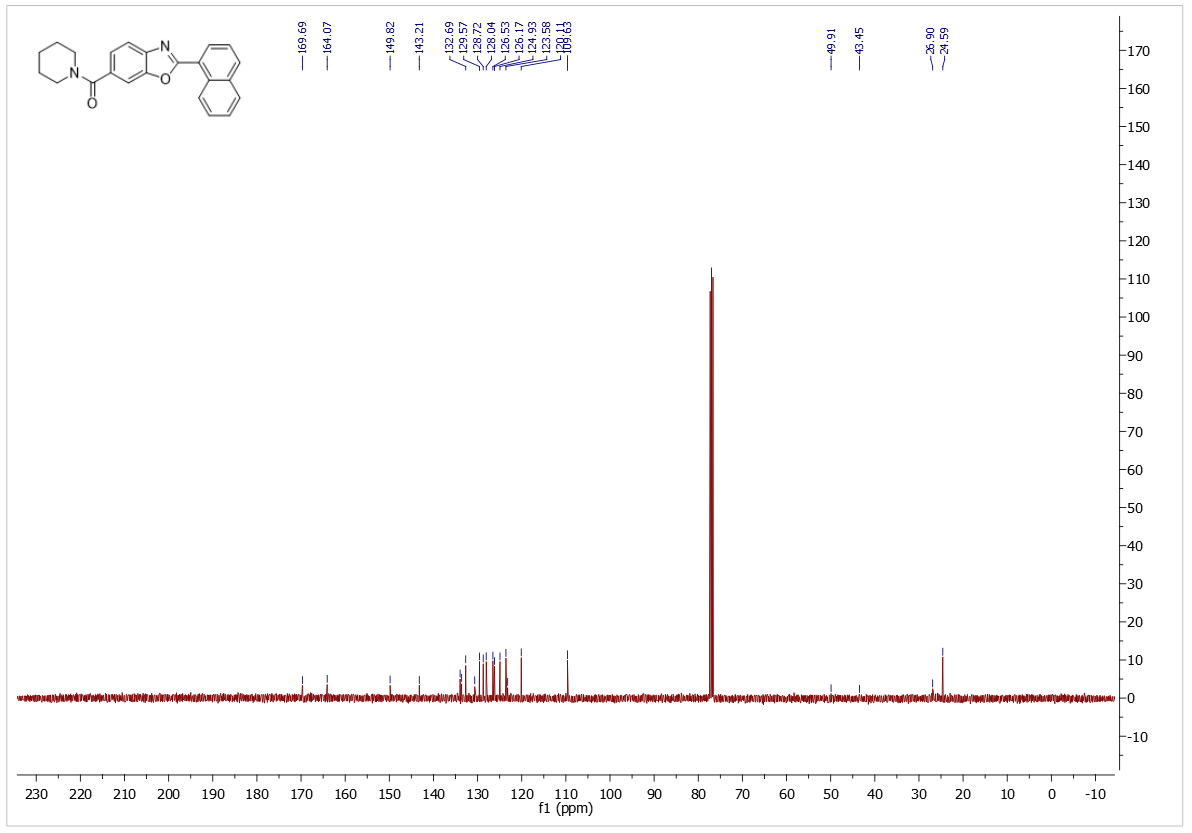


**Figure S34.** ^13^C-NMR Spectrum of compound **23**

**
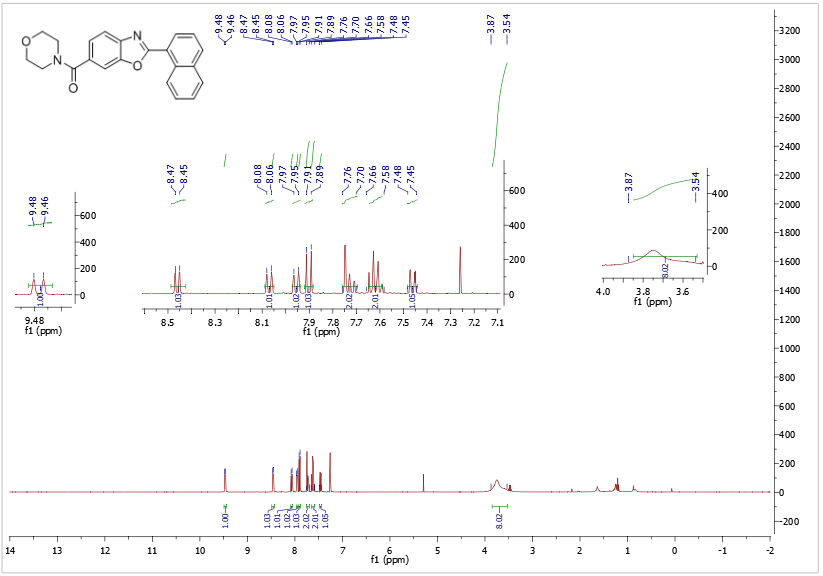
**

**Figure S35.** ^1^H-NMR Spectrum of compound **24**

**
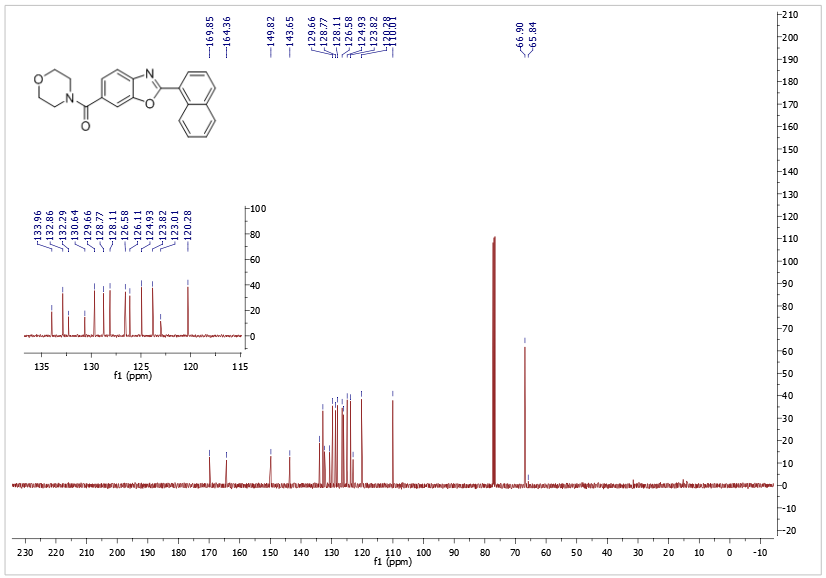
**

**Figure S36.** ^13^C-NMR Spectrum of compound **24**


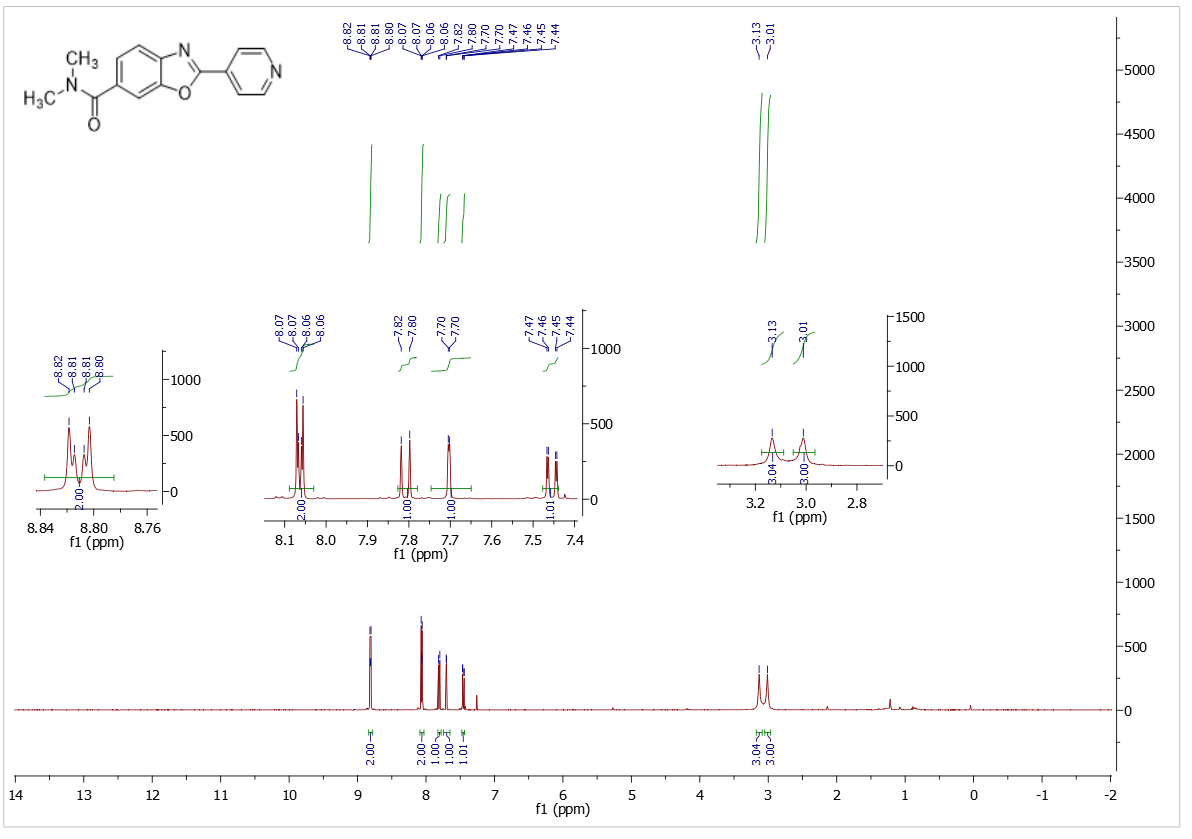


**Figure S37.** ^1^H-NMR Spectrum of compound **25**


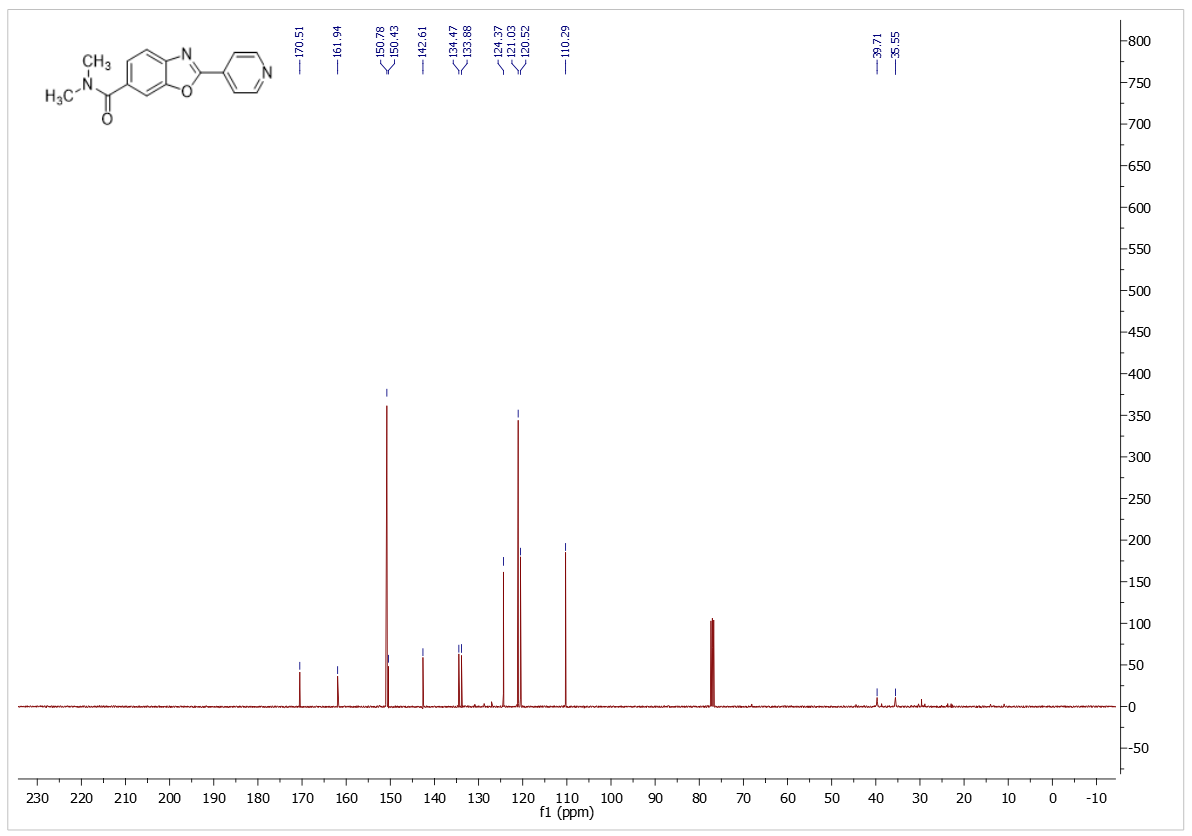


**Figure S38.** ^13^C-NMR Spectrum of compound **25**


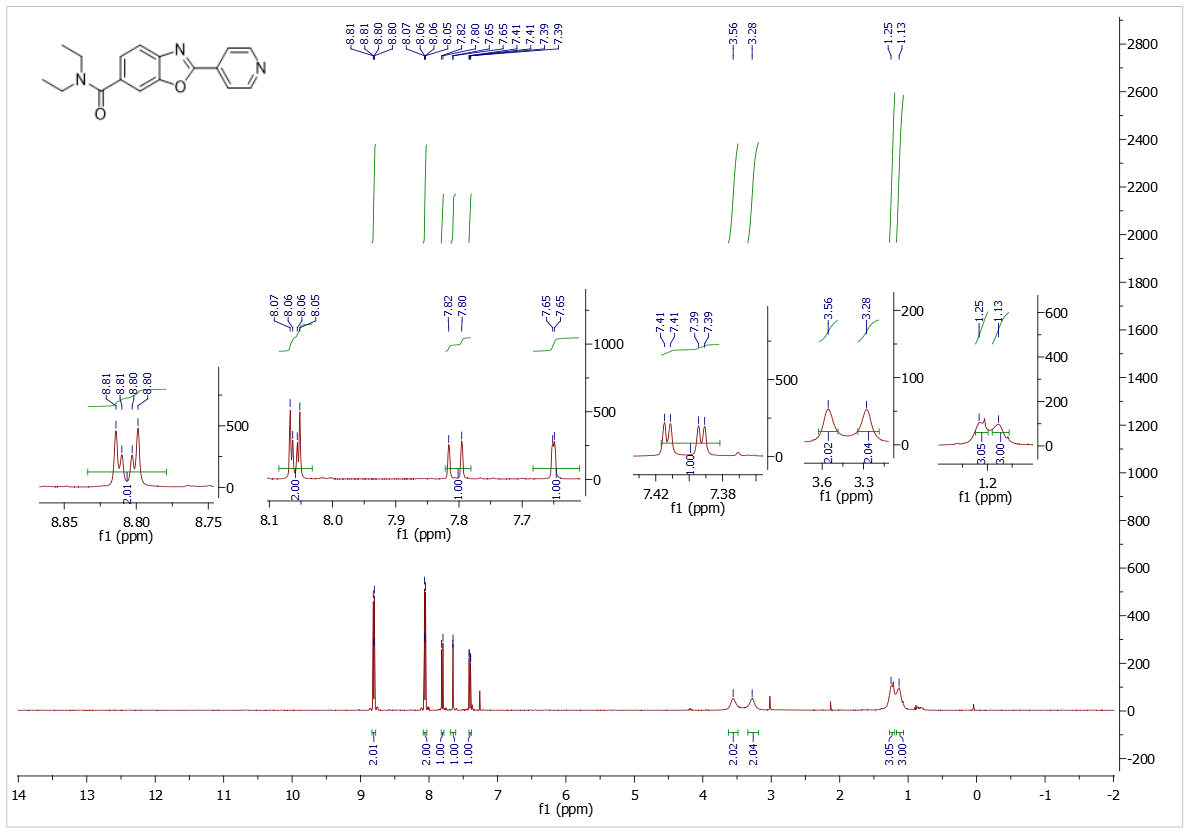


**Figure S39.** ^1^H-NMR Spectrum of compound **26**


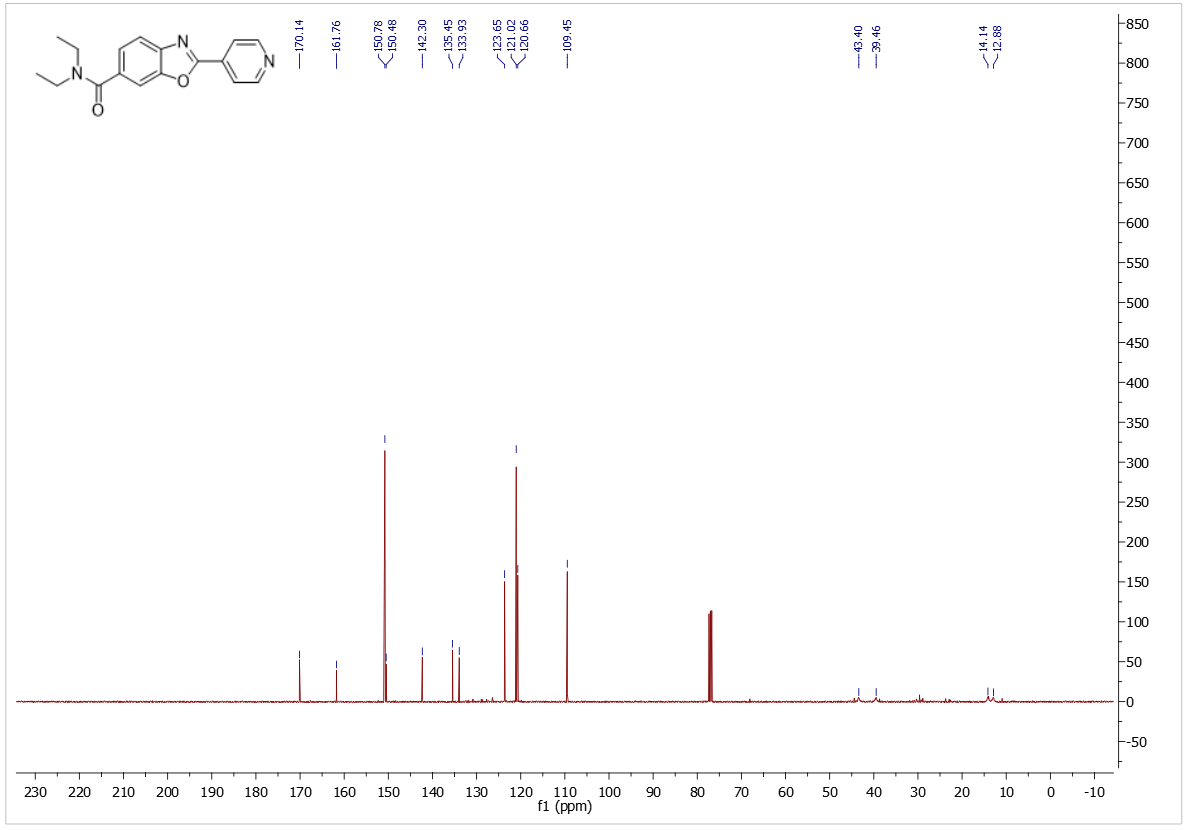


**Figure S40.** ^13^C-NMR Spectrum of compound **26**


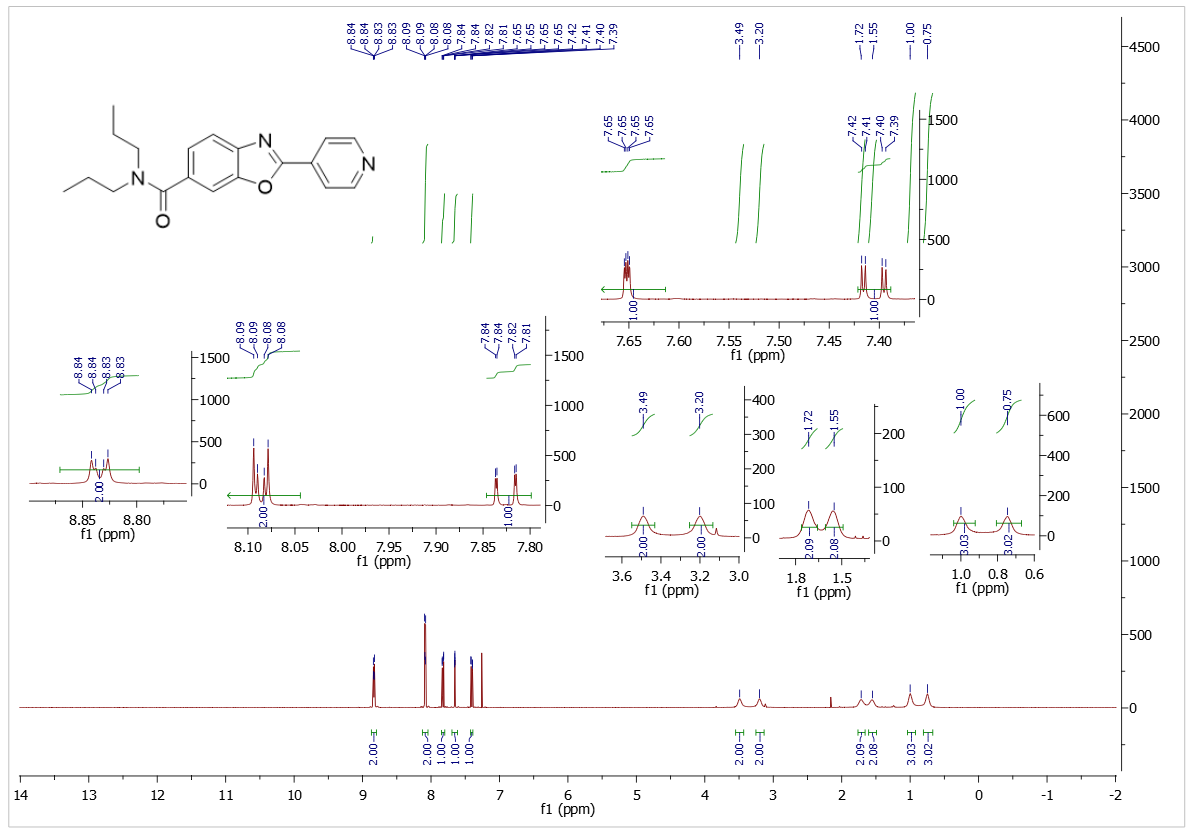


**Figure S41.** ^1^H-NMR Spectrum of compound **27**


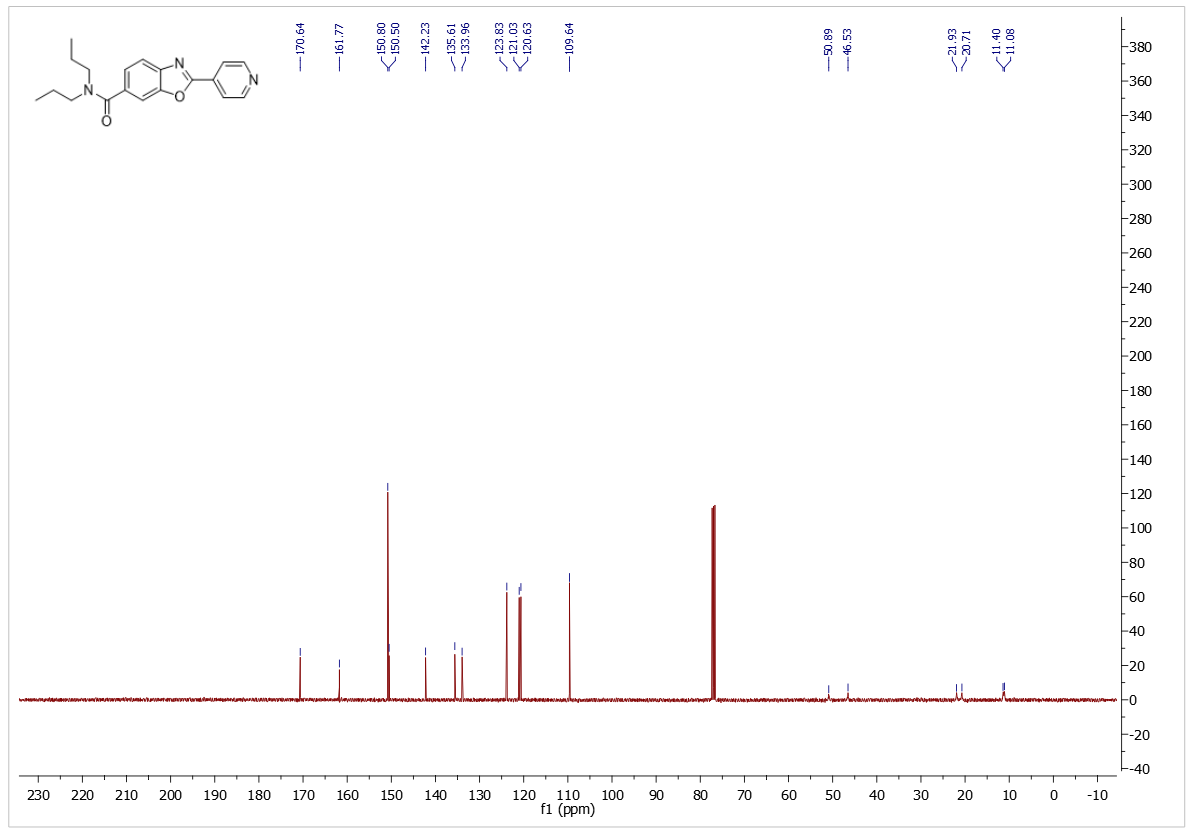


**Figure S42.** ^13^C-NMR Spectrum of compound **27**


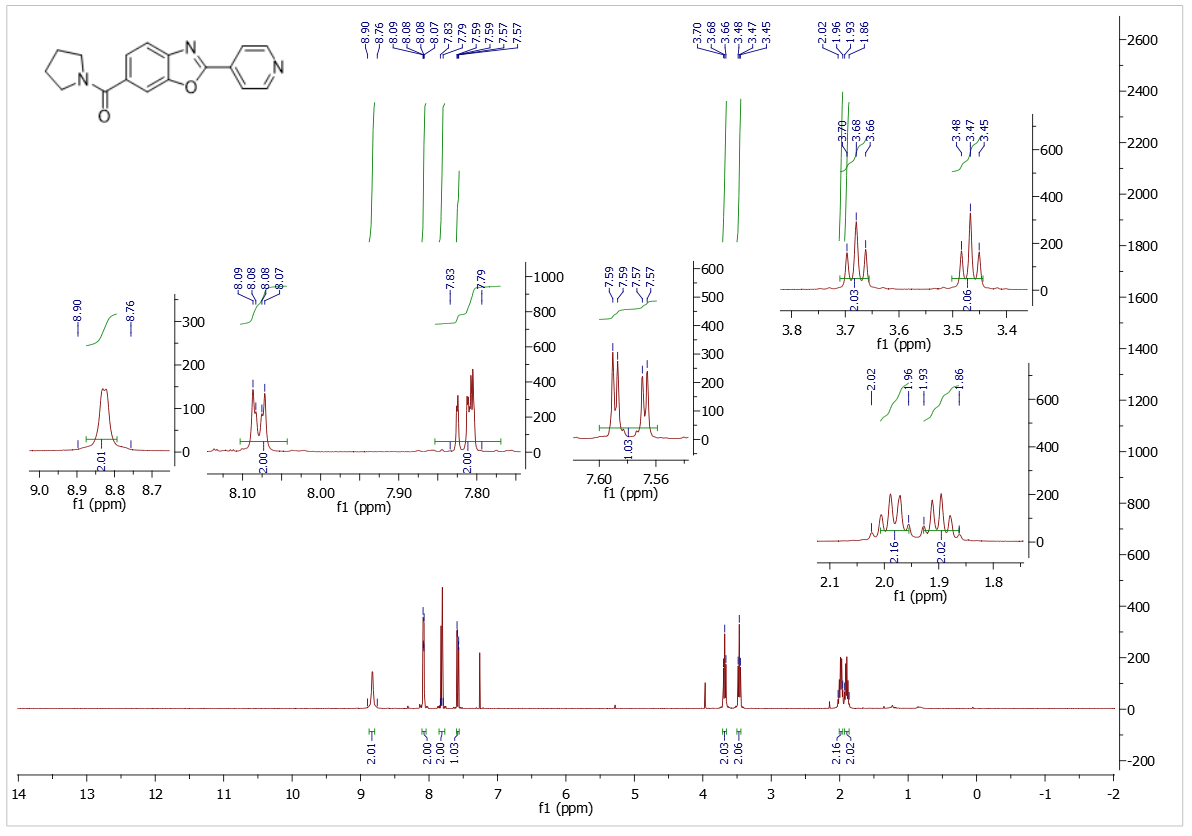


**Figure S43.** ^1^H-NMR Spectrum of compound **28**


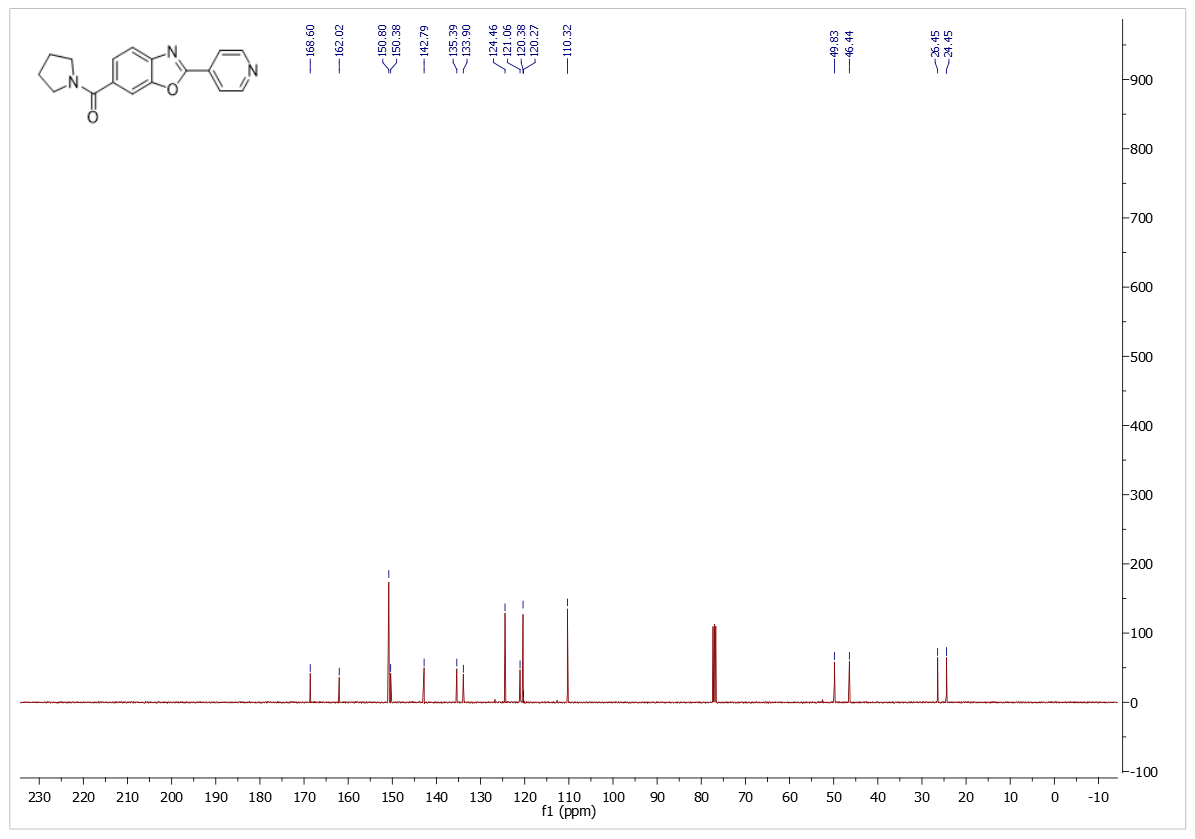


**Figure S44.** ^13^C-NMR Spectrum of compound **28**


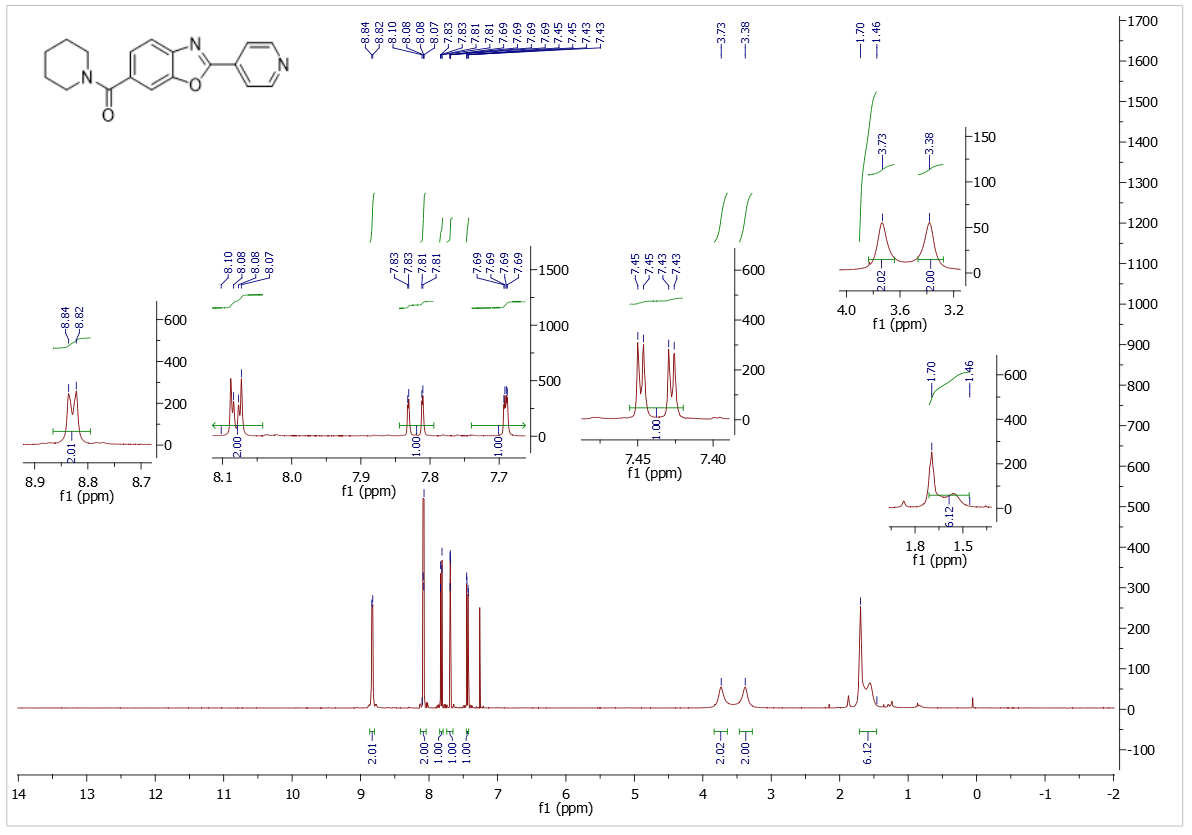


**Figure S45.** ^1^H-NMR Spectrum of compound **29**


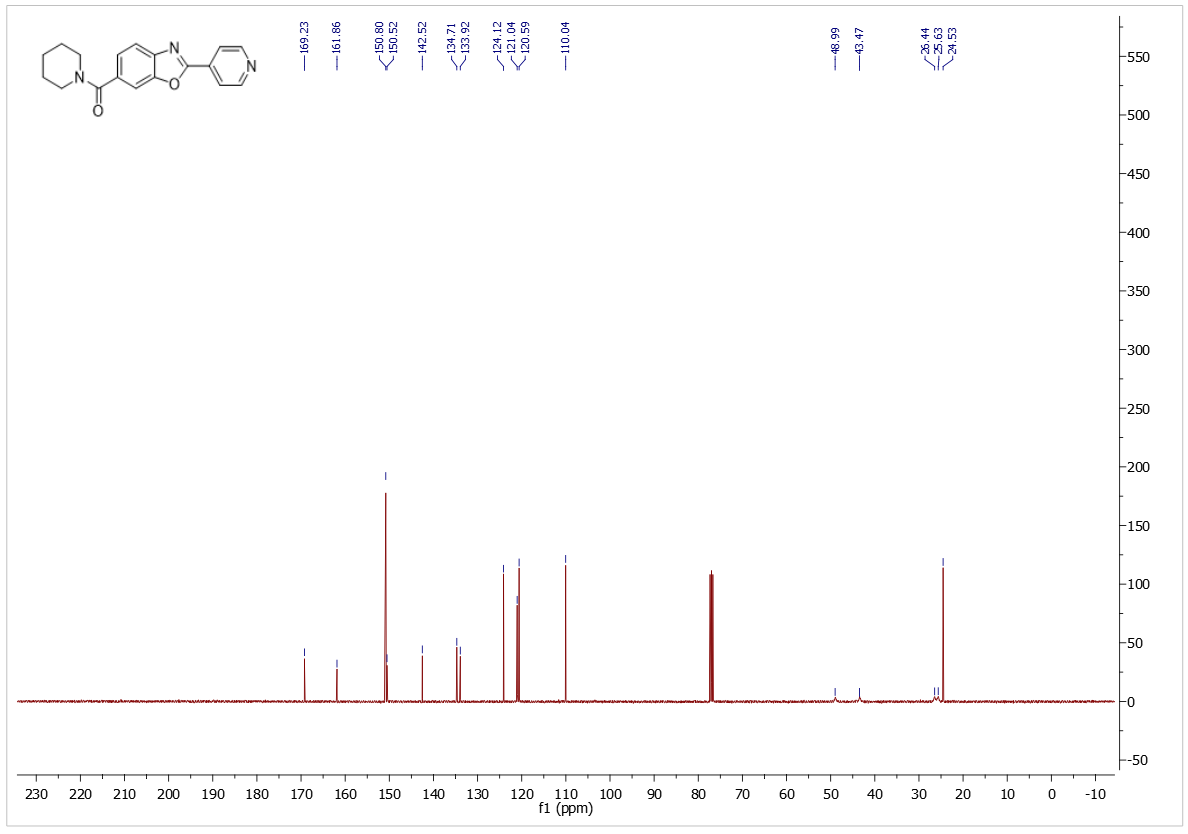


**Figure S46.** ^13^C-NMR Spectrum of compound **29**


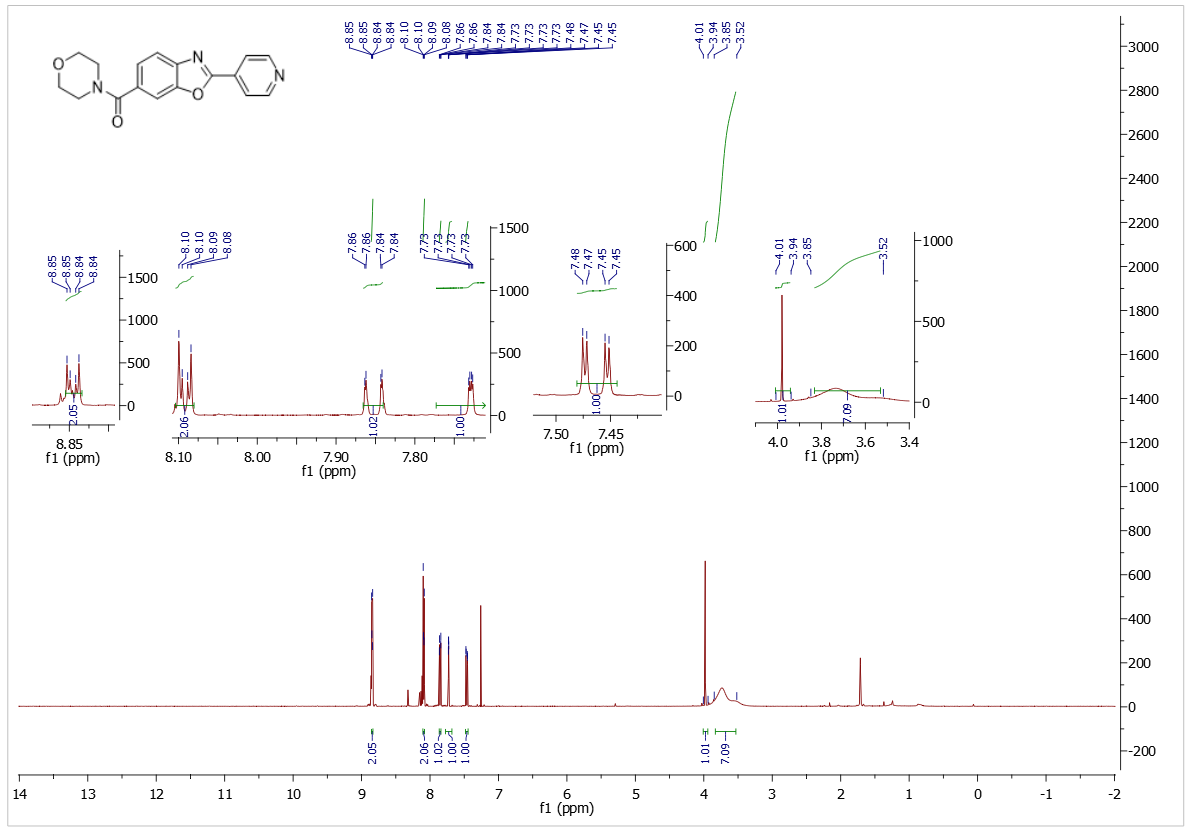


**Figure S47.** ^1^H-NMR Spectrum of compound **30**


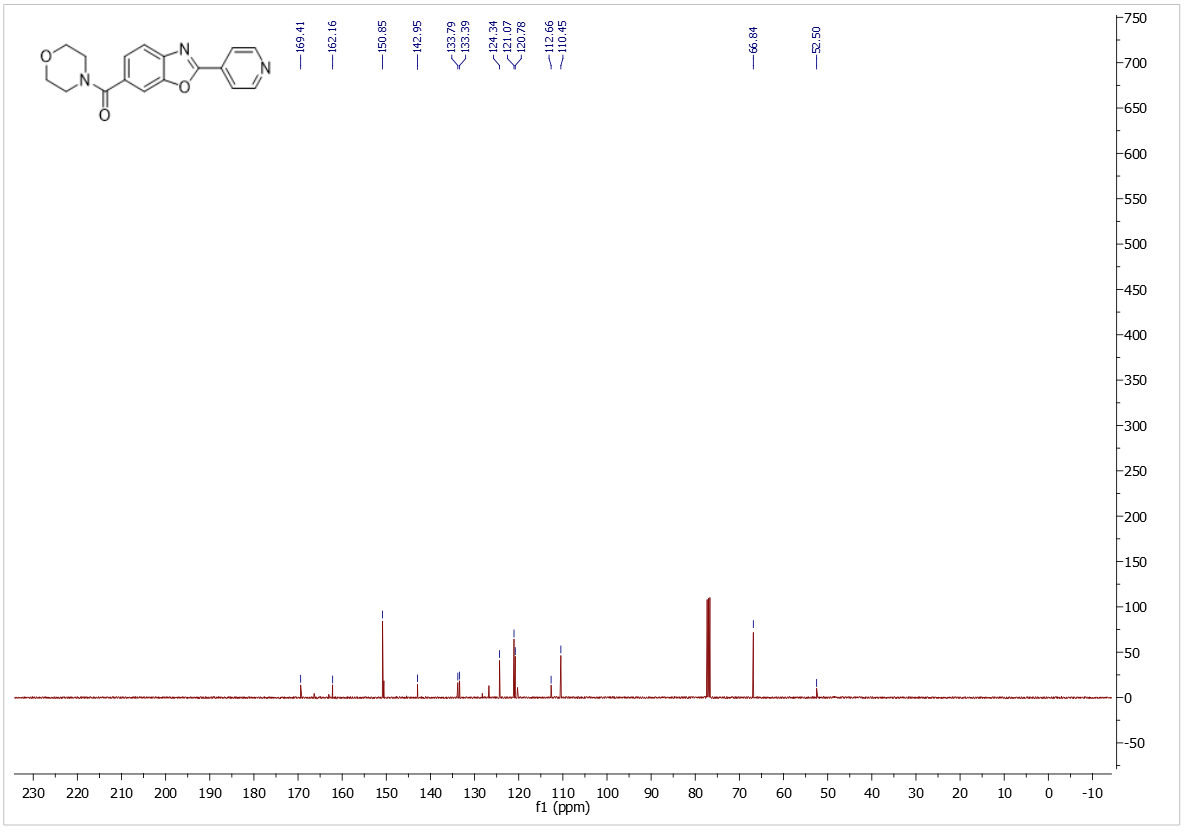


**Figure S48.** ^13^C-NMR Spectrum of compound **30**


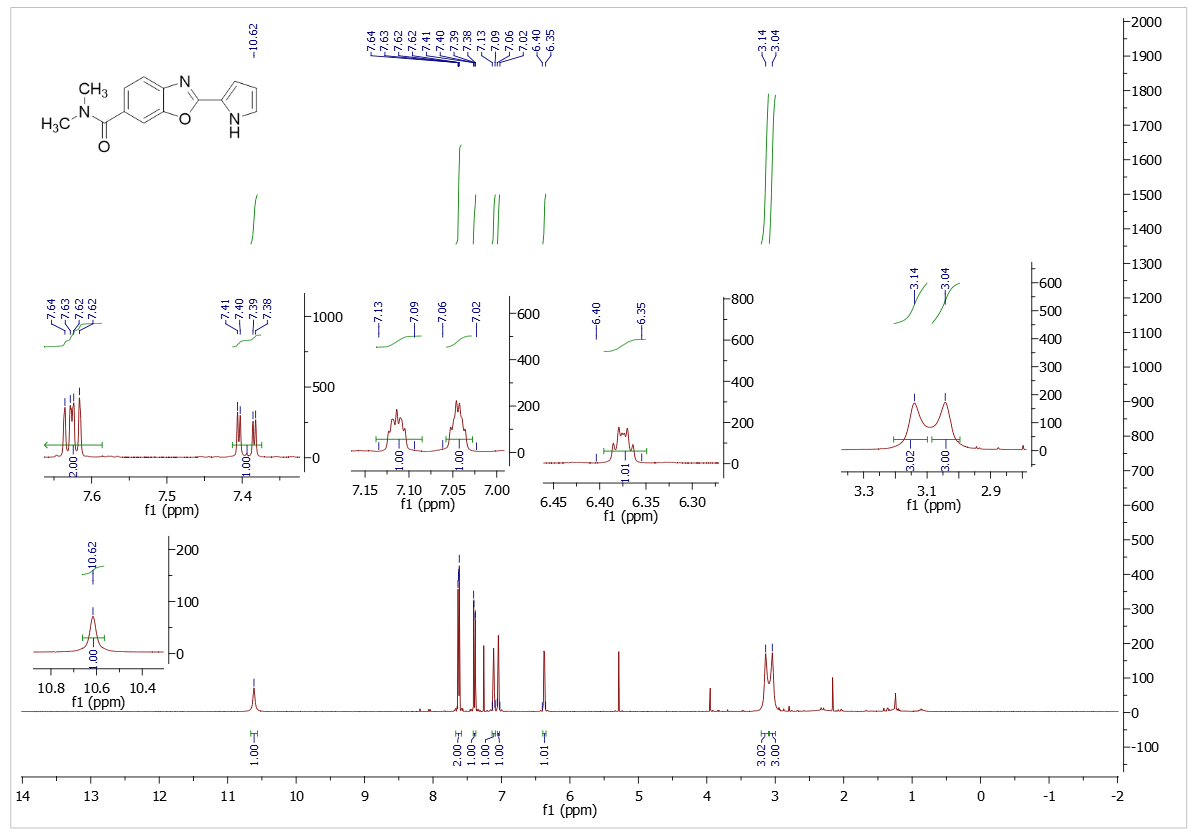


**Figure S49.** ^1^H-NMR Spectrum of compound **31**


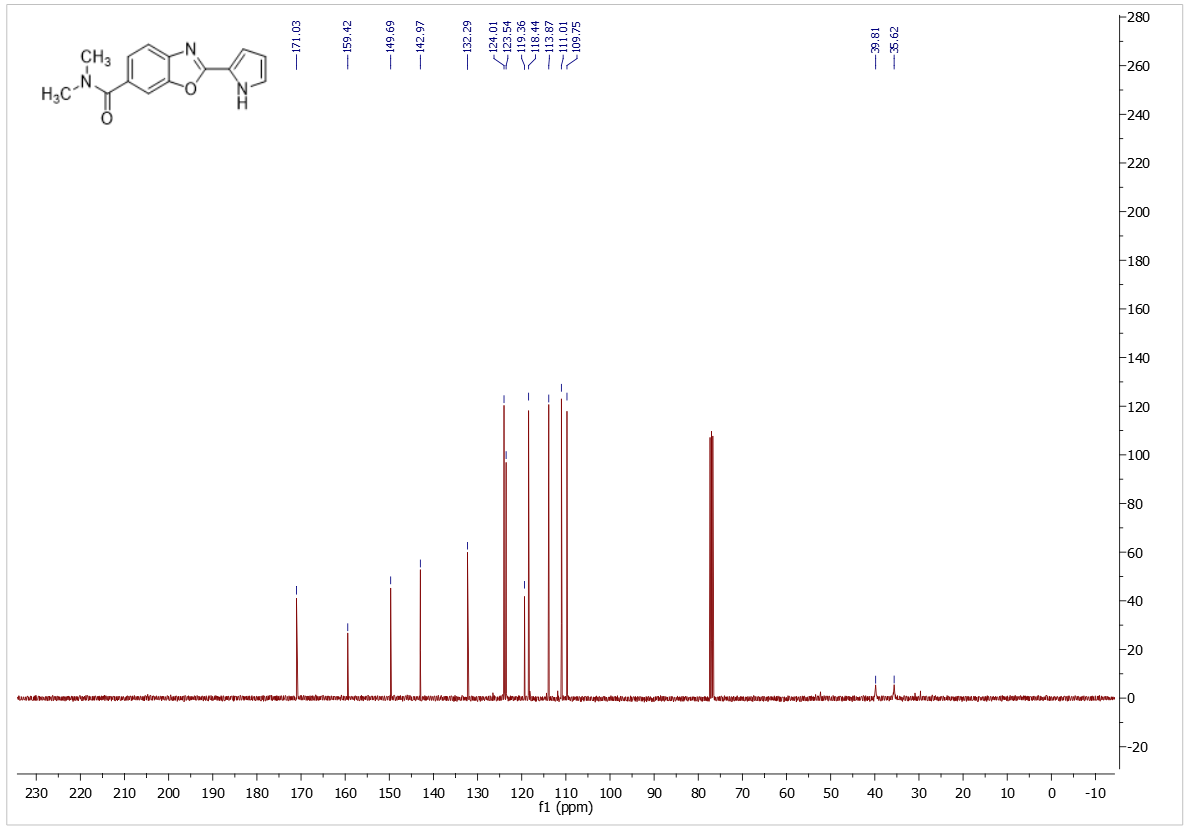


**Figure S50.** ^13^C-NMR Spectrum of compound **31**


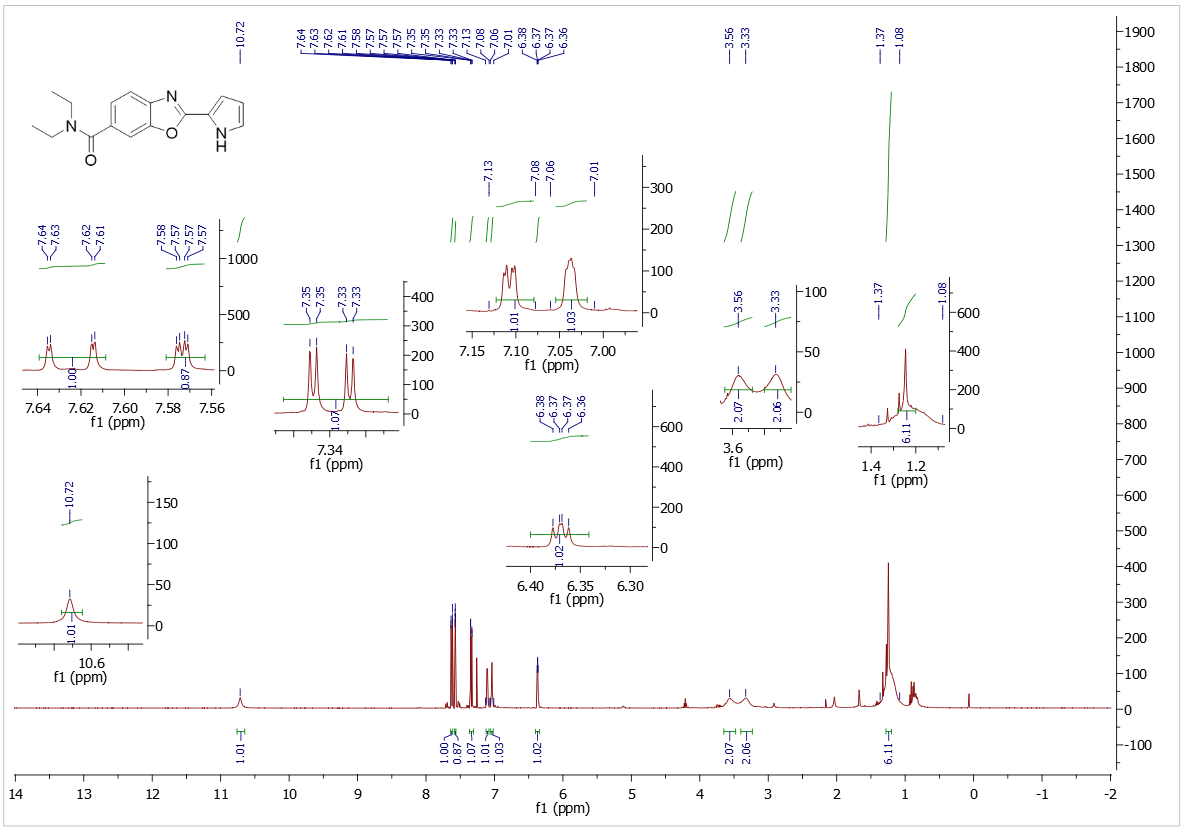


**Figure S51.** ^1^H-NMR Spectrum of compound **32**


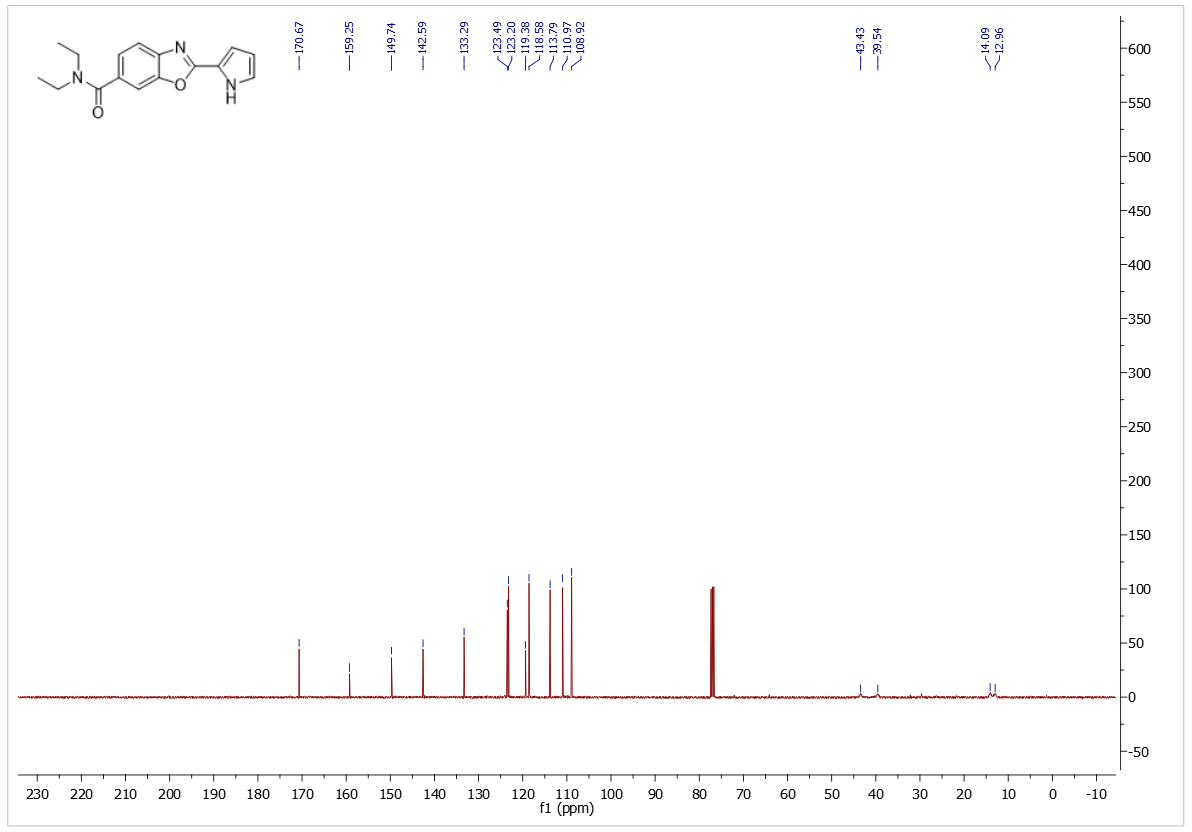


**Figure S52.** ^13^C-NMR Spectrum of compound **32**


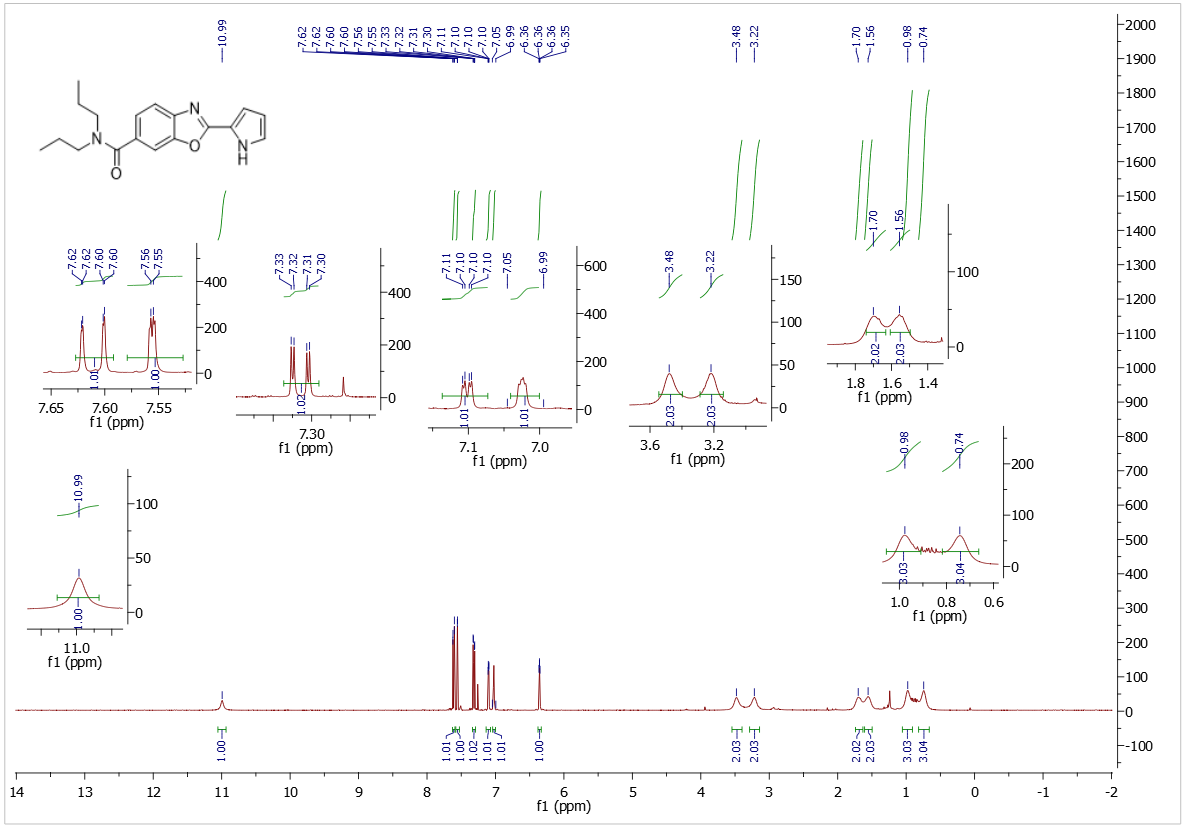


**Figure S53.** ^1^H-NMR Spectrum of compound **33**

**
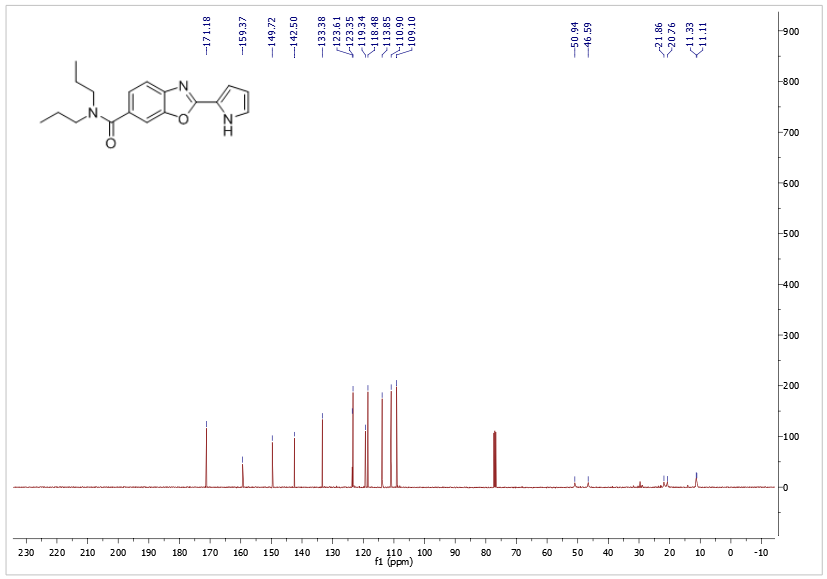
**

**Figure S54.** ^13^C-NMR Spectrum of compound **33**


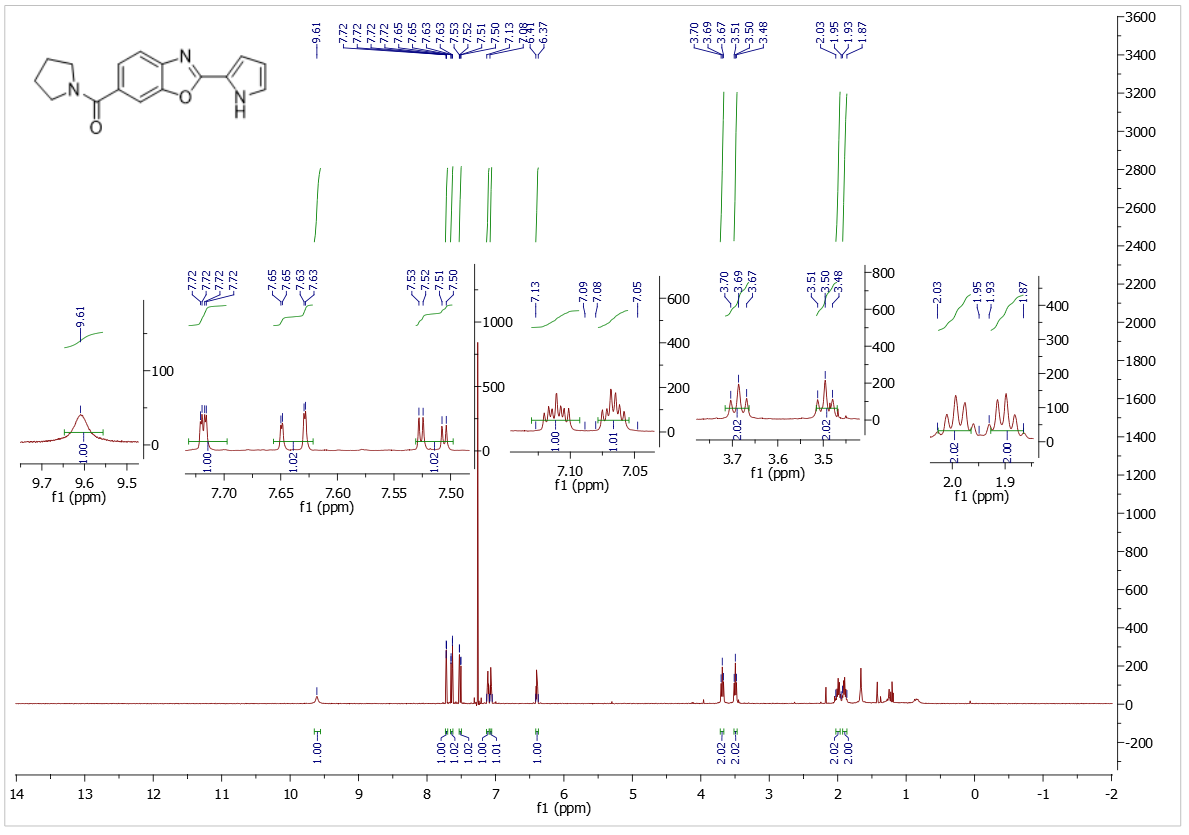


**Figure S55.** ^1^H-NMR Spectrum of compound **34**


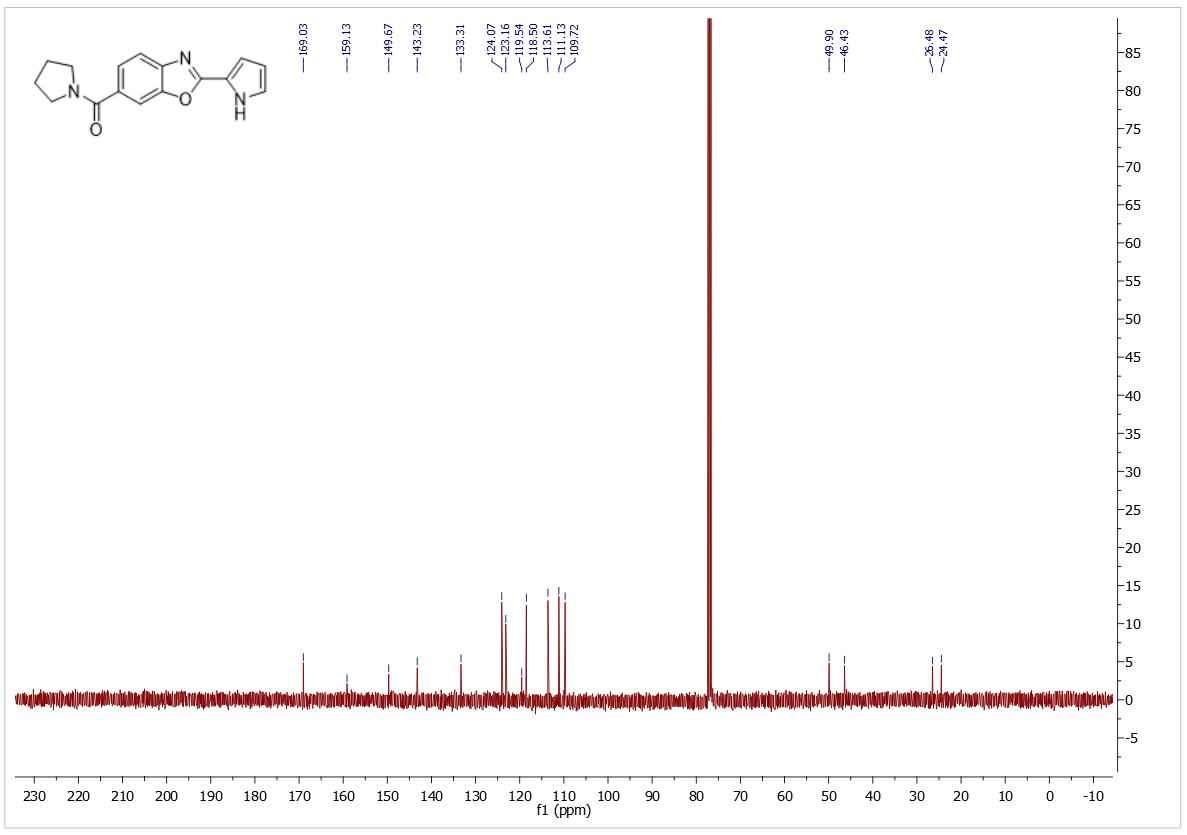


**Figure S56.** ^13^C-NMR Spectrum of compound **34**


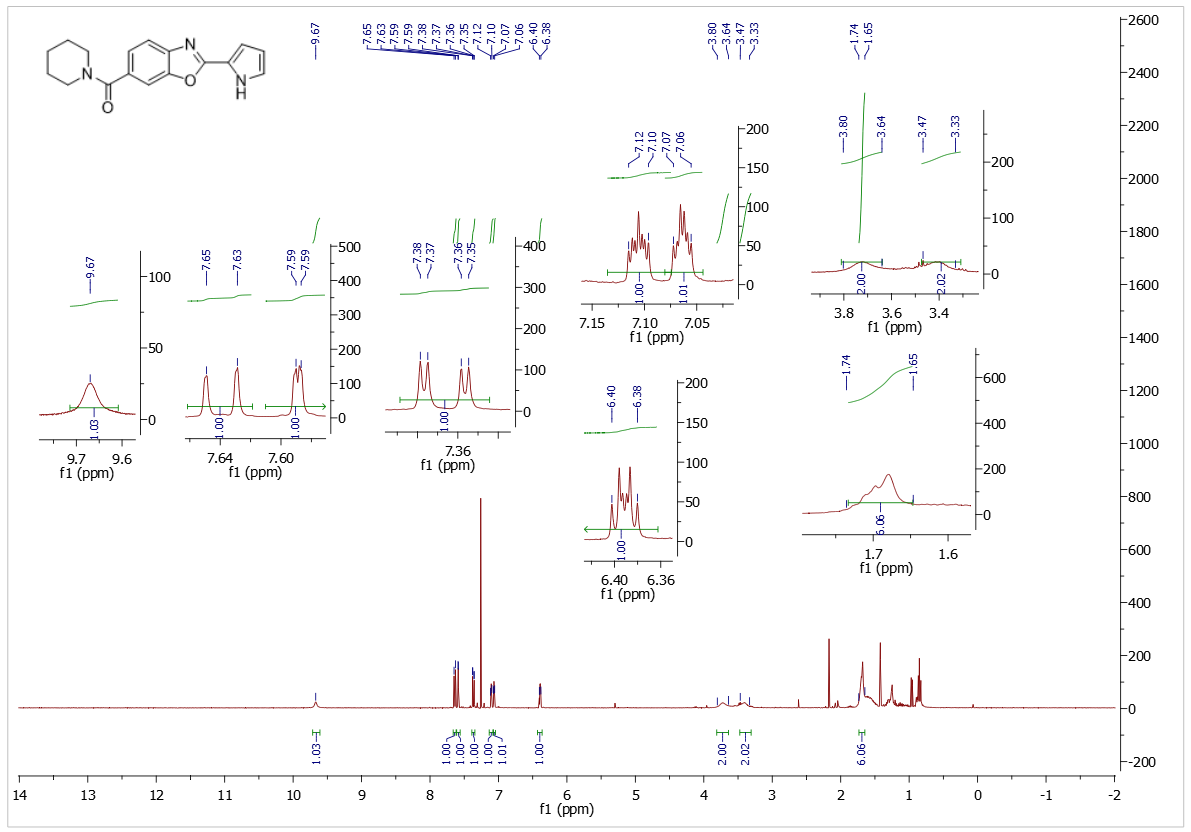


**Figure S57.** ^1^H-NMR Spectrum of compound **35**


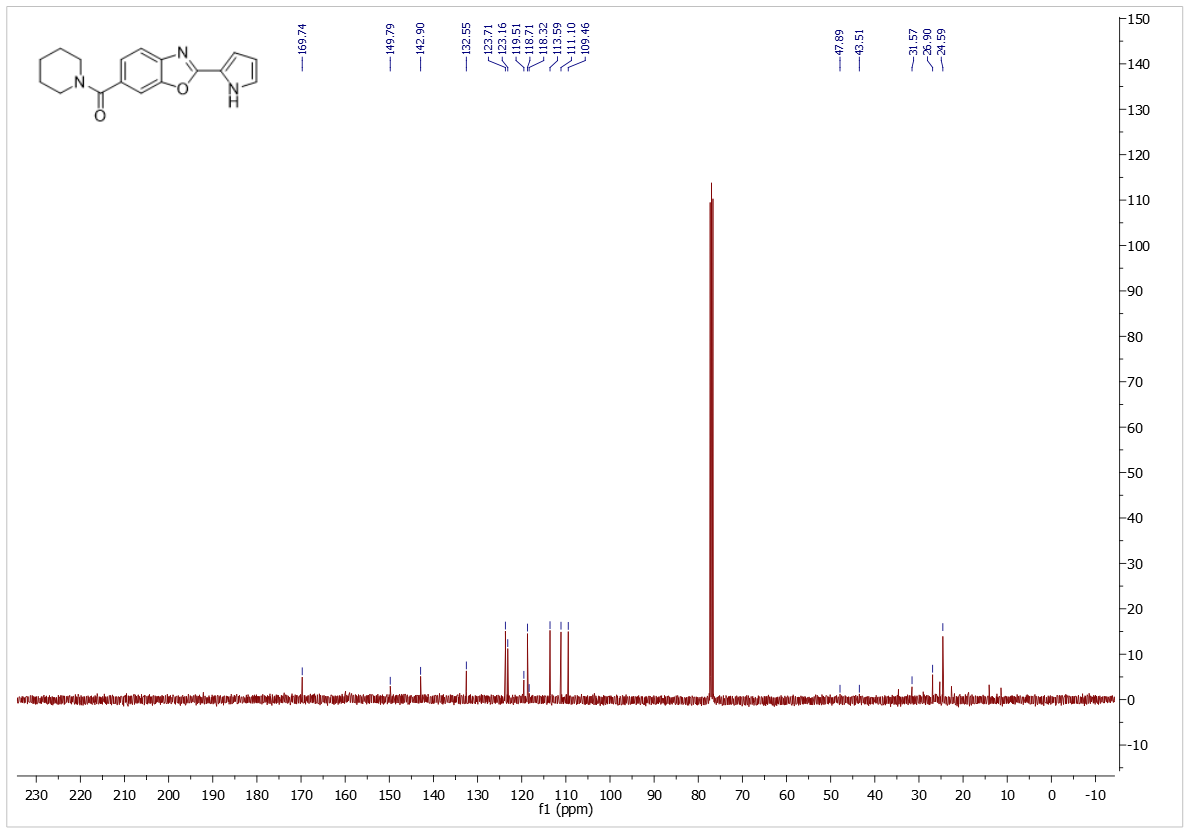


**Figure S58.** ^13^C-NMR Spectrum of compound **35**


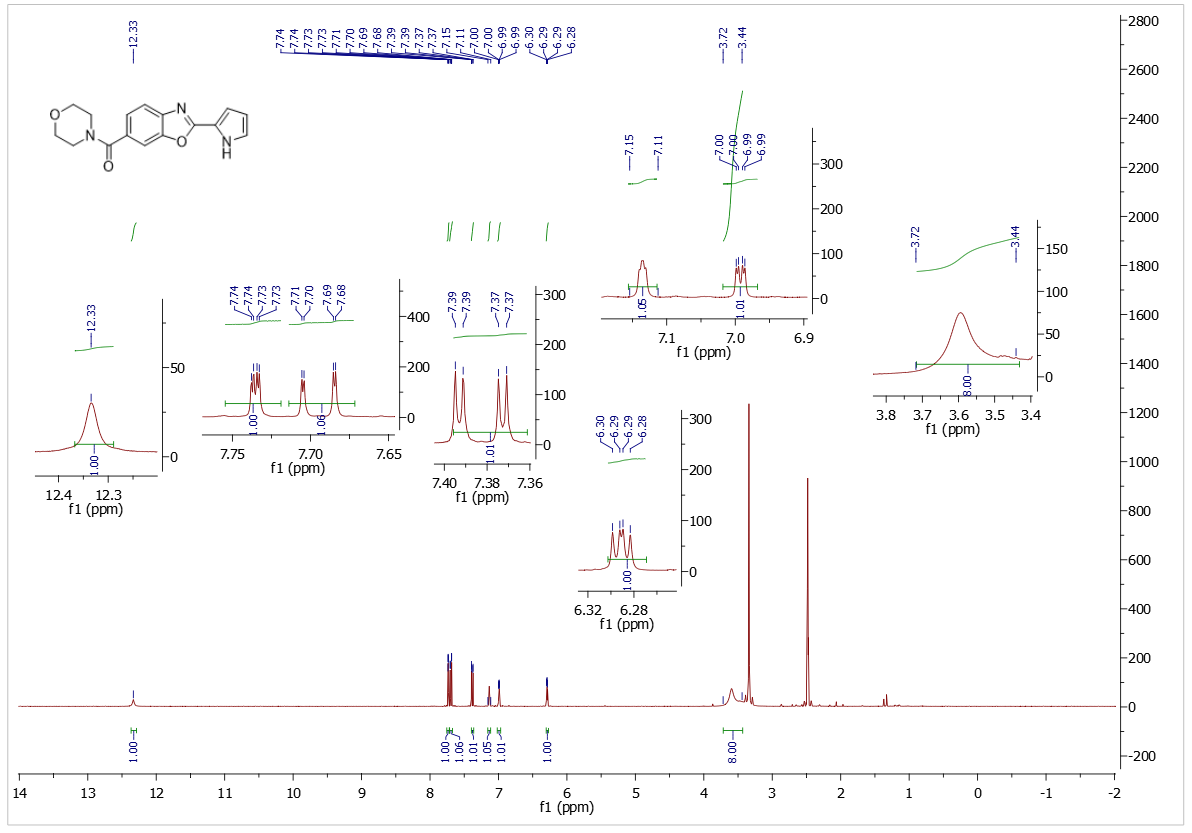


**Figure S59.** ^1^H-NMR Spectrum of compound **36**


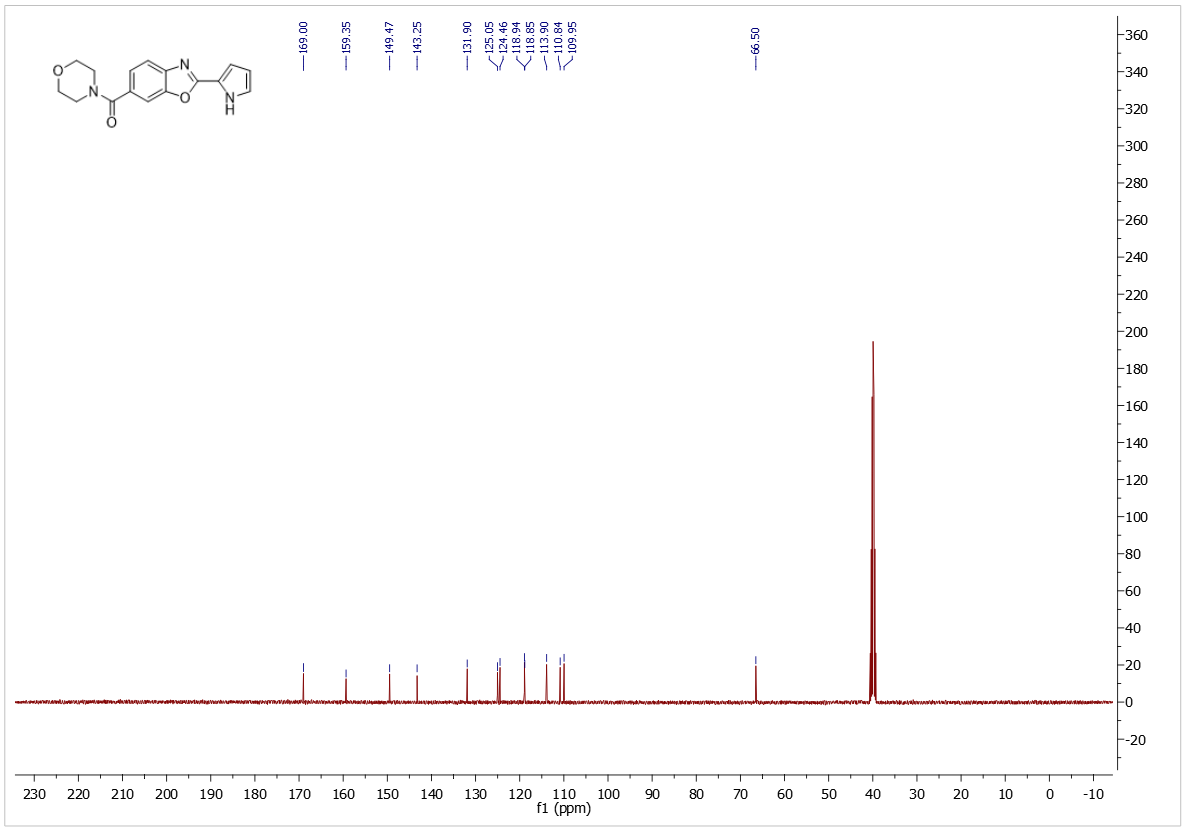


**Figure S60.** ^13^C-NMR Spectrum of compound **36**


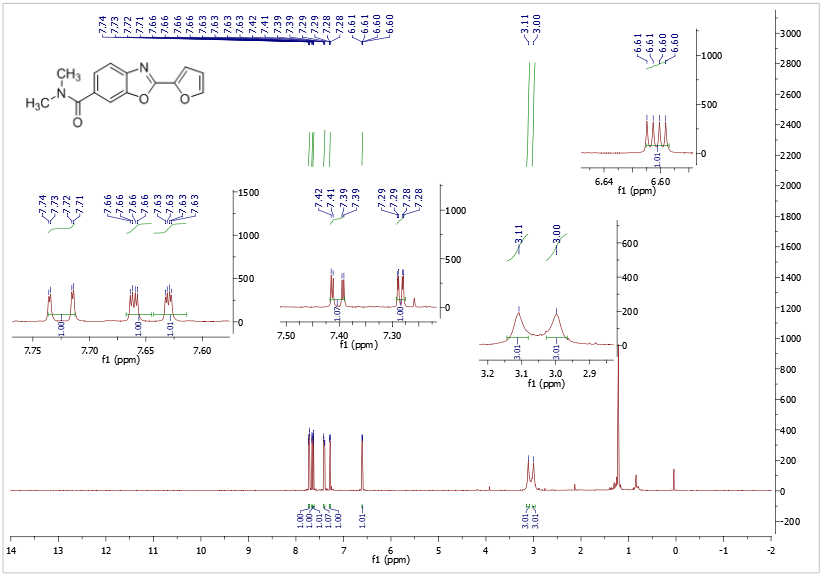


**Figure S61.** ^1^H-NMR Spectrum of compound **37**


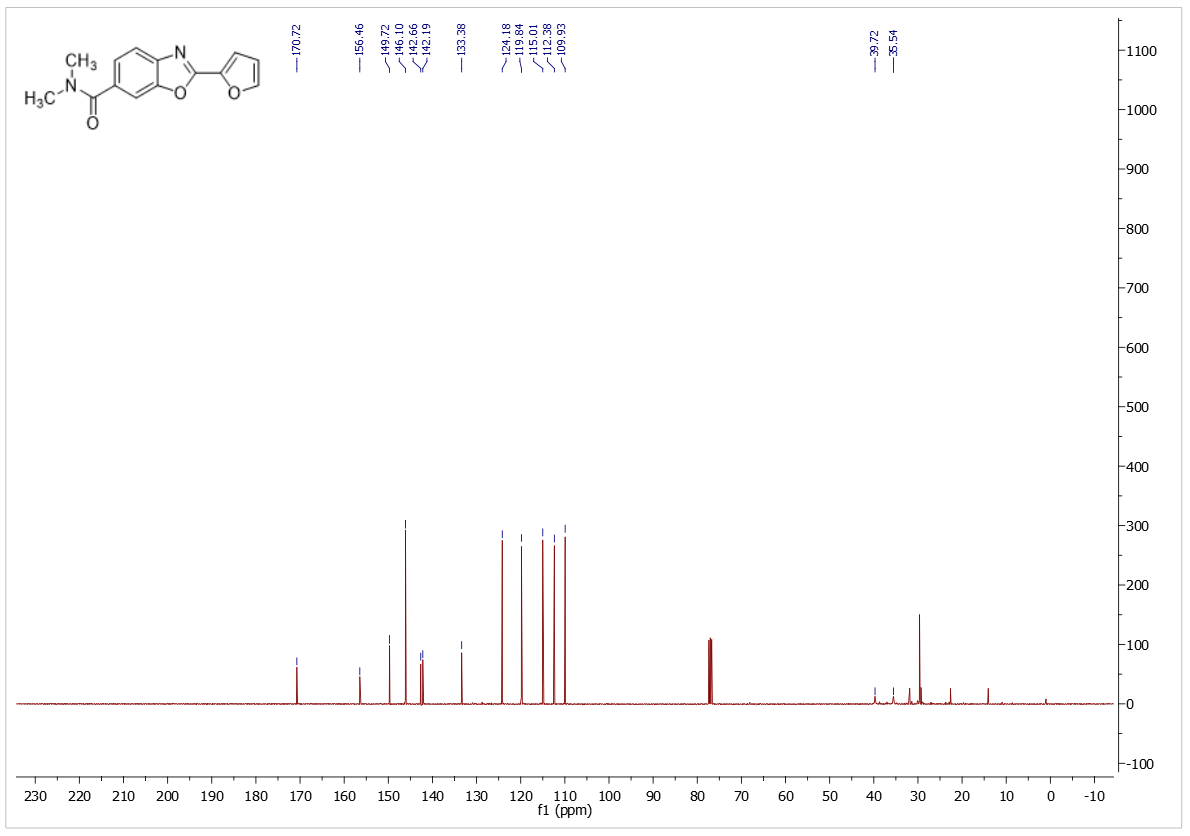


**Figure S62.** ^13^C-NMR Spectrum of compound **37**


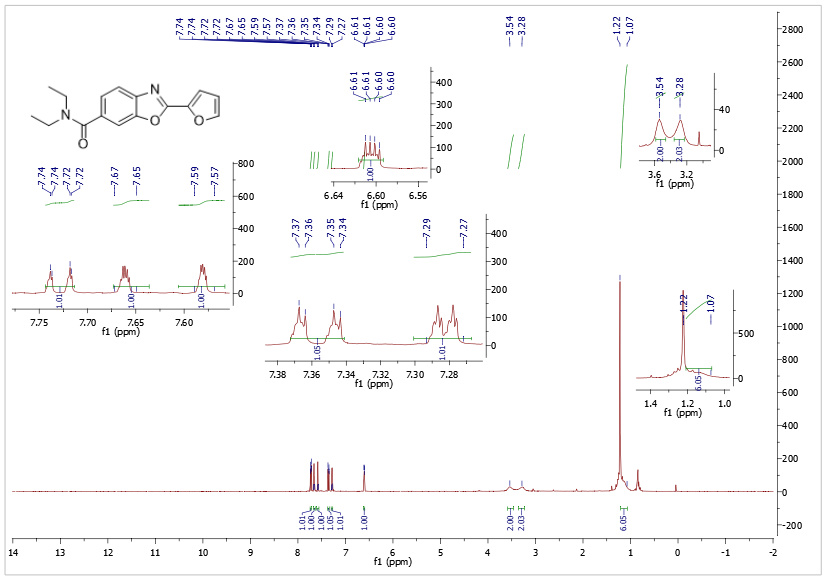


**Figure S63.** ^1^H-NMR Spectrum of compound **38**


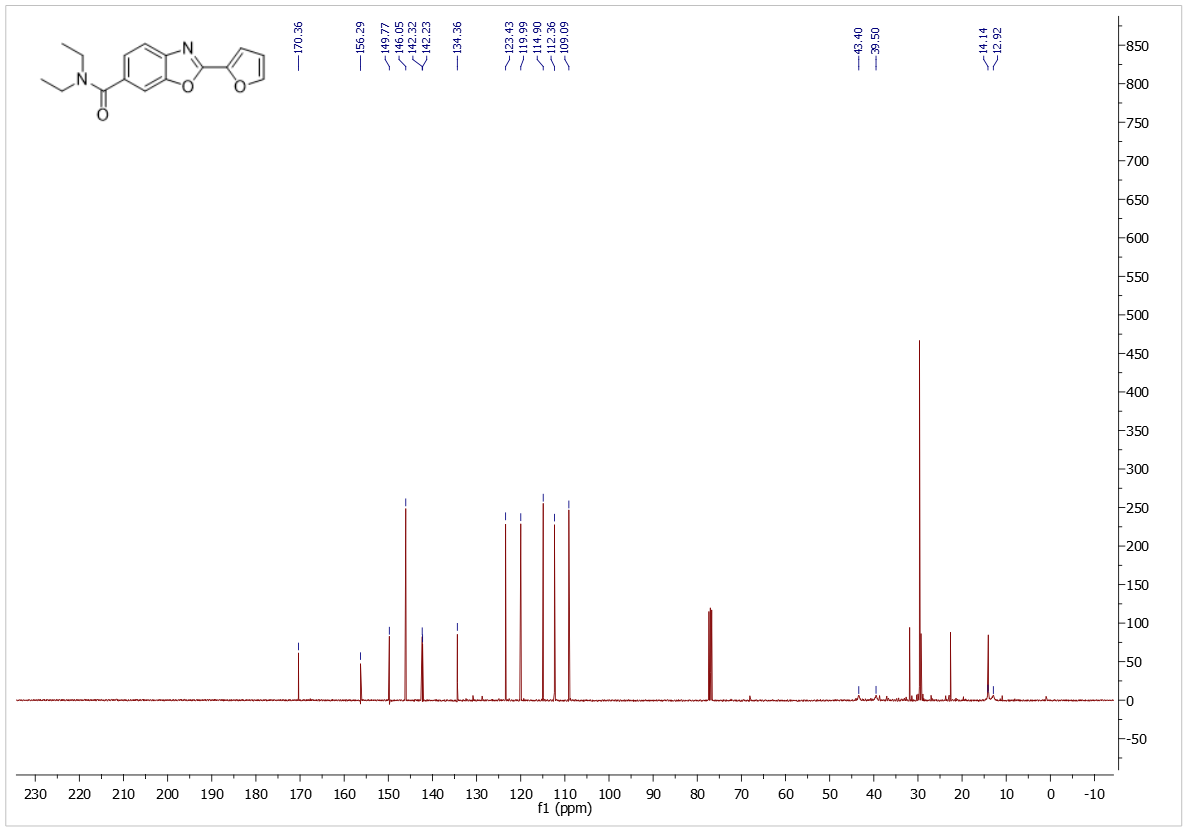


**Figure S64.** ^13^C-NMR Spectrum of compound **38**

**
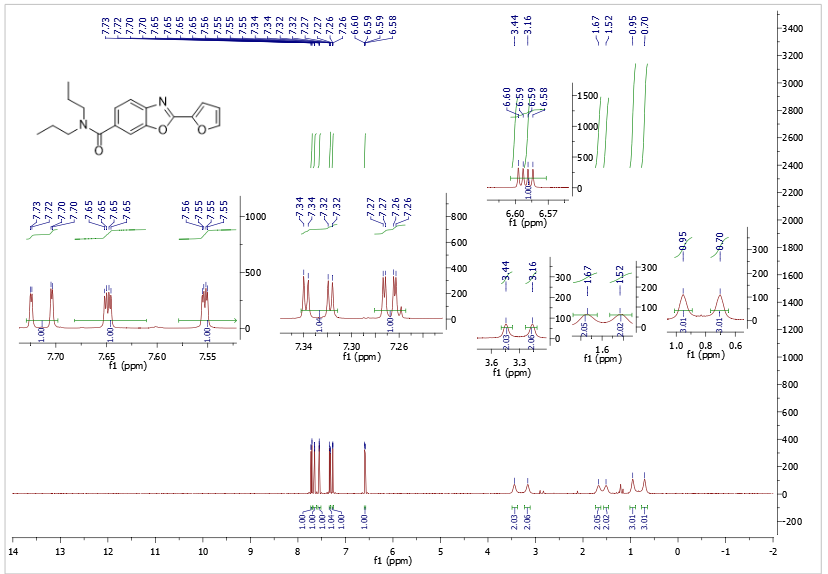
**

**Figure S65.** ^1^H-NMR Spectrum of compound **39**

**
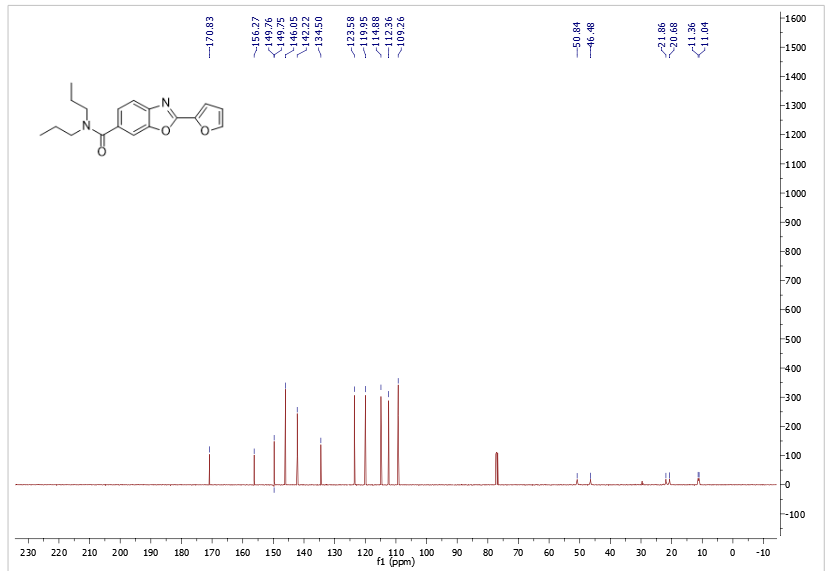
**

**Figure S66.** ^13^C-NMR Spectrum of compound **39**


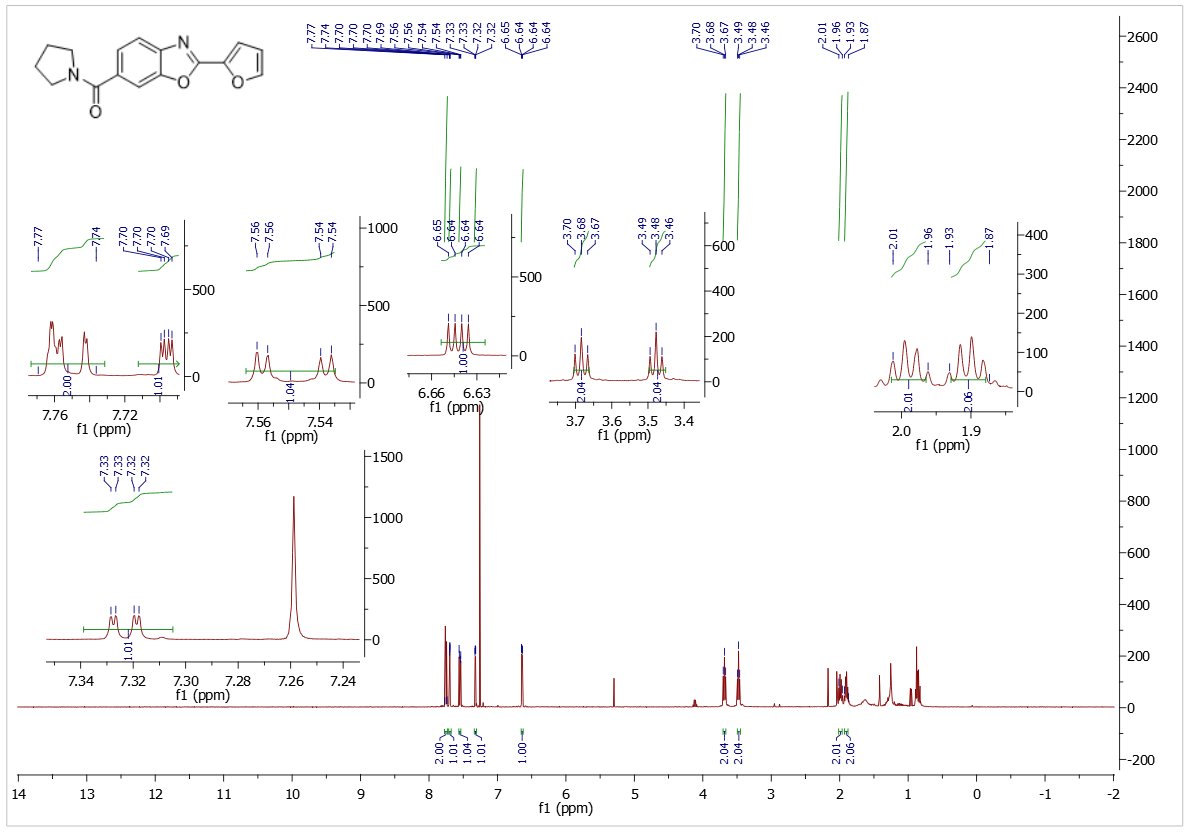


**Figure S67.** ^1^H-NMR Spectrum of compound **40**


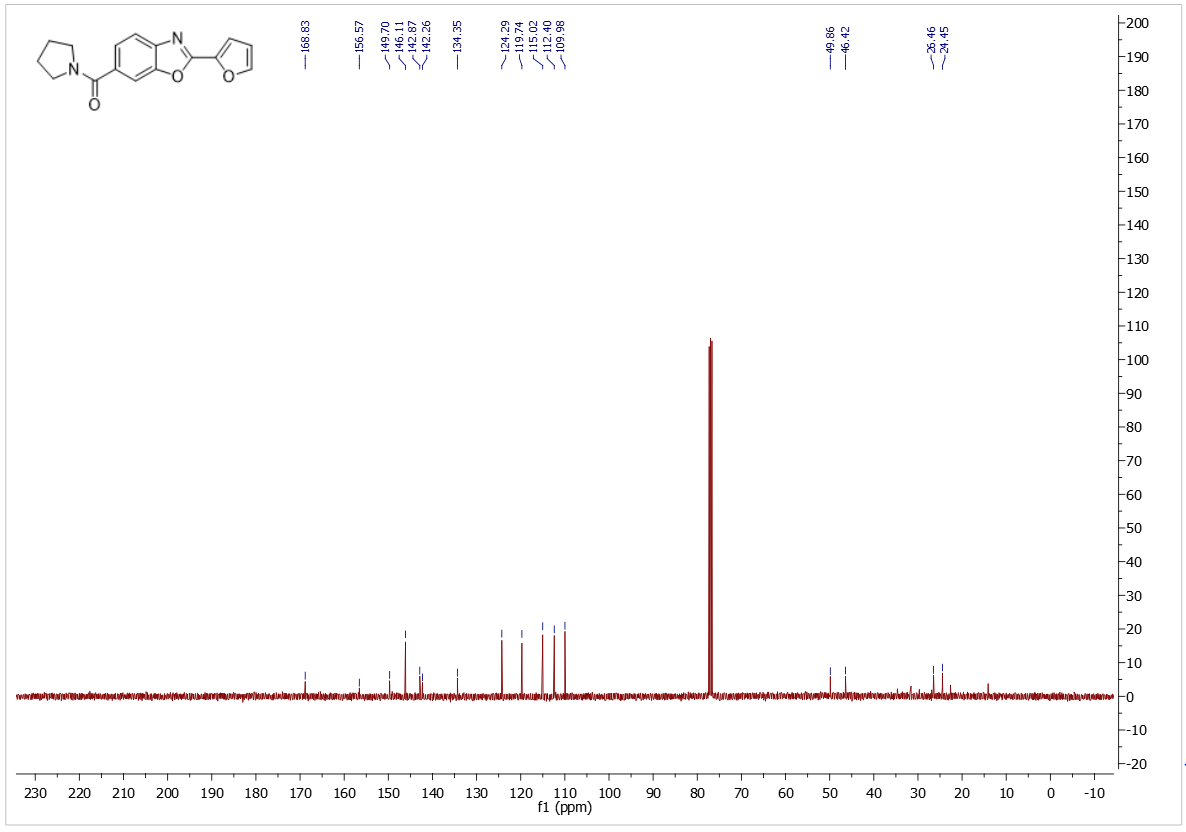


**Figure S68.** ^13^C-NMR Spectrum of compound **40**


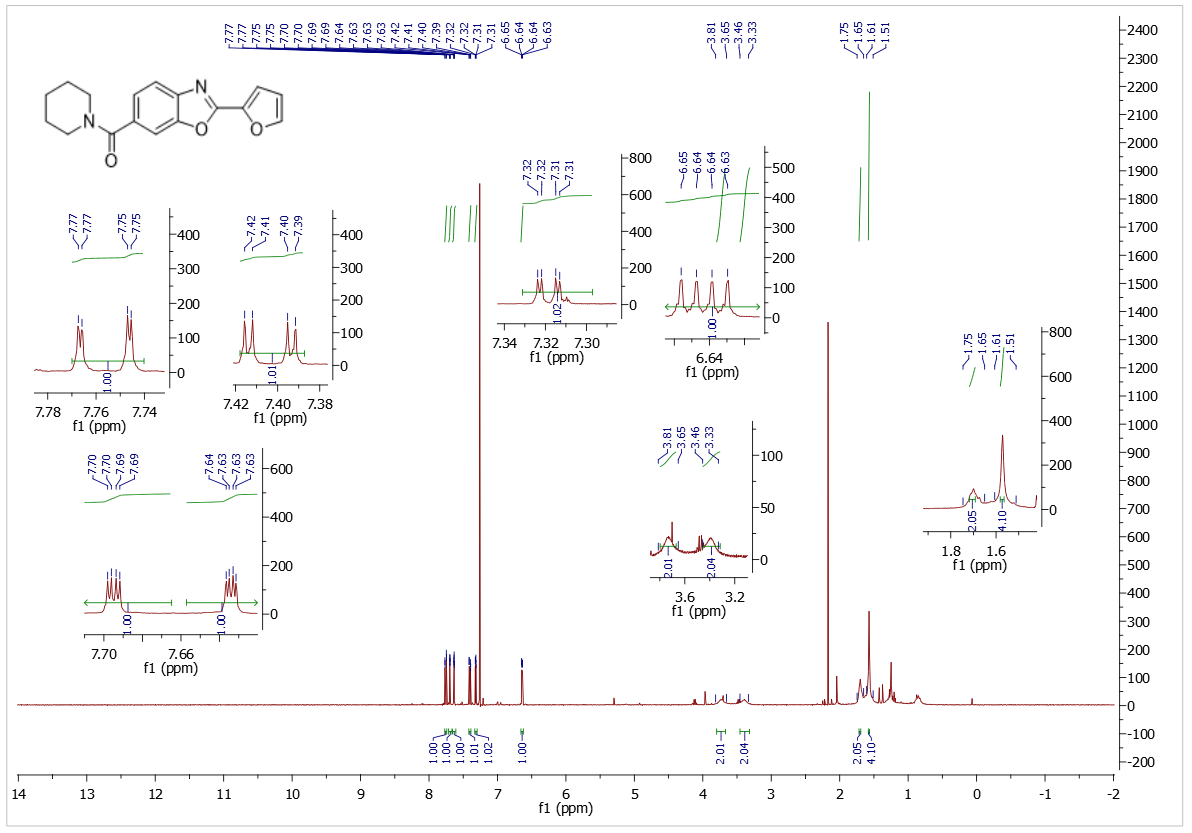


**Figure S69.** ^1^H-NMR Spectrum of compound **41**


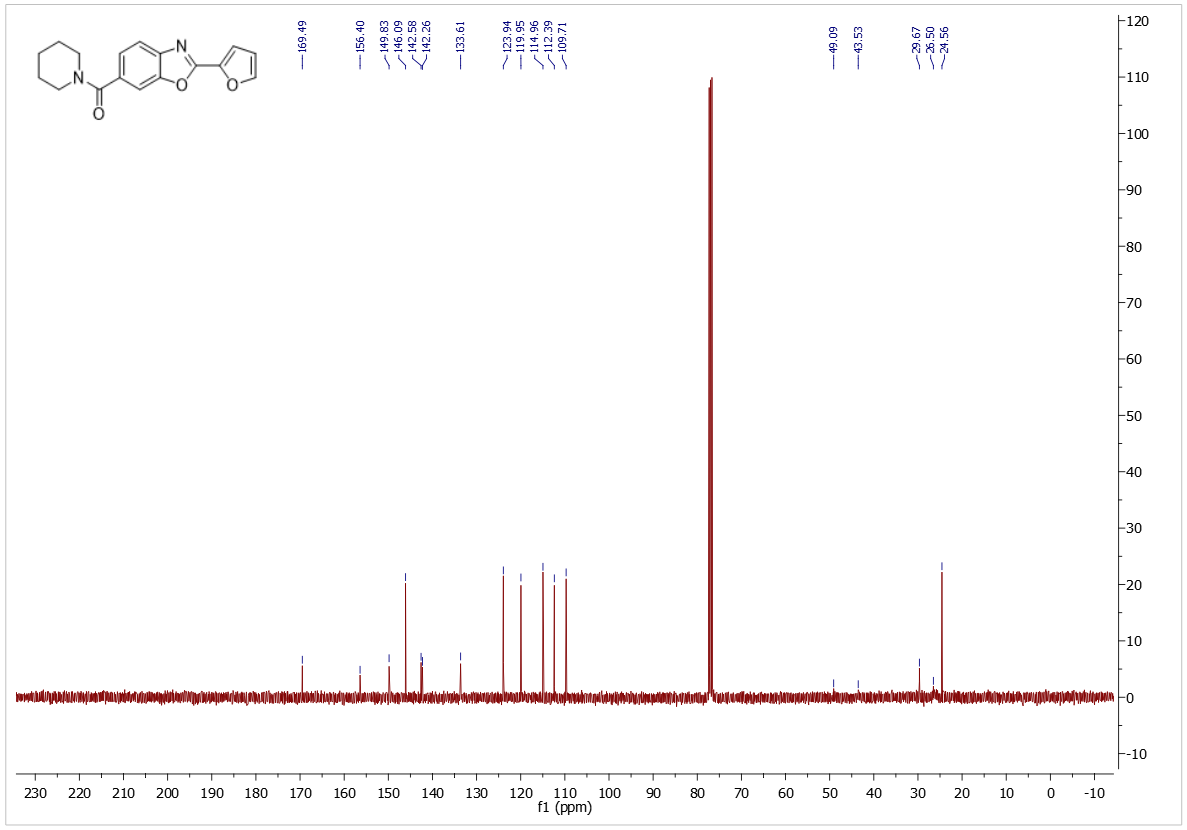


**Figure S70.** ^13^C-NMR Spectrum of compound **41**


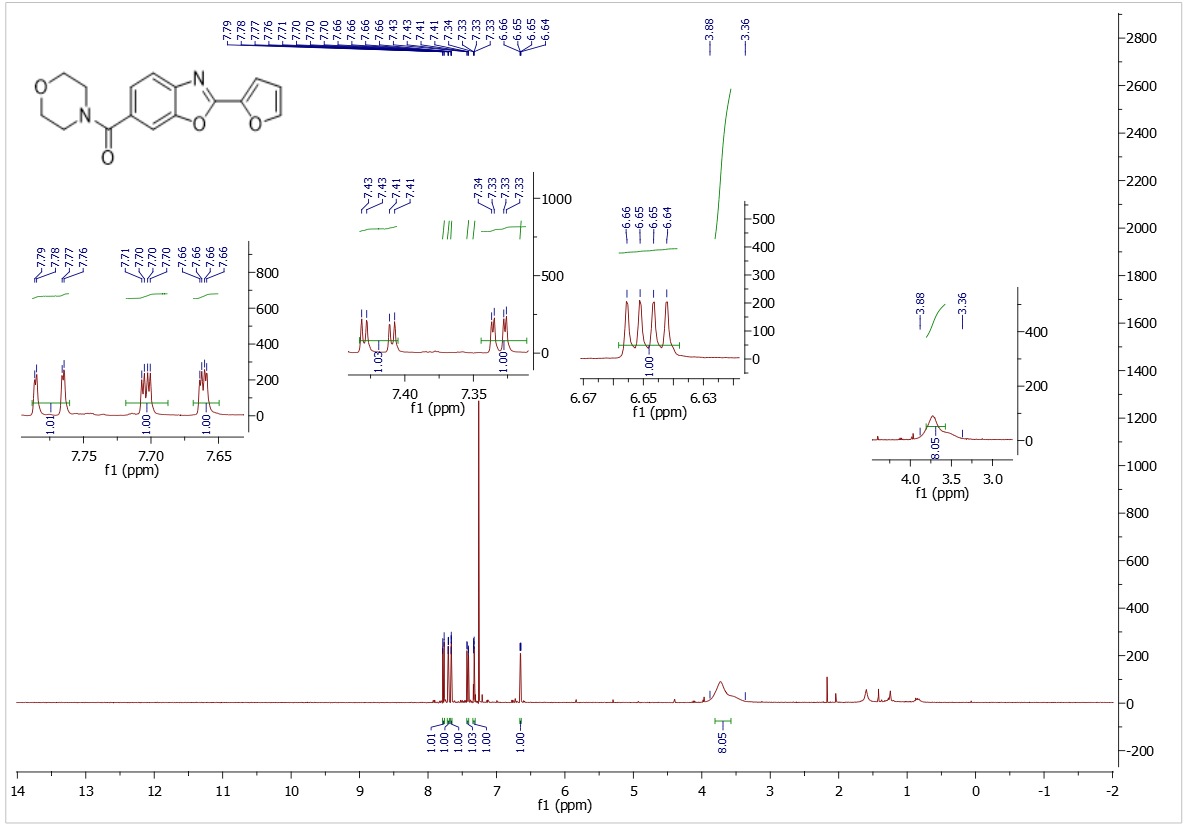


**Figure S71.** ^1^H-NMR Spectrum of compound **42**


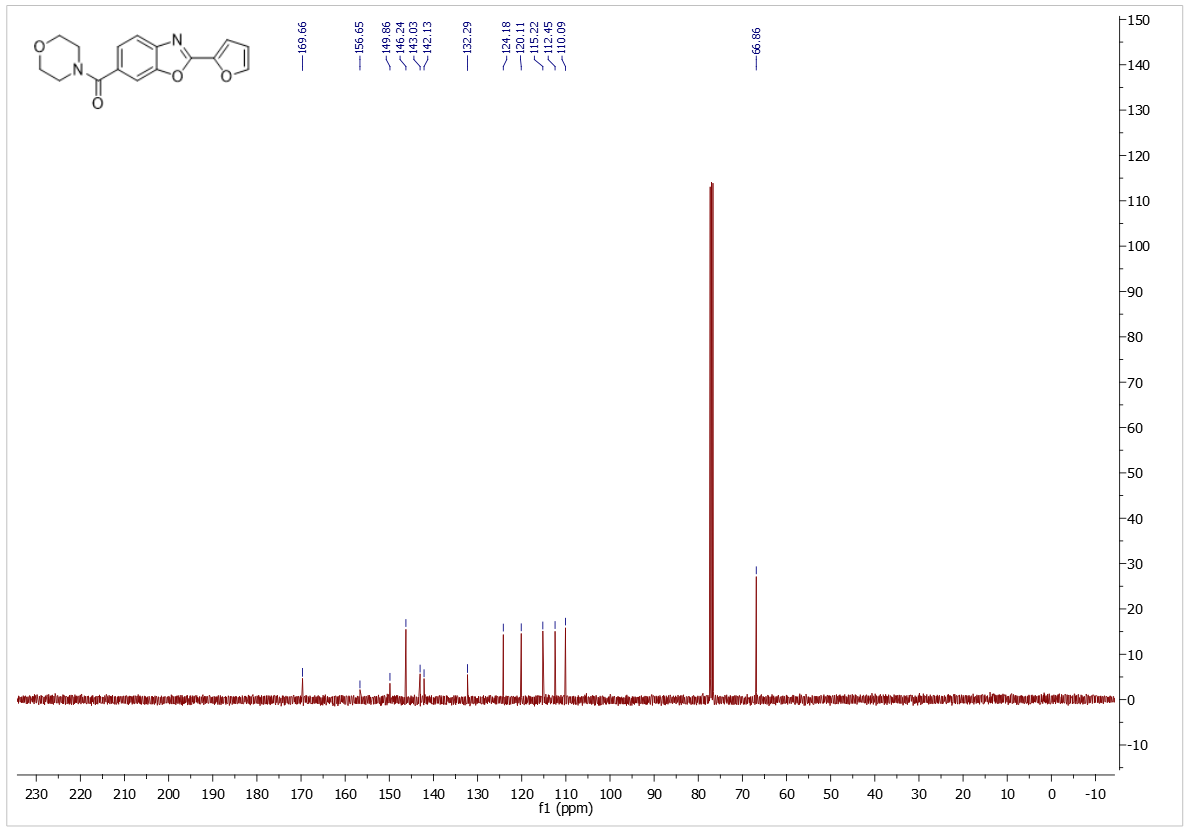


**Figure S72.** ^13^C-NMR Spectrum of compound **42**


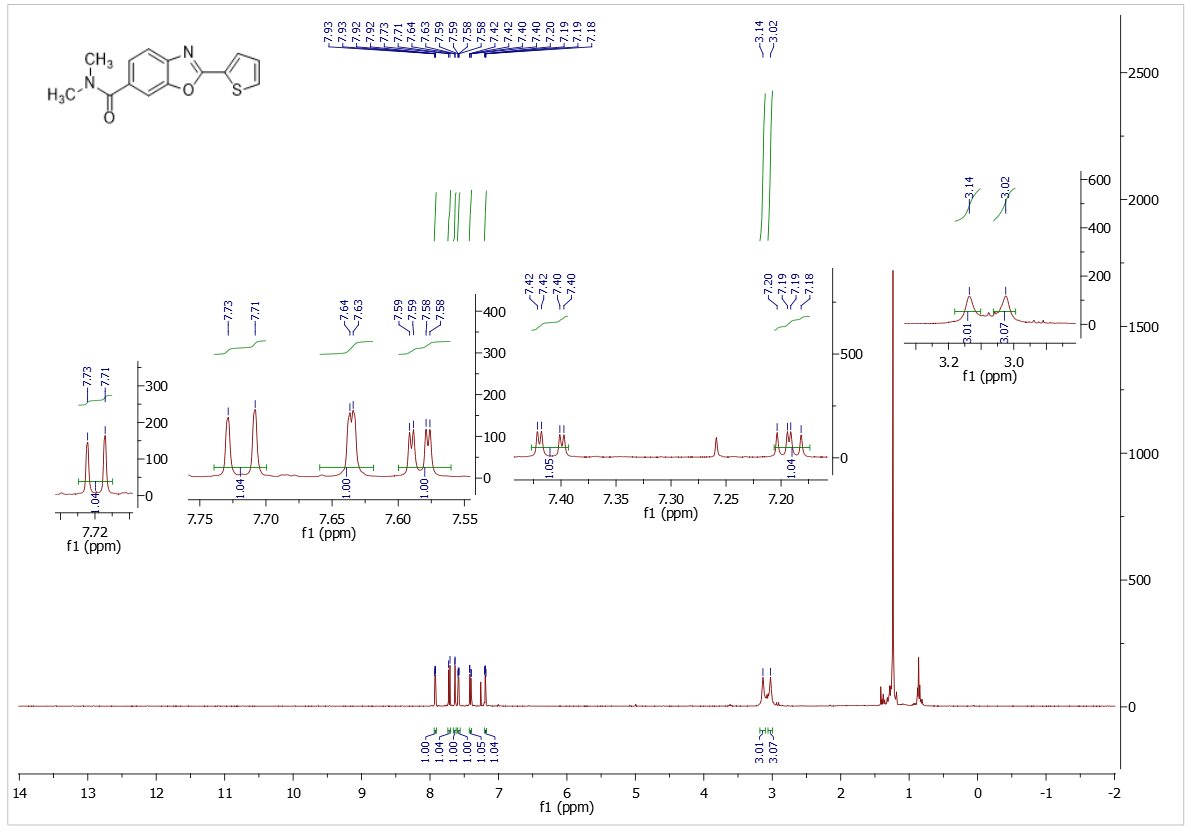


**Figure S73.** ^1^H-NMR Spectrum of compound **43**


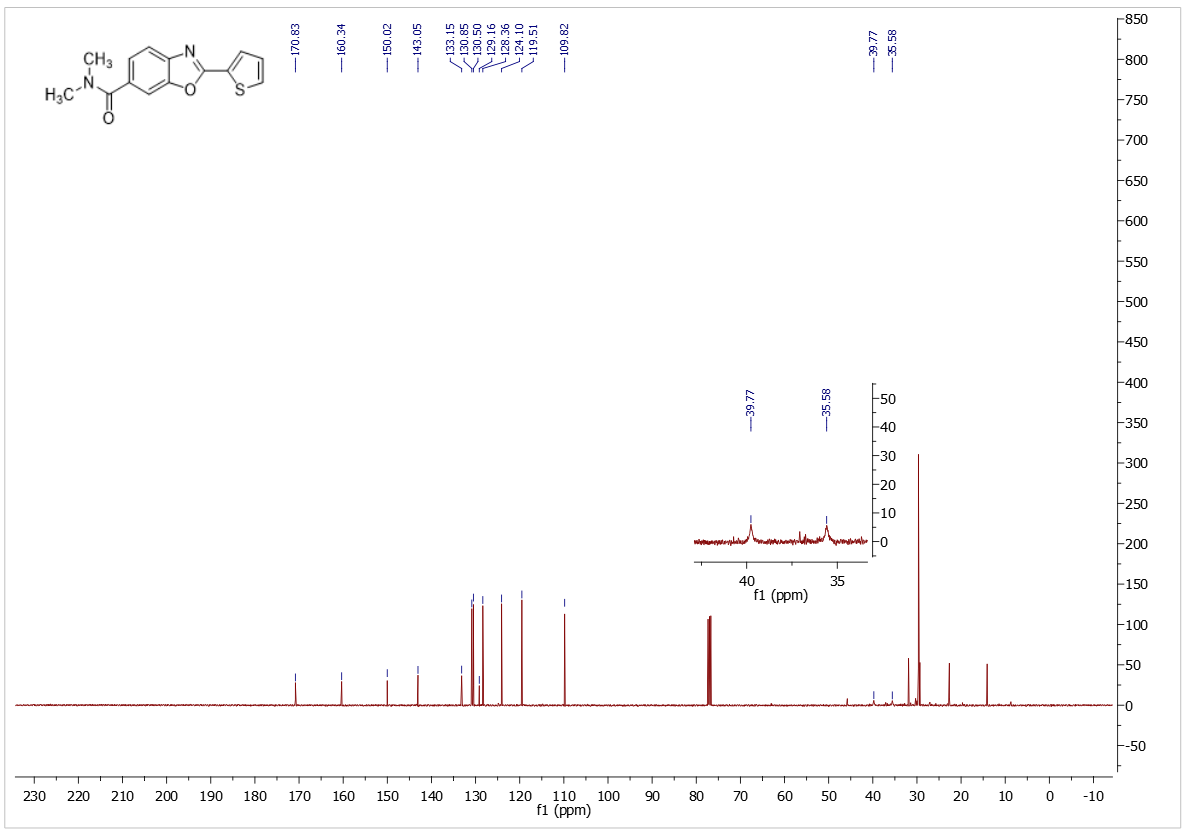


**Figure S74.** ^13^C-NMR Spectrum of compound **43**


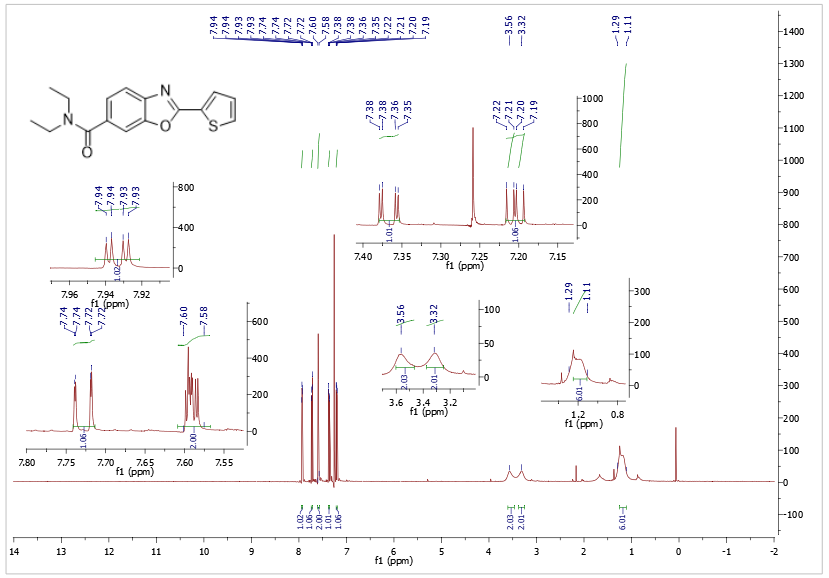


**Figure S75.** ^1^H-NMR Spectrum of compound **44**


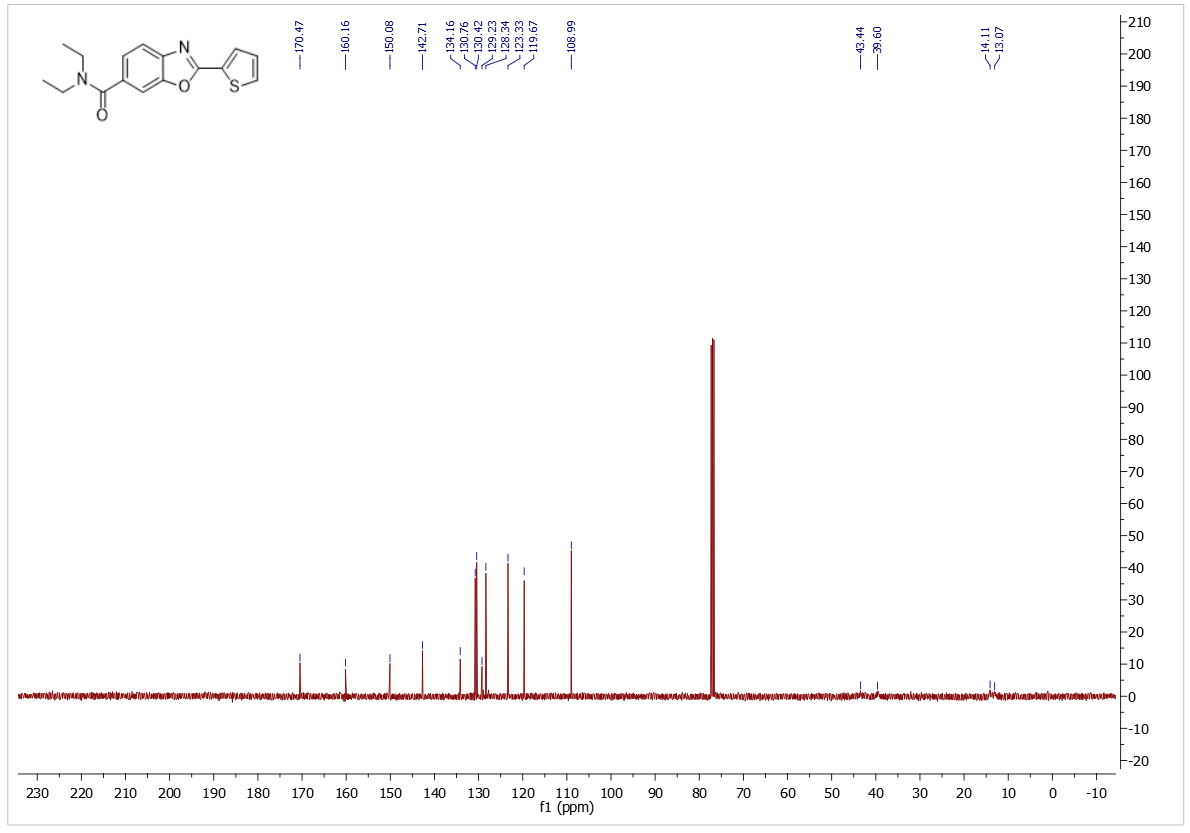


**Figure S76.** ^13^C-NMR Spectrum of compound **44**


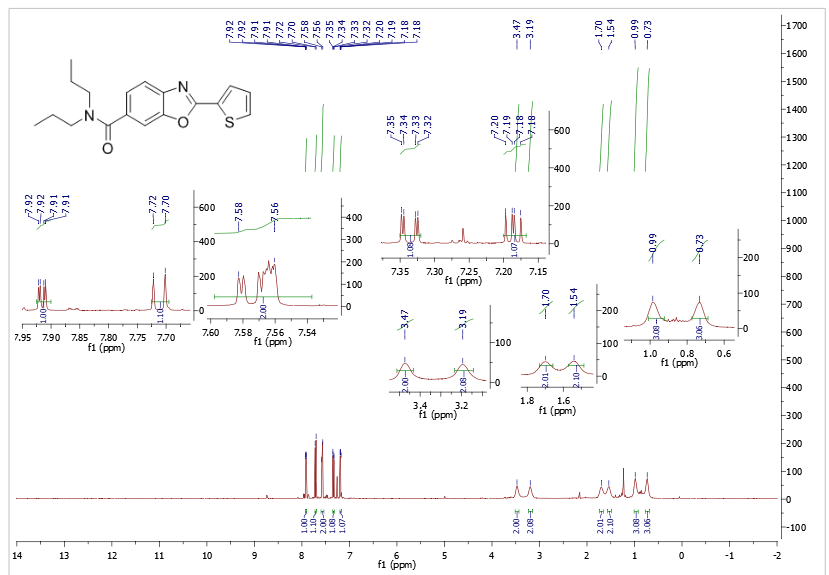


**Figure S77.** ^1^H-NMR Spectrum of compound **45**


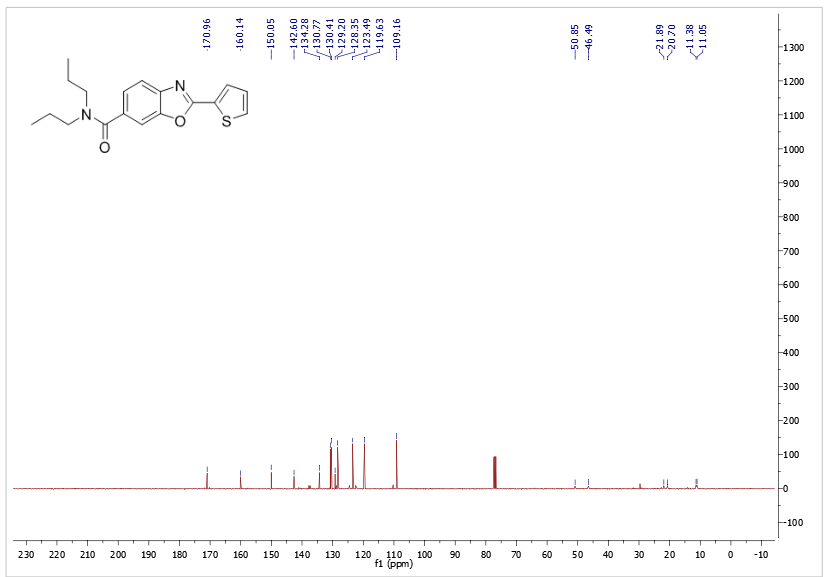


**Figure S78.** ^13^C-NMR Spectrum of compound **45**


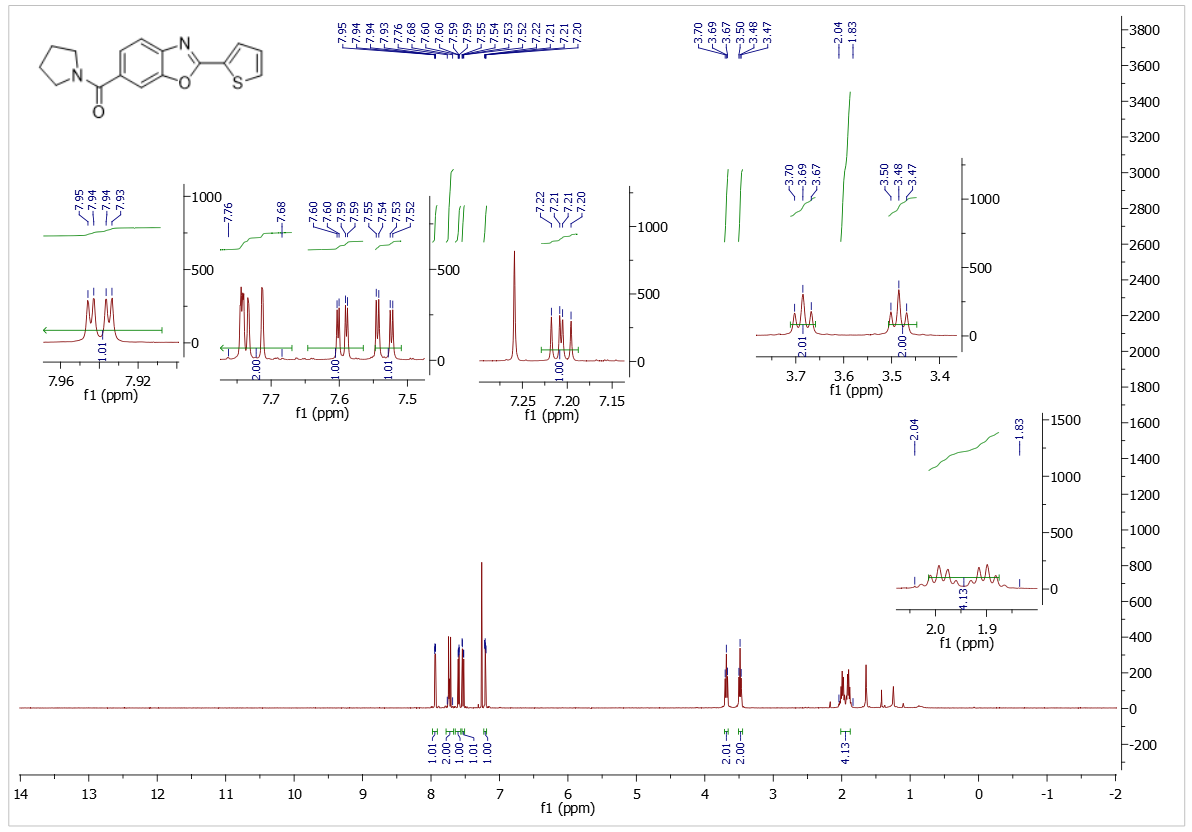


**Figure S79.** ^1^H-NMR Spectrum of compound **46**


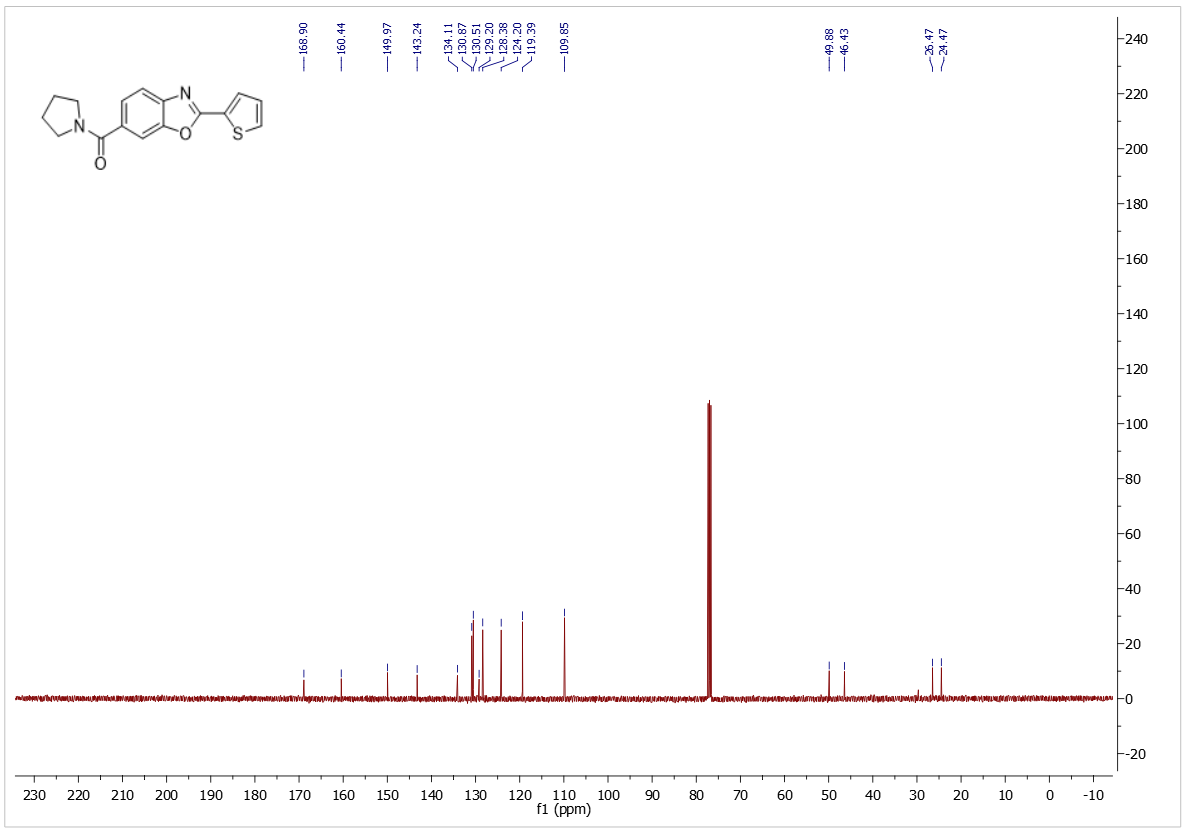


**Figure S80.** ^13^C-NMR Spectrum of compound **46**


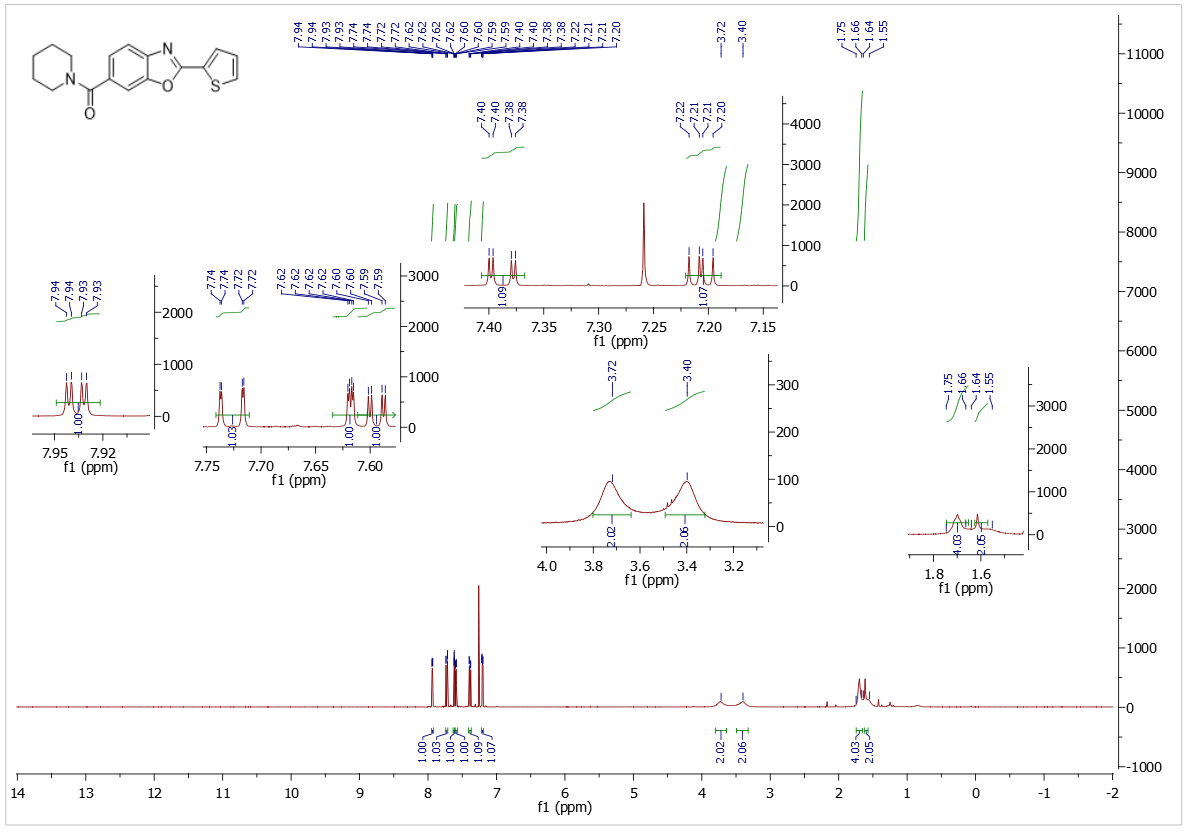


**Figure S81.** ^1^H-NMR Spectrum of compound **47**


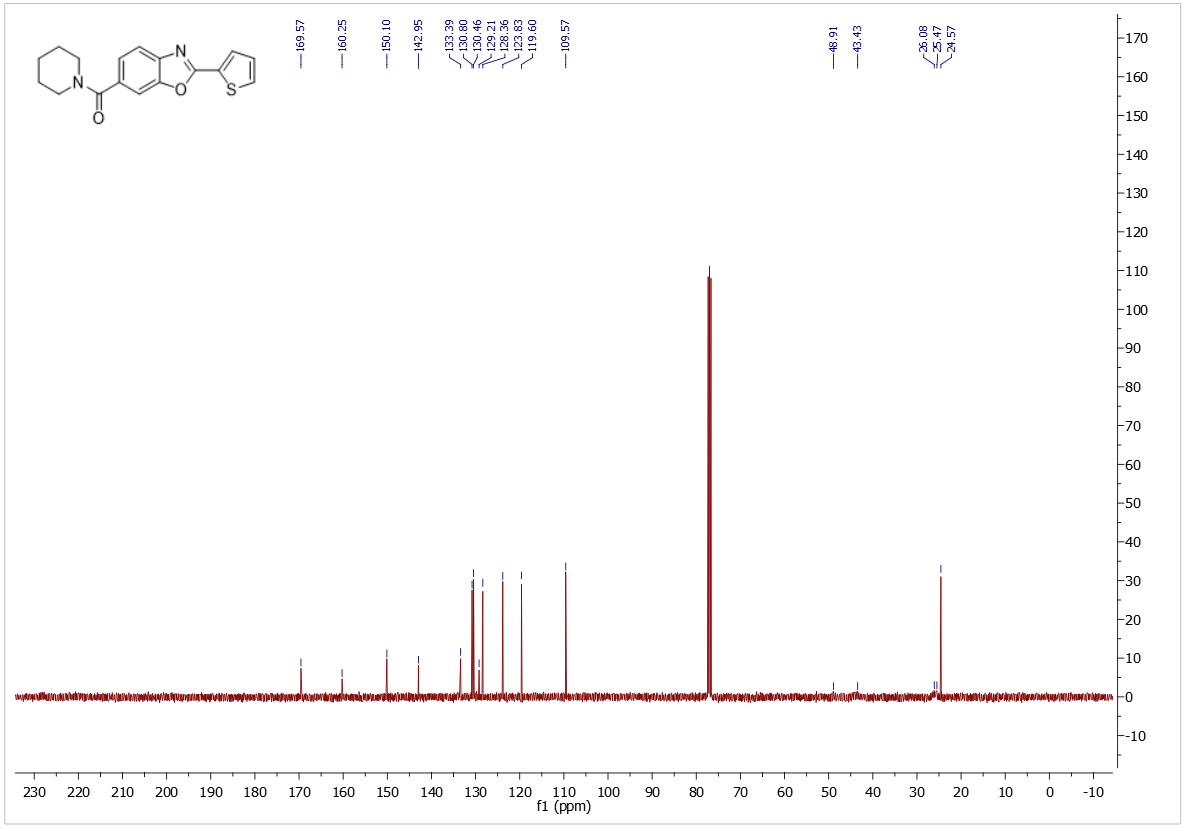


**Figure S82.** ^13^C-NMR Spectrum of compound **47**


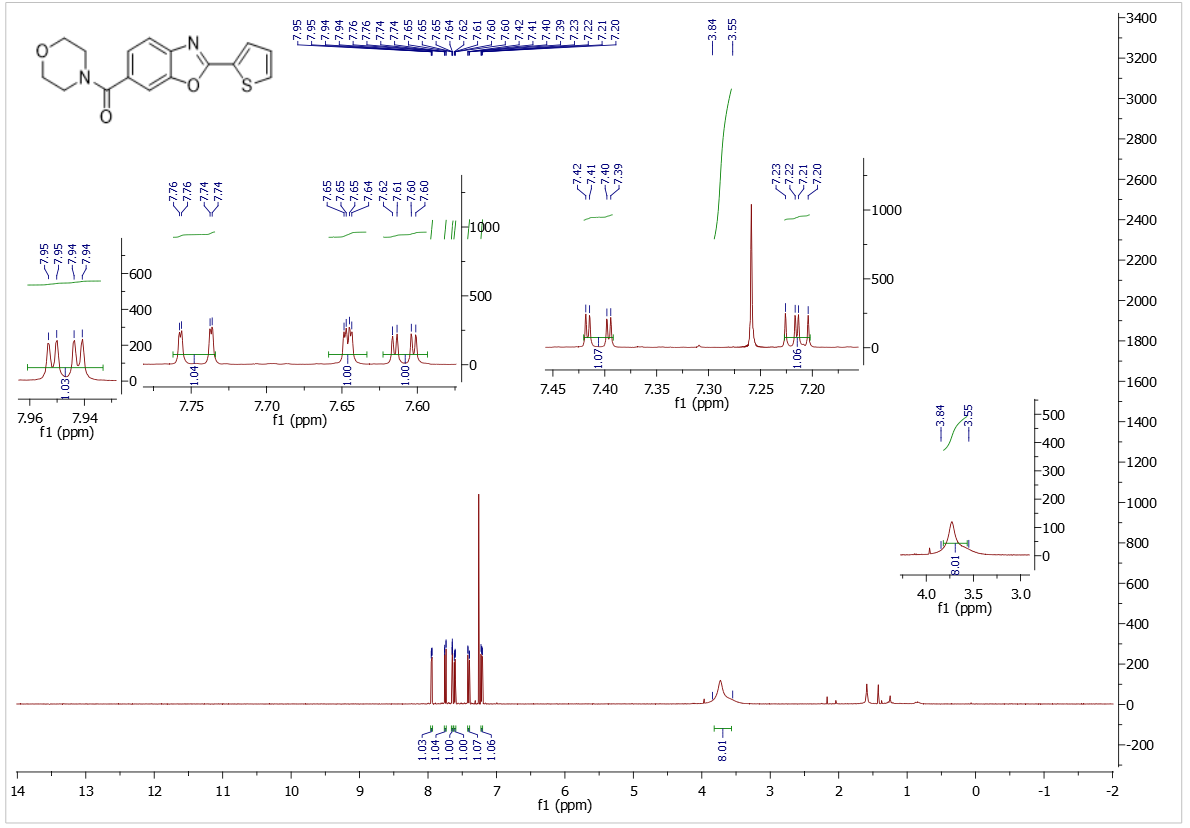


**Figure S83.** ^1^H-NMR Spectrum of compound **48**


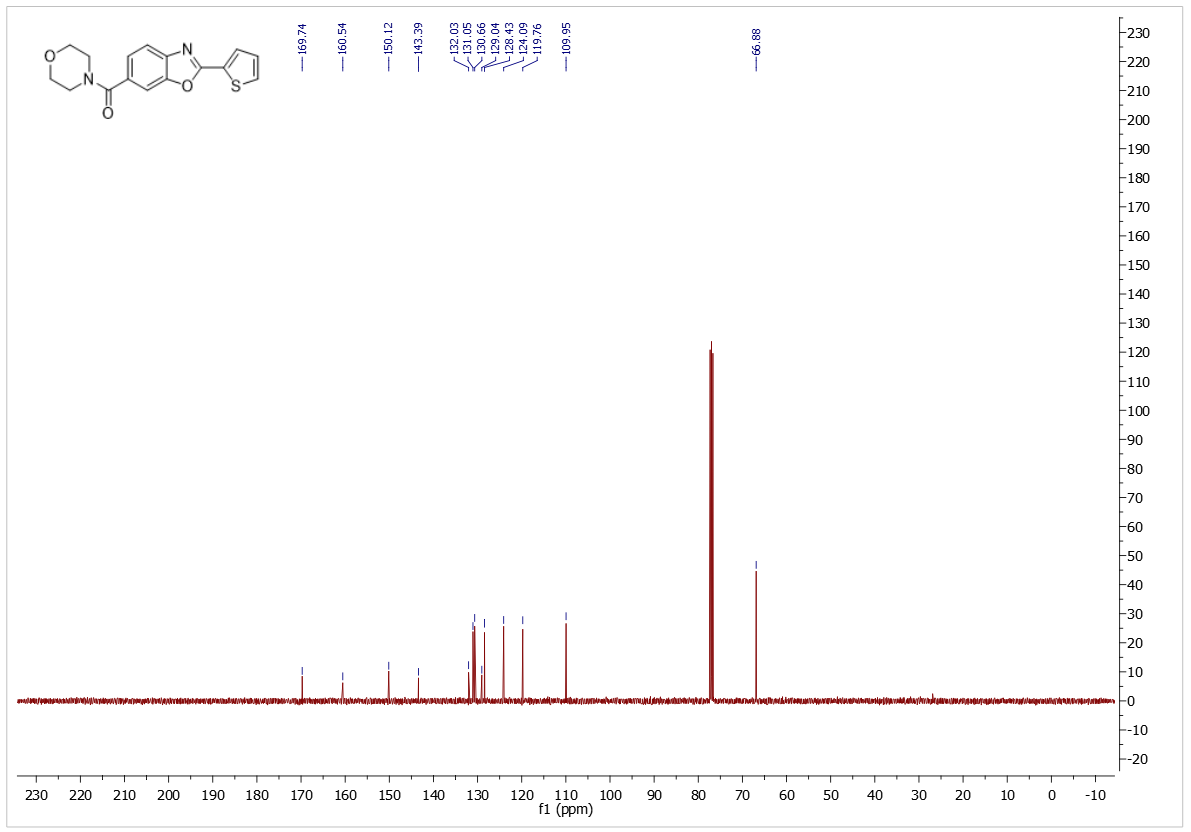


**Figure S84.** ^13^C-NMR Spectrum of compound **49**

1. **QTOF spectrum copies of compounds 7-48**


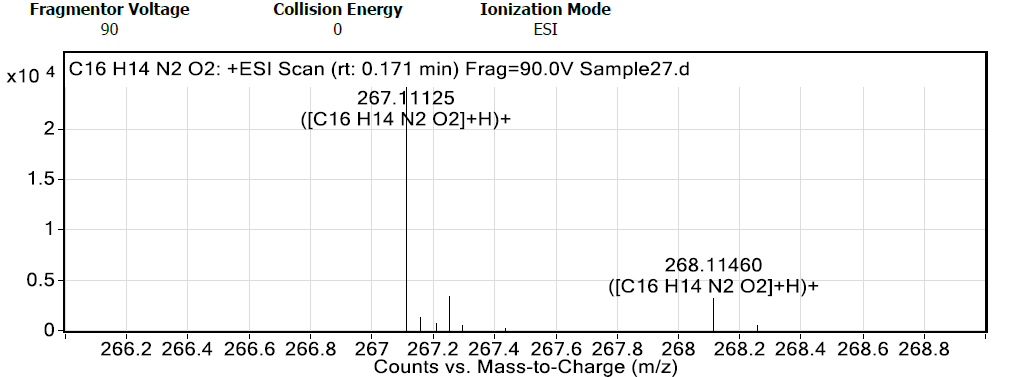


**Figure S85.** QTOF Spectrum of compound **7**


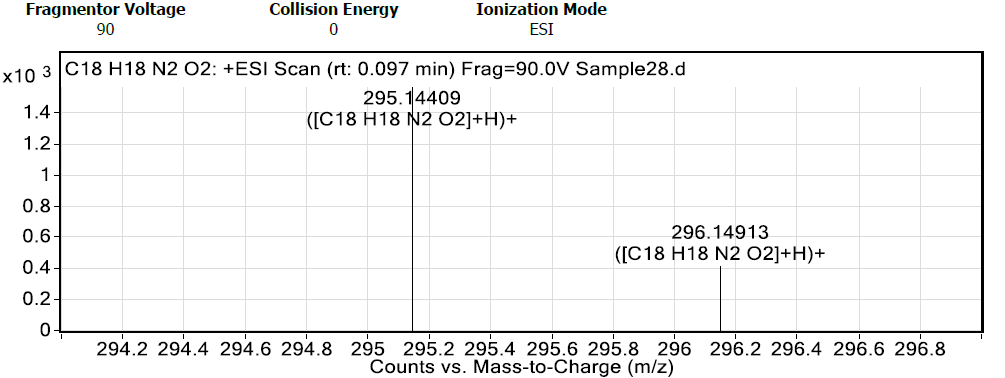


**Figure S86.** QTOF Spectrum of compound **8**


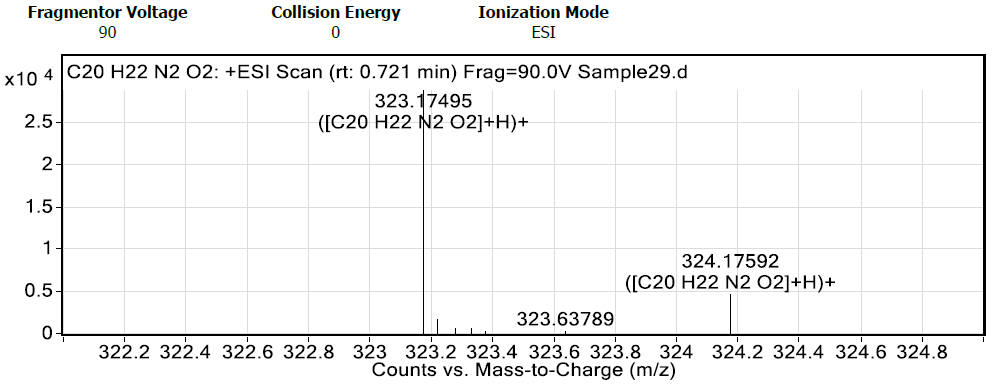


**Figure S87.** QTOF Spectrum of compound **9**


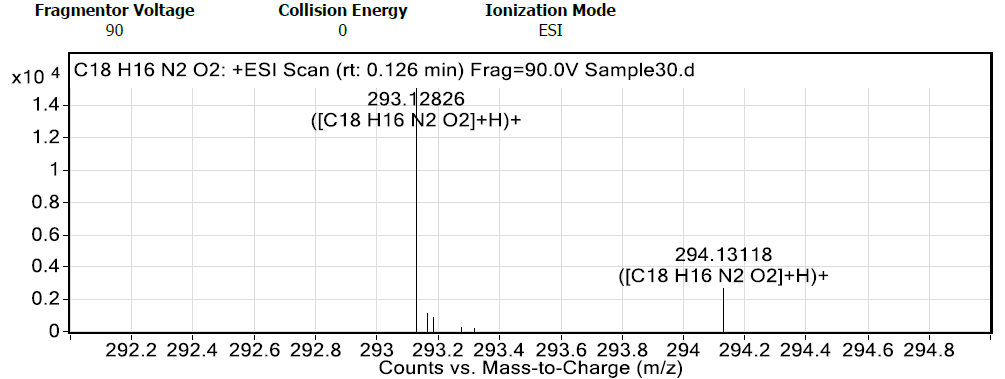


**Figure S88.** QTOF Spectrum of compound **10**


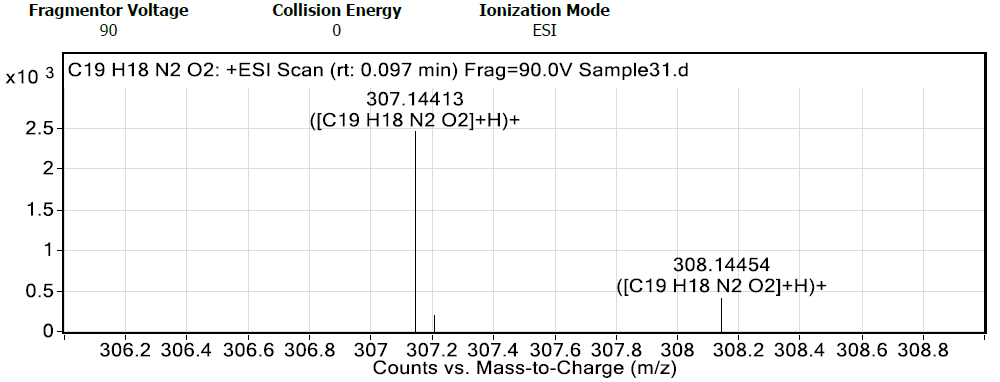


**Figure S89.** QTOF Spectrum of compound **11**


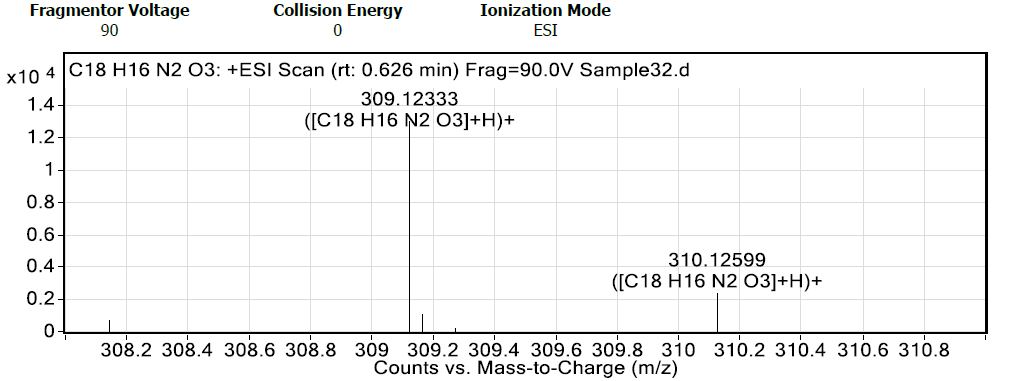


**Figure S90.** QTOF Spectrum of compound **12**


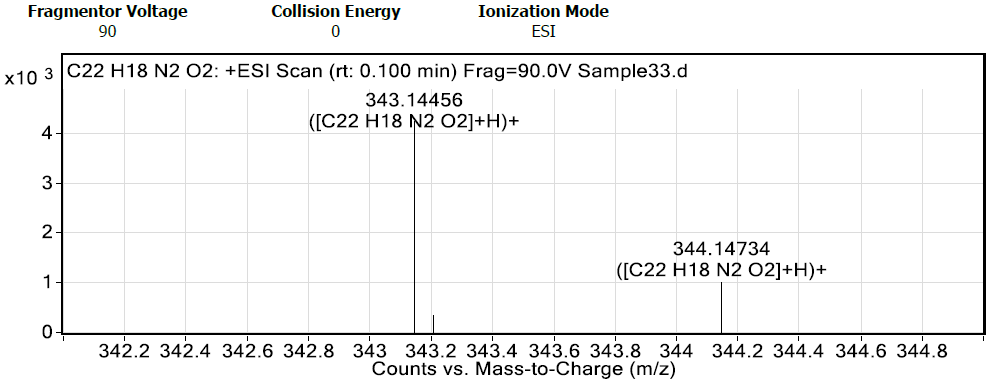


**Figure S91.** QTOF Spectrum of compound **13**


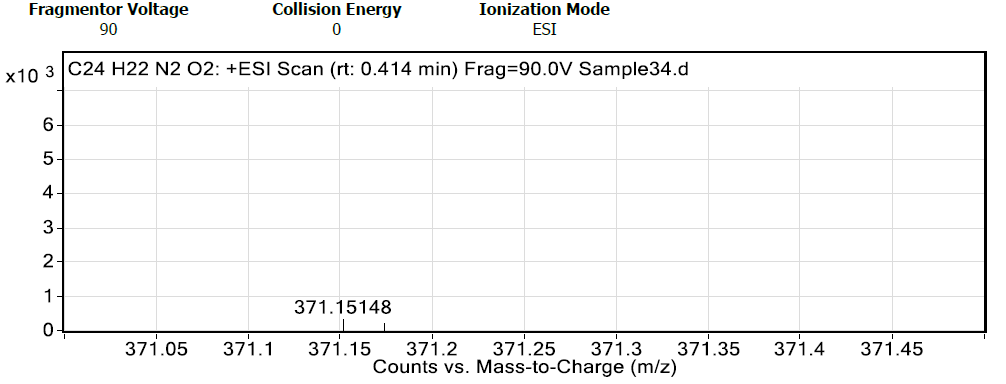


**Figure S92.** QTOF Spectrum of compound **14**


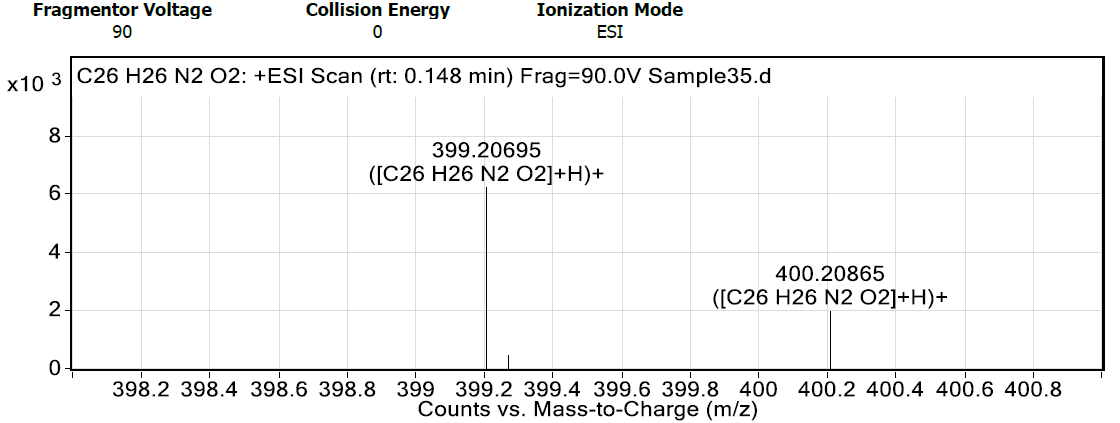


**Figure S93.** QTOF Spectrum of compound **15**


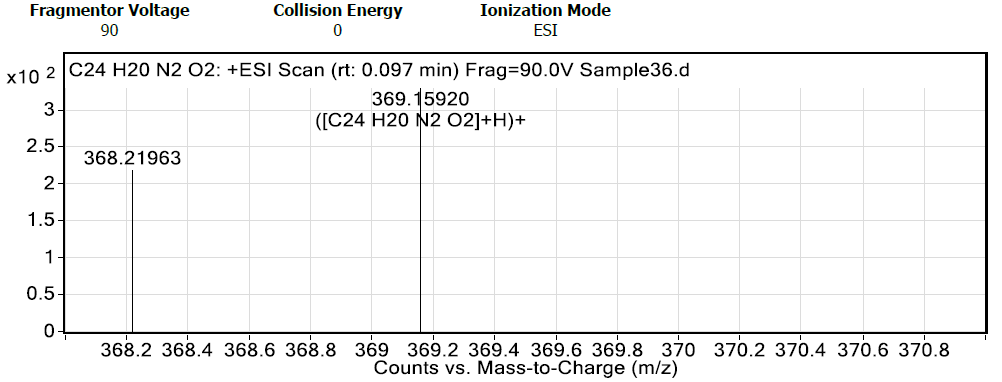


**Figure S94.** QTOF Spectrum of compound **16**


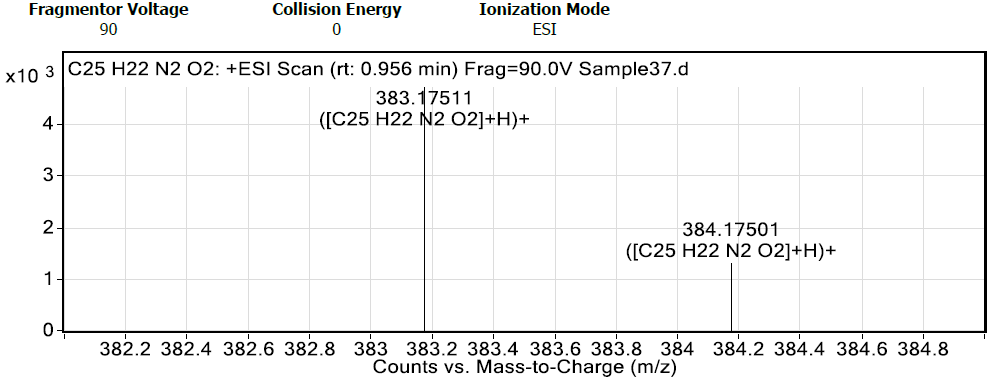


**Figure S95.** QTOF Spectrum of compound **17**


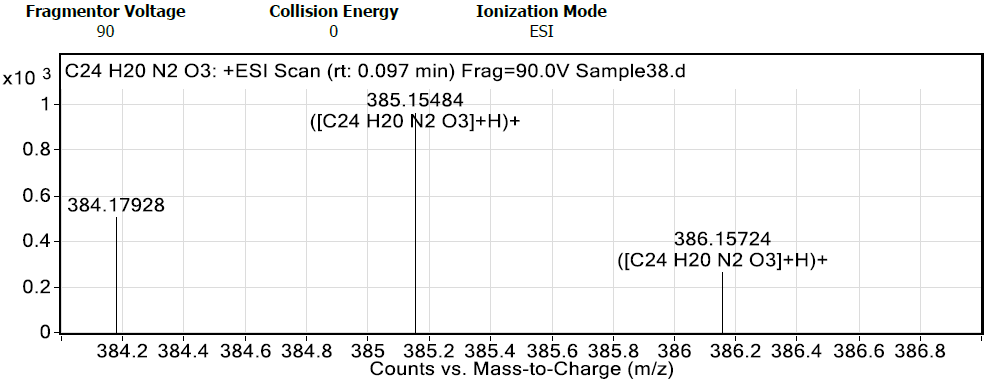


**Figure S96.** QTOF Spectrum of compound **18**


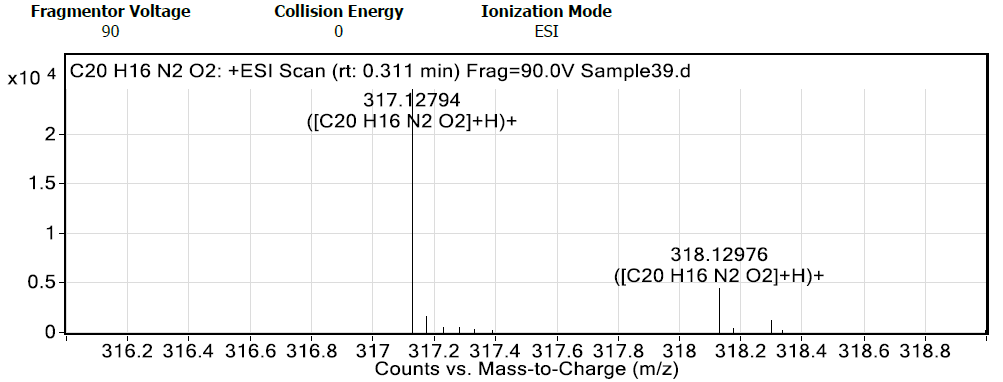


**Figure S97.** QTOF Spectrum of compound **19**


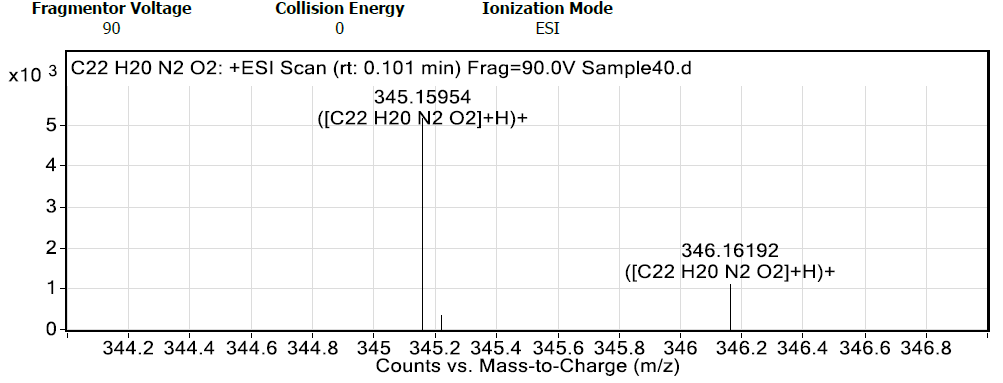


**Figure S98.** QTOF Spectrum of compound **20**


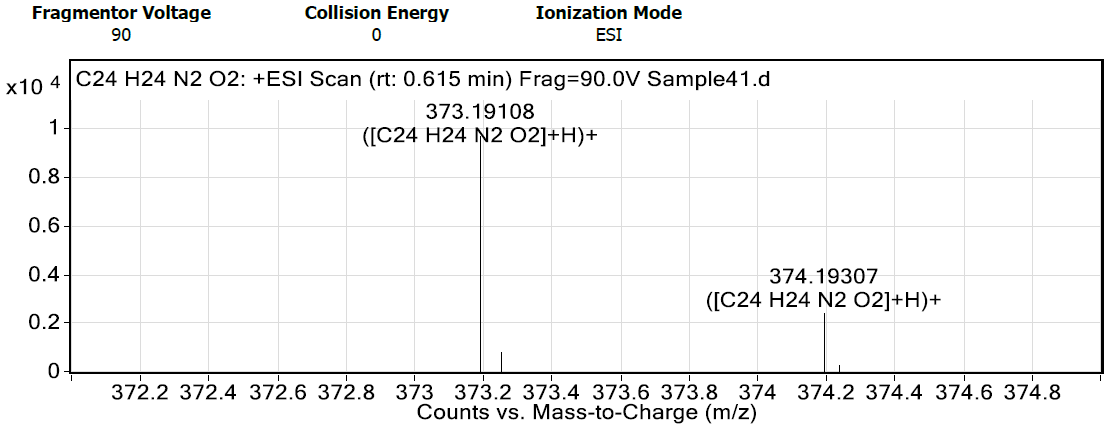


**Figure S99.** QTOF Spectrum of compound **21**


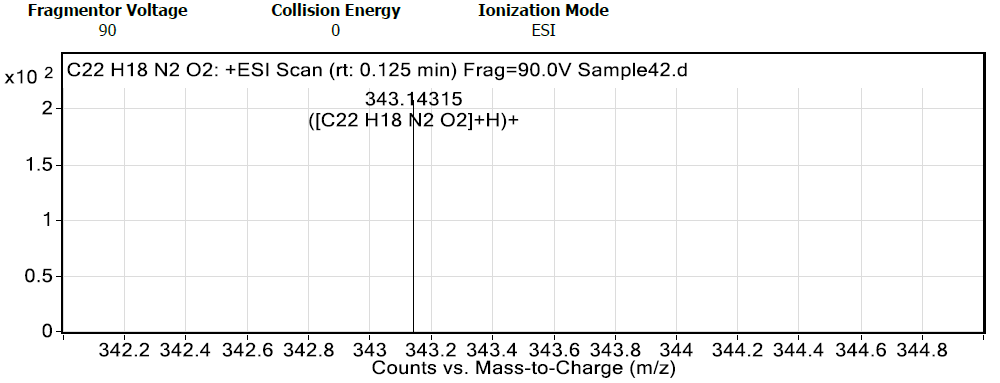


**Figure S100.** QTOF Spectrum of compound **22**

**Figure S101.** QTOF Spectrum of compound **23**

**Figure S102.** QTOF Spectrum of compound **24**

**Figure S103.** QTOF Spectrum of compound **25**

**Figure S104.** QTOF Spectrum of compound **26**

**Figure S105.** QTOF Spectrum of compound **27**

**Figure S106.** QTOF Spectrum of compound **28**

**Figure S107.** QTOF Spectrum of compound **29**

**Figure S108.** QTOF Spectrum of compound **30**

**Figure S109.** QTOF Spectrum of compound **31**

**Figure S110.** QTOF Spectrum of compound **32**

**Figure S111.** QTOF Spectrum of compound **33**

**Figure S112.** QTOF Spectrum of compound **34**

**Figure S113.** QTOF Spectrum of compound **35**

**Figure S114.** QTOF Spectrum of compound **36**

**Figure S115.** QTOF Spectrum of compound **37**

**Figure S116.** QTOF Spectrum of compound **38**

**Figure S117.** QTOF Spectrum of compound **39**

**Figure S118.** QTOF Spectrum of compound **40**

**Figure S119.** QTOF Spectrum of compound **41**

**Figure S120.** QTOF Spectrum of compound **42**

**Figure S121.** QTOF Spectrum of compound **43**

**Figure S122.** QTOF Spectrum of compound **44**

**Figure S123.** QTOF Spectrum of compound **45**

**Figure S124.** QTOF Spectrum of compound **46**

**Figure S125.** QTOF Spectrum of compound **47**

**Figure S126.** QTOF Spectrum of compound **48**

1. **Molecular Dynamics trajectory frame of compound 36 and Fraction of residue interactions for AChE and BChE**

**Figure S127.** Fraction of residue interactions obtained during MD stimulation with **36** AChE complex.

**Figure S128.** Compound 36 interaction with the residues in each trajectory frame in AChE

**Figure S129.** Fraction of residue interactions obtained during MD stimulation with **36** BChE complex.

**Figure S130.** Compound 36 interaction with the residues in each trajectory frame in BChE

1. **Molecular dynamic simulations for donepezil inside AChE and BChE**

**Figure S131.** RMSD plot of 100ns molecular dynamic simulations for donepezil inside AChE.

**Figure S132.** RMSD plot of 100ns molecular dynamic simulations for donepezil inside BChE.

1. **Table S1. Some predicted pharmacokinetic properties of compounds 7-48**

| **Comp.** | **QPlogPo/w^a^** | **CNS^b^** | **QlogBB^c^** | **QPPMDCK^d^** | **% Human oral absorption^e^** | **BBB permeation^f*^** | **Rule of Five^g*^** |
| --- | --- | --- | --- | --- | --- | --- | --- |
| **7** | 2,702 | 0 | -0,072 | 1407,99 | 100 | YES | 0 |
| **8** | 3,441 | 0 | -0,124 | 1712,25 | 100 | YES | 0 |
| **9** | 4,221 | 0 | -0,28 | 1694,763 | 100 | YES | 0 |
| **10** | 3,177 | 0 | -0,068 | 1467,989 | 100 | YES | 0 |
| **11** | 3,494 | 1 | 0,014 | 1776,389 | 100 | YES | 0 |
| **12** | 2,378 | 1 | 0,017 | 1713,25 | 100 | YES | 0 |
| **13** | 4,355 | 0 | -0,191 | 1405,712 | 100 | YES | 0 |
| **14** | 5,136 | 0 | -0,217 | 1796,597 | 100 | YES | 1 |
| **15** | 5,894 | 0 | -0,376 | 1730,565 | 100 | NO | 1 |
| **16** | 4,825 | 0 | -0,191 | 1451,282 | 100 | YES | 0 |
| **17** | 5,149 | 0 | -0,096 | 1775,38 | 100 | YES | 1 |
| **18** | 4,033 | 0 | -0,095 | 1714,698 | 100 | YES | 0 |
| **19** | 3,635 | 0 | -0,049 | 1554,501 | 100 | YES | 0 |
| **20** | 4,352 | 0 | -0,093 | 1917,355 | 100 | YES | 0 |
| **21** | 5,212 | 0 | -0,2 | 2094,41 | 100 | NO | 1 |
| **22** | 4,104 | 0 | -0,053 | 1590,785 | 100 | YES | 0 |
| **23** | 4,419 | 1 | 0,034 | 1930,412 | 100 | YES | 0 |
| **24** | 3,264 | 0 | -0,008 | 1658,819 | 100 | YES | 0 |
| **25** | 1,653 | 0 | -0,336 | 721,376 | 93,032 | YES | 0 |
| **26** | 2,381 | 0 | -0,395 | 875,033 | 100 | YES | 0 |
| **27** | 3,183 | 0 | -0,568 | 866,521 | 100 | YES | 0 |
| **28** | 2,124 | 0 | -0,345 | 743,199 | 96,001 | YES | 0 |
| **29** | 2,445 | 0 | -0,254 | 909,944 | 100 | YES | 0 |
| **30** | 1,329 | -1 | -0,242 | 878,236 | 92,552 | YES | 0 |
| **31** | 2,243 | 0 | -0,315 | 749,445 | 96,757 | YES | 0 |
| **32** | 2,896 | 0 | -0,36 | 905,247 | 100 | YES | 0 |
| **33** | 3,621 | 0 | -0,546 | 876,259 | 100 | YES | 0 |
| **34** | 2,69 | 0 | -0,317 | 780,655 | 100 | YES | 0 |
| **35** | 2,996 | 0 | -0,232 | 945,719 | 100 | YES | 0 |
| **36** | 2,042 | 0 | -0,221 | 910,628 | 96,982 | YES | 0 |
| **37** | 1,906 | 0 | -0,092 | 1253,818 | 100 | YES | 0 |
| **38** | 2,603 | 0 | -0,093 | 1693,021 | 100 | YES | 0 |
| **39** | 3,368 | 0 | -0,262 | 1639,252 | 100 | YES | 0 |
| **40** | 2,38 | 0 | -0,089 | 1309,583 | 100 | YES | 0 |
| **41** | 2,695 | 0 | -0,01 | 1581,522 | 100 | YES | 0 |
| **42** | 1,581 | 0 | -0,005 | 1525,066 | 100 | YES | 0 |
| **43** | 2,598 | 1 | 0,027 | 2418,762 | 100 | YES | 0 |
| **44** | 3,333 | 1 | 0,02 | 3309,051 | 100 | YES | 0 |
| **45** | 4,087 | 0 | -0,149 | 3198,058 | 100 | NO | 0 |
| **46** | 3,069 | 1 | 0,025 | 2493,079 | 100 | YES | 0 |
| **47** | 3,389 | 1 | 0,114 | 3052,884 | 100 | YES | 0 |
| **48** | 2,273 | 1 | 0,116 | 2939,772 | 100 | NO | 0 |

^a^ Predicted octanol/water partition coefficient log P (recommended value: −2.0 – 6.5).

^b^ Predicted central nervous system activity on a –2 (inactive) to +2 (active) scale.

^c^ Predicted brain/blood partition coefficient (recommended value: -3.00 – 1.20).

^d^ Predicted apparent MDCK cell permeability in nm/*sec* (<25 poor, >500 great).

^e^ Percentage of human oral absorption (<25% is weak and > 80% is strong).

^f^ Blood/brain barrier permeation.

^g^ Number of violations of Lipinski’s rule of five (maximum is 4).

*: Calculated with SwissAdme.

1. **References**
2. Kuzu, B., Sari, O., Erdem, S. S., Algul, O., & Menges, N. (2021). Synthesis of Benzoxazole‐2‐carboxylate Derivatives: Electronic‐and Position‐effect of Functional Groups and Computational Modeling of the Selectivity for Oxazole Ring. ChemistrySelect, 6(10), 2529-2538.
3. Kuzu, B., Hepokur, C., Turkmenoglu, B., Burmaoglu, S., & Algul, O. (2022). Design, synthesis and in vitro antiproliferation activity of some 2-aryl and-heteroaryl benzoxazole derivatives. Future Medicinal Chemistry, 14(14), 1027-1048.
4. Kuzu, B., Ayaz, F., & Algul, O. (2019). Synthesis of new alicyclic oxalamide derivatives and their differential immunomodulatory activities on the mammalian cells. Journal of Heterocyclic Chemistry, 56(7), 1946-1952.
